# Telescoping a Prenyltransferase and a Diterpene Synthase to Transform Unnatural FPP Derivatives to Diterpenoids

**DOI:** 10.1021/acs.orglett.4c01670

**Published:** 2024-07-08

**Authors:** Henry Struwe, Heng Li, Finn Schrödter, Laurent Höft, Jörg Fohrer, Jeroen S. Dickschat, Andreas Kirschning

**Affiliations:** † Institute of Organic Chemistry, Leibniz University Hannover, Schneiderberg 1B, 30167 Hannover, Germany; ‡ Kekulé-Institute of Organic Chemistry and Biochemistry, University of Bonn, Gerhard-Domagk-Straße 1, 53121 Bonn, Germany; § Department of Chemistry, Technical University Darmstadt, Alarich-Weiss-Straße 4, 64287 Darmstadt, Germany; ∥ Uppsala Biomedical Center (BMC), University Uppsala, Husargatan 3, 752 37 Uppsala, Sweden

## Abstract

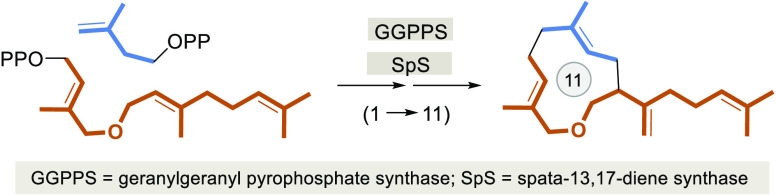

New diterpenoids are accessible from non-natural FPP derivatives as substrates for an enzymatic elongation cyclization cascade using the geranylgeranyl pyrophosphate synthase (GGPPS) from *Streptomyces cyaneofuscatus* and the spata-13,17-diene synthase (SpS) from *Streptomyces xinghaiensis*. This approach led to four new biotransformation products including three new cyclododecane cores and a macrocyclic ether. For the first time, a 1,12-terpene cyclization was observed when shifting the central olefinic double bond toward the geminial methyl groups creating a nonconjugated 1,4-diene.

Terpenoids are formed by a highly modular and iterative process starting from two C_5_ building blocks, namely, the nucleophilic starter unit isopentenyl pyrophosphate (**2**, IPP) and the electrophilic dimethylallyl pyrophosphate (**1**, DMAPP).^1^ The iterative elongation of DMAPP (**1**) and higher oligoprenyl pyrophosphates with IPP (**2**) is promoted by prenyltransferases.^1^ Successive rounds of elongations first lead to geranyl pyrophosphate (GPP), the precursor of monoterpenes (C_10_), and then to farnesyl pyrophosphate (FPP), geranylgeranyl pyrophosphate (**3**, GGPP), and geranylfarnesyl pyrophosphate (GFPP). From there, a cationic cascade that involves the established repertoire of carbocation chemistry is induced by class I or II terpene synthases (TSs).^2^ The final carbocations that originate from these cascades are either trapped by deprotonation or nucleophilically by water. As a result, oligocyclic terpenoids such as spata-13,17-diene (**4**) are formed, which is the product of spata-13,17-diene synthase (SpS) from *Streptomyces xinghaiensis*.^3^ Tailoring enzymes including oxygenases and acyl transferases then provide the final biosynthetic products. In the case of the oxidized diterpene spatol (**5**),^4^ known from different brown algae, the skeleton bears the opposite absolute configuration of that of the product formed by SpS (Scheme 1A).^5^

**Scheme 1:**
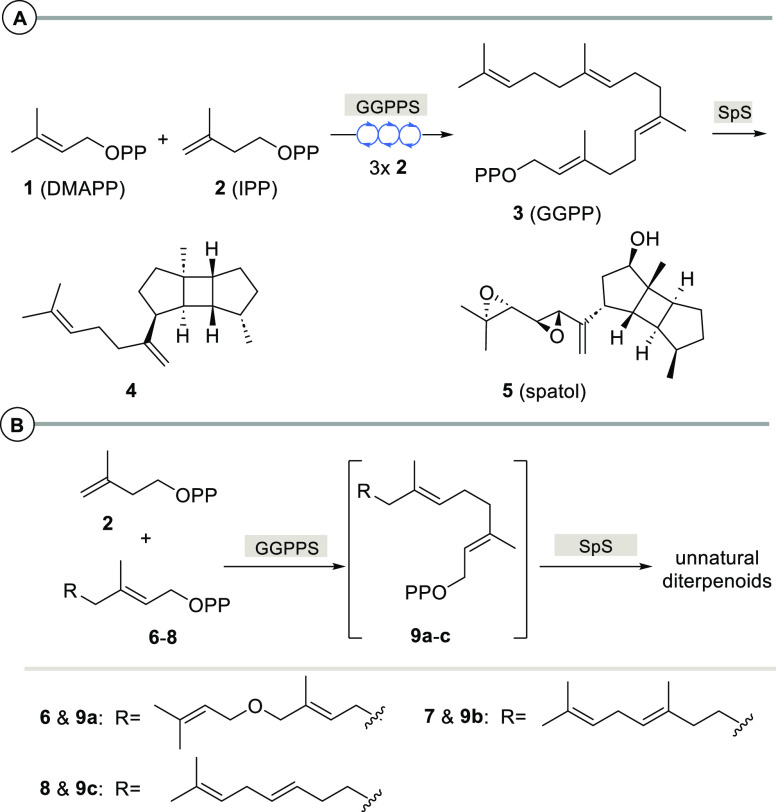
(A) Overview on the Biosynthesis of Spata-13,17-diene (**4**) from C_5_ Precursors DMAPP (**1**) and IPP (**2**) via GGPP (**3**) and Structure of Spatol (**5**) and (B) Elongation of FPP Derivatives **6**–**8** and Cyclization of Intermediate GGPP Derivatives **9a**–**9c** Reported in This Work

With the exception of noncanonical enzymes,^6−9^ the natural substrates of TSs are the aforementioned acyclic pyrophosphates, but TSs are also known to exhibit a remarkable substrate tolerance,^10^ accepting not only halogenated terpenoid precursors^11−13^ but also oxygenated compounds^14−16^ or (thio)ether derivatives.^17^ If the terpene precursors exhibit altered methylation patterns^18,19^ or shifted double bonds,^20^ new reaction pathways lead to novel terpenes with previously unknown backbones.

In contrast, the substrate promiscuity of elongating enzymes such as GGPP have not been probed to the same extent. Here, we provide the first examples of using unnatural FPP derivatives in a cascaded biotransformation that is based on the GGPP synthase (GGPPS) from *Streptomyces cyaneofuscatus*(21) and the diterpene synthase SpS thereby probing the substrate scope of two enzymes. For this purpose, the non-natural FPP derivatives **6**–**8** were chosen (Scheme 1B).

While the syntheses of FPP ether derivative **6**(17) as well as FPP derivative **8**(22) with a shifted central olefinic double bond have been reported before, the *nor-*FPP derivative **7** is a so far unknown substrate. Its synthesis commenced from (*E*)-hex-3-enedioic acid (**10**; Scheme 2). Esterification, reduction, and selective monoprotection of one alcohol provided silylether **11**. Dess-Martin oxidation, Wittig olefination, and *O*-desilylation provided allyl alcohol **12**. A two-step protocol initiated by Appel bromination followed by introduction of the phenylsulfonyl group yielded sulfone **13**. The lithiated derivative was next alkylated with known bromide **14**,^17^ and the resulting sulfone **15** was reductively desulfonylated. Finally, the terminal alcohol was liberated, and based on an established protocol reported by Poulter et al. the target pyrophosphate **7** was collected as trisammonium salt.^23^

**Scheme 2:**
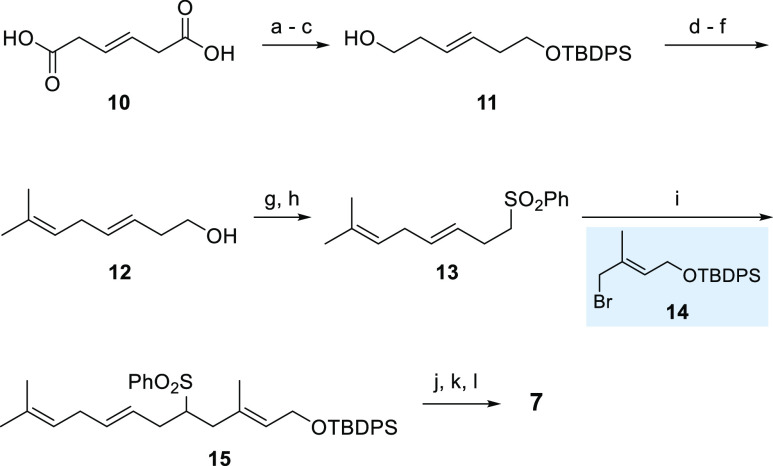
Synthesis of FPP Derivative **7** Reagents and conditions: (a) H_2_SO_4_, MeOH, Δ (90%); (b) DIBAL-H, THF, −78 °C (96%); (c) TBDPSCl, imidazole, *N*,*N*-DMF, rt (67%); (d) Dess-Martin periodinane, THF, 0 °C to rt; (e) *i*-PrPPh_3_I, *n*-BuLi, THF, 0 °C to rt to 40 °C; (f) TBAF, THF, 0 °C to rt (50% over three steps); (g) CBr_4_, PPh_3_, CH_2_Cl_2_, 0 °C to rt (quant.); (h) NaSO_2_Ph, *N*,*N*-DMF, 50 °C to rt (60%); (i) *n*-BuLi, THF, bromide **14**, −78 °C to rt (80%); (j) Na_2_HPO_4_, Na–Hg, THF, MeOH, −14 °C to rt (95%); (k) TBAF, THF, 0 °C to rt (91%); (l) Et_3_N, MsCl, LiCl, THF, 0 °C, then ((*n*-Bu)_4_N)_3_P_2_O_7_H, MeCN, rt (91% over two steps).

The synthetic FPP derivatives **6**–**8** (50 μg for screening) were elongated with IPP and cyclized using a mixture of recombinant purified GGPPS and SpS. Product formation was detected by GC-MS. After having confirmed successful enzyme transformations, the biotransformations were upscaled to conversions of **6** (45 mg), **7** (46 mg), and **8** (119 mg) with the addition of appropriate amounts of IPP and enzymes (cf. SI for details), followed by product isolation. While SpS naturally catalyzes an initial 1,10-cyclization, as a consequence of the inserted oxygen atom in **6** or the shifted double bond in **7** and **8**, respectively, in all three cases, new cyclization modes toward 11- or 12-membered macrocyclic terpenoids were observed (Scheme 3).

**Scheme 3:**
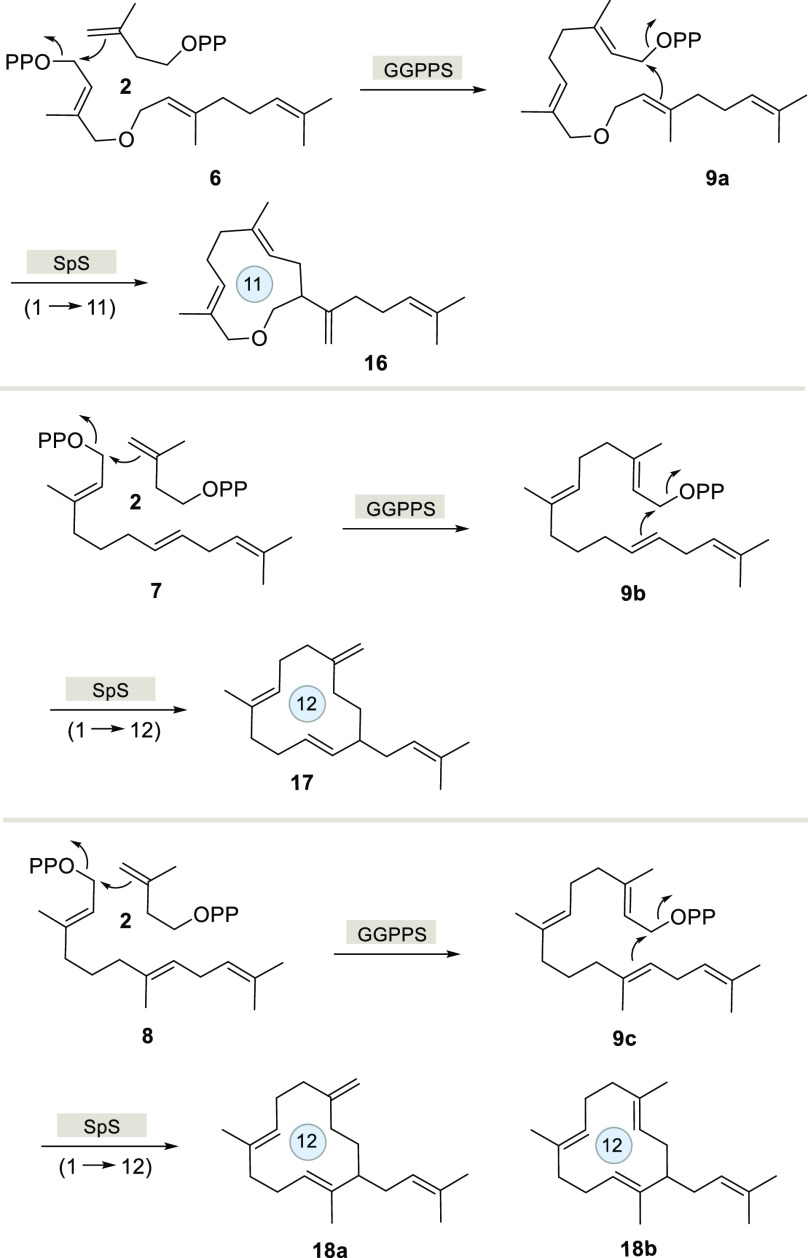
Overview on Biotransformations (Ring Sizes Are Labelled)

The structure elucidation of the new terpenoids was based on NMR spectroscopy relying on the key H,H COSY and HMBC correlations summarized in Scheme 4. The number of alkene groups present in macrocyclic ether **16** proved that the product was the result of only one cyclization step. Chemical shifts (δ = 1.67 and 1.59 ppm, respectively) and multiplicities (both singlets) of the germinal methyl groups in **16** suggest that they are allylic in nature. Also a conformational analysis of **16** was conducted in a similar way to that reported for germacrenes by conducting 1D-NOE experiments (Scheme 4 and SI).^18,24^

**Scheme 4:**
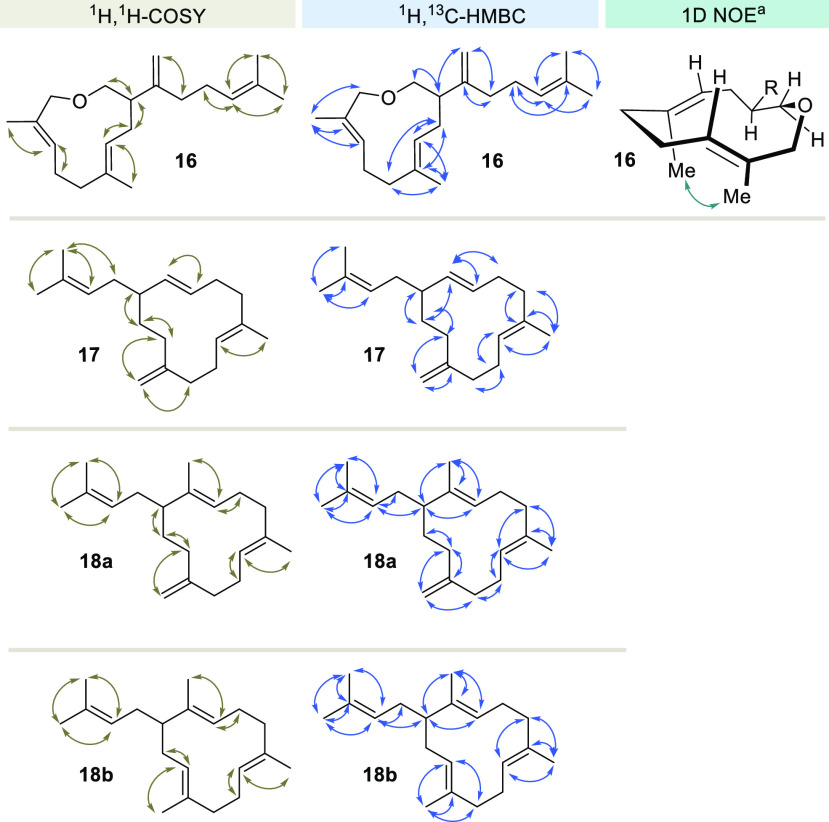
Details on the Structure Elucidation of Macrocycles **16**–**18** by NMR Analysis 1D NOE correlation between methyl groups (δ= 1.44 ppm and δ= 1.59 ppm); the alternate conformation with both methyl groups pointing upward not shown.

Peak broadening in the NMR spectra especially for macrocyclic ether **16**, a frequently observed phenomenon for conformationally flexible but strained terpenes,^25^ posed a challenge for the structure elucidation here. In particular, the ^13^C signal intensities for the carbon atom bearing the stereogenic center as well as for the carbon atoms bound to the ether oxygen atom in **16** were reduced. The 2D data provided some support for achieving a preliminary assignment. Enhanced signal intensities were ultimately obtained when NMR measurements were conducted at an elevated temperature (*T*_NMR_ = 310.0 K; see SI).

Macrocyle **17** provided well resolved NMR spectra so that structure elucidation was straightforward. Compounds **18a** and **18b** were isolated as an inseparable mixture (ratio 1:1.1), which complicated structure elucidation (for details, see SI).

Obviously successful elongation of the FPP derivatives **6**–**8** by GGPPS as well cyclization of intermediates **9a**–**9c** with SpS had occurred. The formation of **16** is explained by a 1,11-cyclization and deprotonation (Scheme 3). Also the biotransformation to **18b** requires just a simple 1,12-cyclization and deprotonation from C10 (Scheme 5, loss of H_a_).

**Scheme 5:**
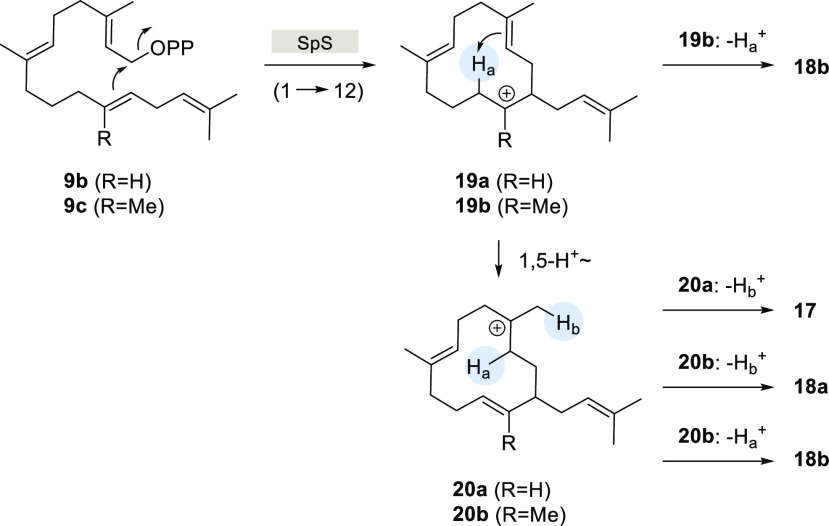
Proposed Mechanisms That Lead to Macrocycles **17** and **18a** with Shifted Double Bond

In contrast, the formation of **17** from **9b** and of **18a** from **9c** showed a surprising shift of the C2–C3 double bond of GGPP to a semicyclic double bond. For **9b**, this is explainable by a 1,12-cyclization to the secondary cation **19a** that may be a highly transient species (Scheme 5). A 1,5-hydrogen shift of H_a_ leads to a more stable tertiary cation **20a** that yields **17** upon deprotonation (loss of H_b_). Experimental and/or theoretical evidence for proton transfers during terpene cyclizations as proposed here has been reported previously for various terpene synthases including *inter alia* taxadiene synthase,^26−28^ fusicoccadiene synthase,^29^ trichodiene synthase,^30^ sodorifen synthase,^31^ and spiroluchuene A synthase.^32^ Interestingly, with substrate **9c**, the initial 1,12-cyclization, despite resulting in the tertiary cation **19b**, shows the same 1,5-proton shift of H_a_ to **20b**, explaning the formation of **18a** through deprotonation from Me20. An alternative explanation for the biosynthesis of **18b** is a deprotonation from **20b** with a loss of H_a_. However, since the formation of **18b** either from **19b** or from **20b** may proceed with a loss of the same proton H_a_, it may be difficult to distinguish experimentally between these two scenarios.

Although kinetics have not yet been determined in detail, it can be assumed that GGPP synthase is a slow-acting enzyme, particularly when a terpene synthase is not present. The product GGPP **3** is probably reluctant to leave the enzyme’s active site if cooperation with a terpene synthase, either through direct contact or via the medium, is lacking, since GGPP **3** itself is not essential for the cell. In contrast, the shorter isoprenoid FPP is used for triterpene biosynthesis and is therefore more essential for metabolism than **3**.

In conclusion, we demonstrated for the first time that geranylgeranyl pyrophosphate synthases exert substrate promiscuity with respect to farnesyl pyrophosphate derivatives and that the resulting new GGPP derivatives are directly transformed into macrocyclic diterpenoids by the dedicated diterpene synthase SpS. The one-pot telescoping using two enzymes as developed here demonstrates the great synthetic potential of these enzyme classes and paves the way to enlarge the number of diterpene backbones considerably.

## Data Availability

The data underlying this study are available in the published article and its Supporting Information.

## Supporting Information Available

Detailed procedures and spectral data (PDF)
