# Supplementary material for: Telescoping a Prenyltransferase and a Diterpene Synthase to Transform Unnatural FPP Derivatives to Diterpenoids
Source: Org Lett. 2024 Jul 8;26(28):5888–92. doi: 10.1021/acs.orglett.4c01670 (PMC11267608; doi:10.1021/acs.orglett.4c01670)
Supplement: Supplementary file 1 — ol4c01670_si_001.pdf [file ol4c01670_si_001.pdf]

Telescoping a prenyltransferase and a diterpene synthase to transform unnatural FPP derivatives to diterpenoids

Henry Struwe,[a#] Heng Li,[b#] Finn Schrödter,[a] Laurent Höft,[a] Jörg Fohrer,[c] Jeroen S. Dickschat*[b], Andreas Kirschning*[a,d]

[a] Institute of Organic Chemistry, Leibniz University Hannover, Schneiderberg 1B, 30167 Hannover, Germany; E-mail: andreas.kirschning@oci.uni-hannover.de.

[b] Kekulé-Institute of Organic Chemistry and Biochemistry, University of Bonn, Gerhard Domagk-Straße 1, 53121 Bonn, Germany; E-mail: dickschat@uni-bonn.de

[c] Department of Chemistry, Technical University Darmstadt, Alarich-Weiss-Straße 4, 64287 Darmstadt, Germany

[d] Uppsala Biomedical Center (BMC), University Uppsala, Husargatan 3, 752 37 Uppsala, Sweden

Table of Contents

1. Materials and methods

1.1 General information
1.2 Chemical syntheses
1.3 Microbiological methods and biotransformations
1.4 GC-MS data
1.5 Structure elucidation of compound **16**
1.6 Structure elucidation of compound **17**
1.7 Structure elucidation of compound mixture **18a** and **18b**

2. Copies of NMR spectra

3. References (supporting information)

1. Materials and methods

1.1 General information

The experimental procedures described below, in which water is neither a reactant nor used as a solvent, were carried out in pre-dried flasks under an inert gas atmosphere. To ensure homogeneity, the reaction mixtures were stirred with a magnetic stirrer. The temperatures indicated refer to the bath vessels used. Temperatures of −78 °C were achieved by an acetone-dry ice mixture, −41 °C can be obtained by MeCN dry ice mixtures and 0 °C by a mixture of water and ice. The room temperature does not refer to a specific value, but varies with the ambient temperature. For varying temperatures oil baths, NaCl-ice mixtures or a cryostat were used.

Unless stated otherwise, dry solvents were used for each reaction in which a pre-dried flask was used. Tetrahydrofuran, dichloromethane, *N,N*-dimethylformamide, acetonitrile, and diethyl ether were obtained as dry solvents from a "Braun" solvent purification system. Deuterated solvents were obtained from Deutero GmbH.

Column chromatography was performed using silica gel obtained from Macherey-Nagel (particle size 40-63 µm). During chromatography pressure was applied and the used solvents/mixtures are listed in the corresponding experimental procedure. To perform TLC analysis pre-coated TLC sheets ALUGRAM® Xtra SIL G/UV_{254} foil from Macherey-Nagel (layer: 0.20 mm silica gel 60 with fluorescent indicator UV_{254}) were used. As stain solutions reagents containing vaniline or $KMnO_4$ were used. In selected cases UV light (λ = 254 nm/ 366 nm) was employed to identify TLC spots. The R_f values given are subject to certain inaccuracies due to measurement limitations, but can be used for initial orientation. Volumes given as a sum refer to the amount of solvent used for the first solution and the additional solvent used for rinsing the glassware (e.g. solvent (2 mL + 2 mL)).

NMR data (^{1}H, ^{13}C, ^{31}P, DEPT135, 2D-NMR) were recorded on the following spectrometers: Bruker AVANCE I (ν_L(^{1}H)= 400 MHz) equipped with a DUL probe, Bruker AVANCE III HD (ν_L(^{1}H)=400 MHz) equipped with either a PRODIGY BBFO or BBO probe, Bruker AVANCE III HD (ν_L(^{1}H)=500 MHz) equipped with a TCI cryoprobe, and Bruker AVANCE NEO (ν_L(^{1}H)=600 MHz) equipped with DUL cryoprobe. All probes are equipped with z-Gradient coils. The deuterated solvents are given in the respective procedures ($CDCl_3$, C_6D_6, D_2O). The analysis was performed using the Bruker Topspin® software. The residual solvent signal of the deuterated solvents was used to calibrate the chemical shift scale of the NMR spectra. Chemical shifts δ are given in ppm, J coupling constants are given in Hz and were determined manually or with appropriate software functions. The abbreviations used for multiplicities are s (singlet), d (doublet), t (triplet), q (quartet), qi (quintet), and m (multiplet). When a particular signal cannot be unambiguously assigned to a particular position, e.g. because signals overlap, the indices in question are separated by "/" to indicate that there are several possibilities. Structural assignments were supported by collecting additional information from HSQC, COSY, HMBC and NOE NMR experiments.

HR-ESI-MS (Tof) analyzes were performed with Aliance 2695 HPLC (Waters) coupled to a LCT premier (Waters) with a lock spray dual ion ESI source. GC-EI-MS (quadrupole) was

measured on a 6890 GC (Agilent) / 5973 MSD (Hewlett Packard) (column: Optima WAX) and a 7890B GC (Agilent) / 5977B MSD (Agilent) (column: Optima5HT). HR-CI-MS (Tof) was performed with a 6890 GC + (Hewlett Packard) coupled to GCT Premier (Waters) equipped with a CI ion source. The GC systems are additionally equipped with a FI detector.

Ion exchange of tetra-*n*-butylammonium to ammonium cations was performed using DOWEX®50WX8 resin as column material. For this purpose, the resin was rinsed with an aqueous HCl solution (3 M) until the eluent was acidic (pH paper). It was then rinsed with H_2O to a near neutral pH, with NH_3 (6%) to an alkaline pH, and finally with ion exchange buffer (980 mL H_2O, 20 mL *i*-PrOH, 2 g NH_4HCO_3) to a pH of 8 to 9. The residues collected from the reactions were then uploaded onto the column and eluted with ion exchange buffer. The product-containing fractions, analyzed by $KMnO_4$-TLC staining, were combined and the solvent was removed in vacuo. The residue was diluted in an aqueous NH_4HCO_3 solution (0.05 M) and freeze-dried. To remove the inorganic ammonium pyrophosphate salt, the product was dissolved in an aqueous NH_4HCO_3 solution (0.05 M, 2 mL) and mixed with MeCN/*i*-PrOH (1/1, 8 mL). After centrifugation (5000 rpm, 10 min, 4 °C), the liquid was collected and the procedure was repeated. After removal of the solvent in vacuo, the residue was dissolved in an aqueous NH_4HCO_3 solution (0.05 M) and freeze-dried. The product was stored at temperatures between −70 °C and −80 °C. The diphosphate salts were analyzed by ^{1}H-, ^{13}C- and by ^{31}P-NMR spectroscopy. HRMS measurements and determination of R_f values could not be performed on these salts.

The syntheses of FPP derivatives **6** and **8** were reported in references S1 and S2.

Experiments with living microorganisms were performed in either S1 or S2 laboratories. Sterile work was performed using a Thermos Scientific laminar flow cabinet (type 2020). Optical density (OD600) was measured at 600 nm using a photometer from FoodALYT. Cell lysis was performed using a SONOPULS ultrasonic homogenizer from Bandelin (Procedure A) and Sonopuls GM2070 from Bandelin (Procedure B). IMAC was performed with column material from Macherey-Nagel GmbH & Co. KG containing Ni ions, such as Ni-NTA. Buffer exchange was performed using a column from GE Healthcare AB (V= 8.3 mL). A Merck KGaA filter unit with an exclusion limit of 30000 Da was used to constrict protein solutions. The extinction coefficient for protein concentration measurement was determined using ProtParam (ExPASy). UV/Vis spectroscopy was performed at 280 nm using a DeNovix spectrophotometer (type: DS-11+). GC-MS analyzes for in vitro biotransformations were performed using an Agilent 5977B GC/MSD with 7890B GC system and an Optima 5HT - 0.25 µm, carrier gas: He, column volume: 30 m x 250 µm x 0.25 µm, injection volume 1 µL. Retention indices (RI) were determined in comparison to *n*-alkanes (C7 to C30).

Composition of buffer solutions und culture media

In order to set pH value for the buffer solutions, aqueous NaOH and HCl solutions were used.

Lysis buffer: Tris·HCl (40 mM), NaCl (100 mM)

Ni-NTA buffer (x M): Tris·HCl (40 mM), NaCl (100 mM), imidazole (x M)

HEPES buffer: HEPES (50 mM), DTT (5 mM), pH =7.5

LB media:	0.50% (w/v) yeast extract (Duchefa Biochemie)
	1.00% (w/v) trypton (Duchefa Biochemie)
	0.50% (w/v) NaCl (Roth or VWR)
2-TY media:	1.00% (w/v) yeast extract (Duchefa Biochemie)
	1.60% (w/v) trypton (Duchefa Biochemie)
	0.50% (w/v) NaCl (Roth or VWR)
Binding buffer	Na_2HPO_4 (20 mM), NaCl (0.5 M), imidazole (20 mM), $MgCl_2$ (1 mM), pH = 7.4
Elution buffer	Na_2HPO_4 (20 mM), NaCl (0.5 M), imidazole (500 mM), $MgCl_2$ (1 mM), pH = 7.4
Incubation Buffer	Tris·HCl (50 mM), glycerine (10%), (2-Hydroxypropyl)-*β*-cyclodextrin (20 mM), $MgCl_2$ (10 mM), pH = 8.2

Geranylgeranyl pyrophosphate synthase (GGPPS)

Streptomyces cyanefuscatus WP_030565322.1

Amino acids sequence:

MGSSHHHHHHSSGLVPRGSHMASMTGGMARIPAVEHSFAACPLPPSPDPSATASTAA
GAVDADVVGAVLRTARAVLAERVAQASEIDASFAGELAGRVADFTLDGGKRMRPR
LLWWGMRACGAVDVGSTAAALRLGVALELIQTCALIHDDVMDRSRLRRGKPAVHI
GLAARAGLSPDSERGSAFGTSAAVLAGDLALAWADDTVAETALPAPVRRRIGAIWRS
LRTEMVAGQYLDLHGQATGGSSAARAIRTACLKSALYSVERPLAIGAALADADEHTT
AALRSAGRCAGIAFQLRDDLLGVFGDPARTGKPSGDDIREGKPTYLLAVARERAEAA
GDEGALAVLGHAVGNADVTEGDLADVRGVFEATGARAHVERKAERLRDHAVRRL
GEAVDVDAHGGRQLLG
LLRTVSGDPSGFGPATPDDPGGHHSPAAAGVLSAAEGGHVR

Spata-13,17-diene synthase (SpS)

Streptomyces xinghaiensis WP_095757924

Amino acids sequence:

MGSSHHHHHHSSGLVPRGSHMASMTGGVDAVHGPEDGSGPGSESGSASRPGAAQPP
EIHCPFPSEMSPHAEHAEAHLDAWVARFEVVRGTVARERFGRAGFAQFAARTYPTA
DRACLDLVADWFGWLFLVDDQLDDGRVGRDIDSARRAMDGLLRVLDREGPAEGER
PPGEPPLAWALRDLWHRTASRATPAWRRRFTGHLAACLEAACWEAENRIAGVVPGE
AEYIEQRRHTGAIYVCMDLIDIVGDLDLPEAVHAGEPFQAVLRASSDVVVWTNDWY

SLGKEMALGEYHNLVRVVAHARRLTLREALEHTAAAISAETRRYLGHRERLLAAHP EHRAA LTTCLAGMESWMRGNLDWSRATLRYRERERGGLPAYLEATLAPAGTEGGT

1.2 Chemical syntheses

***tert*-Butyl((3-methylbut-2-en-1-yl)oxy)diphenylsilane (S1)**[S1]

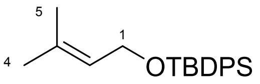

S1
$C_{21}H_{28}OSi$
324.54

Prenol (2.33 mL, 2.00 g, 23.3 mmol, 1.00 eq.) was dissolved in CH_2Cl_2 (20 mL), imidazole (3.98 g, 58.5 mmol, 2.51 eq.) and TBDPSCl (6.2 mL, 6.55 g, 23.8 mmol, 1.03 eq.) were added and the reaction mixture stirred at rt o/n. An aq. sat. NH_4Cl solution and Et_2O were added and the phases were separated. The aqueous phase was extracted with Et_2O (3x), the combined organic phases were dried over $MgSO_4{\cdot}H_2O$ and filtered. The solvent was removed *in vacuo* and the crude product was purified by column chromatography (PE : EtOAc= 50:1 → 10:1) to yield silylether **S1** (6.81 g, 21.0 mmol; 90%) as a colorless oil.

R_f = 0.19 (*n*-pentane 100%); ^{1}H NMR (400 MHz, $CDCl_3$): δ = 7.71 – 7.68 (m, 4H, H_{Ar}), 7.44 – 7.35 (m, 6H, H_{Ar}), 5.40 – 5.35 (m, 1H, H_2), 4.20 (d, J = 6.4 Hz, 2H, H_1), 1.69 (m, 3H, $H_{4/5}$), 1.46 (s, 3H, $H_{4/5}$), 1.04 (s, 9H, $H_{tert\text{-Bu}}$) ppm; ^{13}C NMR (101 MHz, $CDCl_3$): δ = 135.8 (C_{Ar}), 134.2 (C_{Ar}), 133.9 (C_3), 129.6 (C_{Ar}), 127.7 (C_{Ar}), 124.3 (C_2), 61.3 (C_1), 27.0 ($C_{tert\text{-Bu}}$), 25.9 ($C_{4/5}$), 19.3 ($C_{tert\text{-Bu}}$), 18.1 ($C_{4/5}$) ppm; GC-MS [EI, 12.371 min] *m/z* (%): 199.1 (100), 267.1 (28).

(*E*)-4-((*tert*-Butyldiphenylsilyl)oxy)-2-methylbut-2-en-1-ol (S2) [S1]

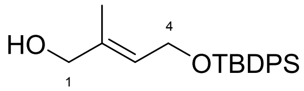

S2
$C_{21}H_{28}O_2Si$
340.54

t-BuOOH (70% in H_2O, 54 mL, 35.5 g, 394 mmol, 3.47 eq.) was given to a mixture of selenium dioxide (1.27 g, 11.4 mmol, 0.10 eq.) and salicylic acid (1.57 g, 11.4 mmol, 0.10 eq.) in CH_2Cl_2 (100 mL) and stirred for 5 min at rt. Silyl ether **S1** (36.9 g, 114 mmol, 1.00 eq.) was dissolved in CH_2Cl_2 (10 mL + 25 mL) and added to the reaction. After stirring at rt for about 44 h brine, a sat. aq. $NaHCO_3$ solution and EtOAc were added. The phases were separated and the aqueous phase was extracted with EtOAc (3x). The combined organic phases were washed with brine, dried over $MgSO_4{\cdot}H_2O$, filtered and the solvent was removed *in vacuo*. The residue was dissolved in MeOH (200 mL) and cooled to 0 °C. $NaBH_4$ (3.61 g, 95.4 mmol, 0.84 eq.) was slowly added over a period of about 40 min. After stirring for 0.5 h at 0 °C, the reaction mixture was warmed to rt and stirred for further 20 min. Brine, a sat. aq. $NaHCO_3$ solution and EtOAc were added and the phases were separated. The aqueous phase was extracted with EtOAc (3x)

and the combined organic phases were washed with brine and dried over $MgSO_4 \cdot H_2O$. After filtration, the solvent was removed *in vacuo* and the crude product was purified twice by column chromatography (PE:EtOAc= 4:1) to yield alcohol **S2** (22.9 g, 67.3 mmol; 59%) as a pale yellow oil.

R_f = 0.32 (*n*-pentane:Et_2O= 2:1); ^1H NMR (400 MHz, $CDCl_3$): δ = 7.70 – 7.68 (m, 4H, H_{Ar}), 7.45 – 7.36 (m, 6H, H_{Ar}), 5.62 (tq, J = 9.3 Hz, 1.4 Hz, 1H, H_3), 4.28 (m, 2H, H_4), 3.96 (d, J = 6.2 Hz, H_1), 1.48 (s, 3H, H_5), 1.20 (t, J = 6.3 Hz, 1H, H_{OH}), 1.05 (s, 9H, $H_{tert\text{-}Bu}$) ppm; ^{13}C NMR (101 MHz, $CDCl_3$): δ = 136.3 (C_2), 135.8 (C_{Ar}), 134.0 (C_{Ar}), 129.7 (C_{Ar}), 127.8 (C_{Ar}), 125.1 (C_3), 68.4 (C_1), 60.9 (C_4), 27.0 ($C_{tert\text{-}Bu}$), 19.3 ($C_{tert\text{-}Bu}$), 13.9 (C_5) ppm; HRMS (ESI) m/z: $[M + Na]^+$ Calcd for $C_{21}H_{28}O_2SiNa$ 363.1756; Found: 363.1753.

(*E*)-((4-Bromo-3-methylbut-2-en-1-yl)oxy)(tert-butyl)diphenylsilane (14)[S1]

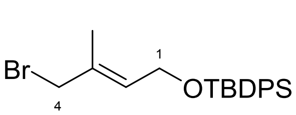

14
$C_{21}H_{27}BrOSi$
403.44

Alcohol **S2** (0.99 g, 2.91 mmol, 1.00 eq.) was dissolved in CH_2Cl_2 (15 mL) and cooled to 0 °C. PPh_3 (0.97 g, 3.68 mmol, 1.27 eq.) and NBS (0.65 g, 3.65 mmol, 1.26 eq.) were added to the solution and the reaction was stirred for 65 min at 0 °C. After dilution with Et_2O, the solvent was removed *in vacuo* and the residue was loaded onto silica with CH_2Cl_2. After column chromatography (PE 100% → PE / EtOAc= 100:1 → 50:1 → 10:1) bromide **14** (1.12 g, 2.78 mmol; 96%) was obtained as a pale yellow oil.

R_f = 0.11 (*n*-pentane 100%); ^1H-NMR (400 MHz, $CDCl_3$): δ = 7.68 – 7.66 (m, 4H, H_{Ar}), 7.45 – 7.37 (m, 6H, H_{Ar}), 5.79 (t, J = 6.0 Hz, 1H, H_2), 4.23 (d, J = 6.0 Hz, 2H, H_1), 3.93 (s, 2H, H_4), 1.58 – 1.57 (m, 3H, H_5), 1.04 (s, 9H, $H_{tert\text{-}Bu}$) ppm; ^{13}C NMR (101 MHz, $CDCl_3$): δ = 135.7 (C_{Ar}), 133.7 (C_{Ar}), 133.0 (C_3), 130.4 (C_2), 129.8 (C_{Ar}), 127.8 (C_{Ar}), 61.2 (C_1), 40.8 (C_4), 26.9 ($C_{tert\text{-}Bu}$), 19.3 ($C_{tert\text{-}Bu}$), 15.1 (C_5) ppm; GC-HRMS (CI) m/z: $[M+H]^+$ Calcd for $C_{21}H_{28}OSiBr$ 403.1093; Found: 403.1081.

Dimethyl (*E*)-hex-3-enedioate (10) [S3]

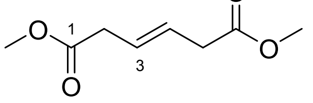

10
$C_8H_{12}O_4$
172.18

To a solution of (*E*)-hex-3-enedioic acid (25.0 g, 173 mmol, 1.00 eq.) in MeOH (125 mL) was added conc. H_2SO_4 (4.3 mL, 7.71 g, 78.6 mmol, 0.45 eq.). After stirring under refluxing

conditions overnight the reaction mixture was concentrated *in vacuo*. The residue was taken up in Et_2O and brine and the phases were separated. The aqueous phase was extracted with Et_2O (3x) and the combined organic phases were washed with a sat. aq. $NaHCO_3$ solution and brine. After drying over $MgSO_4{\cdot}H_2O$ the suspension was filtered and the solvent was removed *in vacuo*. Diester **10** (26.8 g, 156 mmol; 90%) was obtained as a colorless oil.

R_f = 0.31 (PE:EtOAc= 5:1); ^{1}H-NMR (400 MHz, $CDCl_3$): δ = 5.70 – 5.68 (m, 2H, H_3), 3.68 (s, 6H, H_{OMe}), 3.10 – 3.09 (m, 4H, H_2) ppm; ^{13}C NMR (101 MHz, $CDCl_3$): δ = 172.1 (C_1), 126.1 (C_3), 52.0 (C_{OMe}), 37.8 (C_2) ppm; HRMS (ESI) m/z: $[M+Na]^+$ Calcd for $C_8H_{12}O_4Na$ 195.0633; Found: 195.0632.

(*E*)-Hex-3-ene-1,6-diol (S3)[S3]

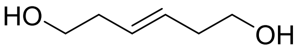

S3
$C_6H_{12}O_2$
116.16

Diester **10** (1.01 g, 5.84 mmol, 1.0 eq.) was dissolved in THF (18 mL) and cooled to −78 °C. DIBAL-H (1 M in hexane, 30 mL, 30 mmol, 5.13 eq.) was added and after 80 min the reaction mixture was carefully poured into an aqueous solution of Rochelle salt at 0 °C. Stirring was continued at rt o/n to separate the phases. The aqueous phase was extracted with a mixture of CH_2Cl_2:*i*PrOH (3:1, 14x) and the combined organic phases were dried over $MgSO_4{\cdot}H_2O$, filtered and the solvent was removed *in vacuo*. Diol **S3** (650 mg, 5.60 mmol; 96%) was obtained as a colorless oil.

R_f = 0.22 (PE:EtOAc= 1:4); ^{1}H-NMR (400 MHz, $CDCl_3$): δ = 5.54 – 5.52 (m, 2H, H_3), 3.65 (t, J = 6.2 Hz, 2H, H_1), 2.32 – 2.27 (m, 4H, H_2), 1.76 (s_{broad}, 2H, H_{OH}) ppm; ^{13}C NMR (101 MHz, $CDCl_3$): δ = 129.8 (C_3), 61.9 (C_1), 36.1 (C_2) ppm; HRMS (MS) m/z: $[M+Na]^+$ Calcd for $C_6H_{12}O_2Na$ 139.0735; Found: 139.0732.

(*E*)-6-((*tert*-Butyldiphenylsilyl)oxy)hex-3-en-1-ol (11)[S4]

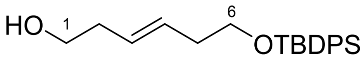

11
$C_{22}H_{30}O_2Si$
354.57

Diol **S3** (6.08 g, 52.3 mmol, 2.00 eq.) was dissolved in *N*,*N*-DMF (50 mL). Imidazole (1.75 g, 25.7 mmol, 0.98 eq.) and TBDPSCl (6.81 mL, 7.20 g, 26.2 mmol, 1.00 eq.) were added and the reaction mixture was stirred at rt o/n. A sat. aq. NH_4Cl solution was added and after extraction with Et_2O (4x) and EtOAc (5x) the combined organic phases were dried over $MgSO_4{\cdot}H_2O$, filtered and the solvent was removed *in vacuo*. The crude product was purified by column

chromatography (PE:EtOAc= 4:1 → 2:1 → EtOAc 100% to reisolate diol **S4**) and alcohol **11** (6.21 g, 17.5 mmol; 67%) was obtained as a colorless oil.

R_f = 0.33 (PE:EtOAc= 4:1); ^{1}H-NMR (400 MHz, $CDCl_3$): δ = 7.68 – 7.65 (m, 4H, H_{Ar}), 7.45 – 7.36 (m, 6H, H_{Ar}), 5.60 – 5.53 (m, 1H, H_4), 5.47 – 5.40 (m, 1H, H_3), 3.69 (t, J = 6.6 Hz, 2H, H_6), 3.62 (dt, J = 6.0 Hz, 5.8 Hz, 2H, H_1), 2.31 – 2.24 (m, 4H, H_2, H_5), 1.37 (t, J = 5.5 Hz, 1H, H_{OH}), 1.05 (s, 9H, $H_{tert\text{-}Bu}$) ppm; ^{13}C NMR (101 MHz, $CDCl_3$): δ = 135.7 (C_{Ar}), 134.1 (C_{Ar}), 130.7 (C_4), 129.7 (C_{Ar}), 128.2 (C_3), 127.8 (C_{Ar}), 63.8 (C_6), 62.0 (C_1), 36.2 (C_2, C_5), 27.0 ($C_{tert\text{-}Bu}$), 19.4 ($C_{tert\text{-}Bu}$) ppm; HRMS (ESI) m/z: $[M+Na]^+$ Calcd for $C_{22}H_{30}O_2NaSi$ 377.1913; Found: 377.1917.

(*E*)-6-((*tert*-Butyldiphenylsilyl)oxy)hex-3-enal (S4)[S4, S5]

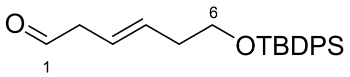

S4
$C_{22}H_{28}O_2Si$
352.55

Alcohol **11** (12.0 g, 33.8 mmol, 1.00 eq.) was dissolved in THF (200 mL) and cooled to 0 °C. Dess-Martin-periodinane (20.1 g, 47.5 mmol, 1.41 eq.) was added stepwise in three portions and after 2 h the reaction mixture was warmed to rt. After 2.25 h *n*-pentane and water were added and the white solid was filtered off and washed with *n*-pentane (4x). The combined organic phases were dried over $MgSO_4 \cdot H_2O$, filtered and the solvent was removed *in vacuo*. The crude product, which appears as a yellow oil, was directly used for the next reaction without further purification. The yield will be calculated after three steps.

R_f = 0.53 (PE:EtOAc= 6:1); ^{1}H-NMR (400 MHz, $CDCl_3$): δ = 9.64 (t, J = 2.1 Hz, 1H, H_1), 7.67 – 7.64 (m, 4H, H_{Ar}), 7.45 – 7.36 (m, 6H, H_{Ar}), 5.67 – 5.51 (m, 2H, H_3, H_4), 3.71 (t, J = 6.5 Hz, 2H, H_6), 3.11 – 3.10 (m, 2H, H_2), 2.32 (dt, J = 6.4 Hz, 6.4 Hz, 2H, H_5), 1.04 (s, 9H, H_{tertBu}) ppm; ^{13}C NMR (101 MHz, $CDCl_3$): δ = 200.4 (C_1), 135.7 (C_{Ar}), 134.0 (C_{Ar}), 133.4 (C_3), 129.8 (C_{Ar}), 127.8 (C_{Ar}), 121.4 (C_4), 63.6 (C_6), 47.5 (C_2), 36.2 (C_5), 27.0 (C_{tertBu}), 19.4 (C_{tertBu}) ppm; HRMS (ESI) m/z: $[M+Na]^+$ Calcd for $C_{22}H_{28}O_2NaSi$ 375.1756; Found: 375.1746

(*E*)-*tert*-Butyl((7-methylocta-3,6-dien-1-yl)oxy)diphenylsilane (S5)

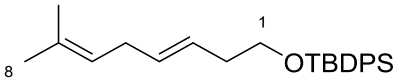

S5
$C_{25}H_{34}OSi$
378.63

i-$PrPPh_3I$ (24.9 g, 57.5 mmol, 1.70 eq.) in THF (120 mL) was cooled to 0 °C and *n*-BuLi (1.6 M in hexane, 35 mL, 56.0 mmol, 1.66 eq.) was added. After 100 min the reaction was warmed to rt. Aldehyde **S4** (11.9 g, 33.8 mmol, 1.00 eq.) was dissolved THF (15 mL + 35 mL) and added after additional 75 min. The reaction mixture was stirred at 40 °C o/n before adding brine. After

extraction with CH_2Cl_2 (4x) the combined organic phases were washed with H_2O (2x) and dried over $MgSO_4 \cdot H_2O$. After filtration the solvent was removed *in vacuo* and the crude product was purified by column chromatography (PE:EtOAc= 50:1). Alkene **S5** was obtained as a pale-yellow oil.

R_f = 0.53 (PE:EtOAc= 50:1); ^{1}H-NMR (400 MHz, $CDCl_3$): δ = 7.68 – 7.66 (m, 4H, H_{Ar}), 7.44 – 7.29 (m, 6H, H_{Ar}), 5.44 – 5.41 (m, 2H, H_3, H_4), 5.14 – 5.10 (m, 1H, H_6), 3.67 (t, J = 6.7 Hz, 2H, H_1), 2.69 – 2.64 (m, 2H, H_5), 2.28 – 2.24 (m, 2H, H_2), 1.70 (m, 3H, $H_{8/9}$), 1.61 (s, 3H, $H_{8/9}$), 1.05 (s, 9H, $H_{tert\text{-}Bu}$) ppm; ^{13}C NMR (101 MHz, $CDCl_3$): δ = 135.8 (C_{Ar}), 134.2 (C_{Ar}), 132.3 (C_7), 131.1 (C_4), 129.6 (C_{Ar}), 127.7 (C_{Ar}), 126.6 (C_3), 122.6 (C_6), 64.1 (C_1), 36.1 (C_2), 31.5 (C_5), 27.0 ($C_{tert\text{-}Bu}$), 25.9 ($C_{8/9}$), 19.4 ($C_{tert\text{-}Bu}$), 17.8 ($C_{8/9}$) ppm; HRMS (ESI) m/z: $[M+Na]^+$ Calcd for $C_{25}H_{34}ONaSi$ 401.2277; Found: 401.2267.

(*E*)-7-Methylocta-3,6-dien-1-ol (12)

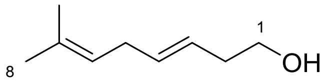

12
$C_9H_{16}O$
140.23

Alkene **S5** (8.31 g, 21.9 mmol, 1.00 eq.) was dissolved in THF (120 mL) and cooled to 0 °C. TBAF (1 M in THF, 28 mL, 28.0 mmol, 1.28 eq.) was added and the reaction mixture was stirred o/n while slowly warming up to rt. Water and EtOAc were added and the phases were separated. The aqueous phase was extracted with EtOAc (3x) and the combined organic phases were washed with brine, dried over $MgSO_4 \cdot H_2O$, filtered and the solvent was removed *in vacuo*. The crude product was purified by column chromatography (PE:EtOAc= 6:1 → 4.7:1) and alcohol **12** (2.36 g, 16.8 mmol; 50% o.3.s.) was obtained as a pale-yellow oil.

R_f = 0.42 (PE:EtOAc= 3:1); ^{1}H-NMR (400 MHz, $CDCl_3$): δ = 5.54 (dtt, J = 15.3 Hz, 6.3 Hz, 1.2 Hz, 1H, H_4), 5.39 (dtt, J = 15.3 Hz, 6.9 Hz, 1.4 Hz, 1H, H_3), 5.16 – 5.11 (m, 1H, H_6), 3.63 (t, J = 6.3 Hz, 2H, H_1), 2.71 (t, J = 6.7 Hz, 2H, H_5), 2.30 – 2.24 (m, 2H, H_2), 1.71 (m, 3H, $H_{8/9}$), 1.61 (s, 3H, $H_{8/9}$), 1.40 (s_{broad}, 1H, H_{OH}) ppm; ^{13}C NMR (101 MHz, $CDCl_3$): δ = 132.8 ($C_{4/7}$), 132.7 ($C_{4/7}$), 125.9 (C_3), 122.2 (C_6), 62.2 (C_1), 36.1 (C_2), 31.5 (C_5), 25.8 ($C_{8/9}$), 17.8 ($C_{8/9}$) ppm; GC-HRMS (CI; 6.98 min) m/z: $[M]^+$ Calcd for $C_9H_{16}O$ 140.1201; Found: 140.1201.

(*E*)-8-Bromo-2-methylocta-2,5-diene (S6)

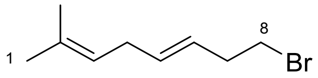

S6
$C_9H_{15}Br$
203.12

CBr_4 (6.72 g, 20.3 mmol, 1.20 eq.) was dissolved in CH_2Cl_2 (20 mL) at rt. Alcohol **12** (2.36 g, 16.8 mmol, 1.00 eq.) was dissolved in CH_2Cl_2 (6 mL + 18 mL) and added to the solution. At

0 °C PPh_3 (4.89 g, 18.6 mmol, 1.11 eq.) was added and after 17 min the reaction was warmed to rt. After stirring additional 65 min at rt, the solvent was removed *in vacuo* and the crude product was purified by column chromatography (*n*-pentane:Et_2O= 100:1). A portion of the material was washed with water and the aqueous phase was extracted with *n*-pentane (2x). Finally, bromide **S6** (3.46 g, 17.0 mmol, quant.) was obtained as a colorless oil.

R_f = 0.84 (PE:EtOAc= 20:1); ^{1}H-NMR (400 MHz, $CDCl_3$): δ = 5.52 (dtt, J = 15.3 Hz, 6.3 Hz, 1.2 Hz, 1H, H_5), 5.39 (dtt, J = 15.3 Hz, 6.6 Hz, 1.4 Hz, 1H, H_6), 5.15 – 5.10 (m, 1H, H_3), 3.36 (t, J = 7.2 Hz, 2H, H_8), 2.70 (t, J = 6.8 Hz, 2H, H_4), 2.58 – 2.52 (m, 2H, H_7), 1.71 (m, 3H, $H_{1/9}$), 1.61 (s, 3H, $H_{1/9}$) ppm; ^{13}C NMR (101 MHz, $CDCl_3$): δ = 132.8 (C_2), 132.5 (C_5), 126.5 (C_6), 122.0 (C_3), 36.2 (C_7), 33.0 (C_8), 31.4 (C_4), 25.8 ($C_{1/9}$), 17.8 ($C_{1/9}$) ppm; GC-HRMS (CI; 7.641 min) m/z: $[M]^+$ Calcd for $C_9H_{15}Br$ 202.0357; Found: 202.0361.

(*E*)-((7-Methylocta-3,6-dien-1-yl)sulfonyl)benzene (13)

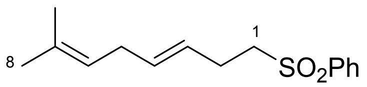

13
$C_{15}H_{20}O_2S$
264.38

Benzenesulfinic acid sodium salt (4.21 g, 25.6 mmol, 1.51 eq.) was dissolved in *N*,*N*-DMF (15 mL) and bromide **S6** (3.45 g, 17.0 mmol, 1.00 eq.) dissolved in *N*,*N*-DMF (10 mL + 20 mL) was added at rt. The reaction stirred for 4 h at 50 °C and then o/n at rt. Brine and EtOAc were added and the phases were separated. The aqueous phase was extracted with EtOAc (3x) and the combined organic phases were washed with brine, dried over $MgSO_4 \cdot H_2O$, filtered and the solvent was removed *in vacuo*. The crude product was purified by column chromatography (PE:EtOAc= 6:1) and sulfone **13** (2.69 g, 10.2 mmol; 60%) was obtained as a colorless oil.

R_f = 0.29 (PE:EtOAc= 6:1); ^{1}H-NMR (400 MHz, $CDCl_3$): δ = 7.92 – 7.89 (m, 2H, H_{Ar}), 7.68 – 7.63 (m, 1H, H_{Ar}), 7.59 – 7.55 (m, 2H, H_{Ar}), 5.46 – 5.39 (m, 1H, H_4), 5.32 – 5.24 (m, 1H, H_3), 5.06 – 5.01 (m, 1H, H_6), 3.15 – 3.11 (m, 2H, H_1), 2.62 (t, J = 6.6 Hz, 2H, H_5), 2.43 – 2.37 (m, 2H, H_2), 1.68 (m, 3H, $H_{8/9}$), 1.57 (s, 3H, $H_{8/9}$) ppm; ^{13}C NMR (101 MHz, $CDCl_3$): δ = 139.3 (C_{Ar}), 133.8 (C_{Ar}), 133.0 (C_7), 132.1 (C_4), 129.4 (C_{Ar}), 128.3 (C_{Ar}), 125.0 (C_3), 121.6 (C_6), 56.1 (C_1), 31.2 (C_5), 26.0 (C_2), 25.8 (C_8/C_9), 17.7 ($C_{8/9}$) ppm; HRMS (ESI) m/z: $[M+Na]^+$ Calcd for $C_{15}H_{20}O_2NaS$ 287.1082; Found: 287.1069.

Sulfone (15)

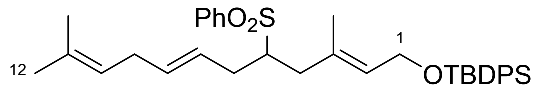

15
$C_{36}H_{46}O_3SSi$
586.91

Sulfone **13** (2.68 g, 10.1 mmol, 1.00 eq.) was dissolved in THF (70 mL) and cooled to −78 °C. *n*-BuLi (1.6 M in hexane, 6.60 mL, 10.6 mmol, 1.05 eq.) was slowly added to the solution and the reaction stirred for 2 h at −78 °C. Bromide **14** (4.18 g, 10.4 mmol, 1.03 eq.) was dissolved in THF (20 mL + 90 mL) and added to the reaction mixture, that kept stirring at −78 °C for 2.5 h before warming up to rt. Stirring was continued for further 3.5 h. Then, water was added and after separating the phases, the aqueous phase was extracted with EtOAc (3x). The combined organic phases were washed with a sat. aq. NH_4Cl-solution, water and brine. After drying over $MgSO_4{\cdot}H_2O$ and filtration the solvent was removed *in vacuo*. The crude product was purified by column chromatography (*n*-pentane:Et_2O= 6:1 → 5:1 → 3:1) and sulfone **15** (4.78 g, 8.14 mmol; 80%) was obtained as a highly viscous yellow oil.

R_f = 0.54 (PE:EtOAc, 3:1); ^{1}H-NMR (400 MHz, $CDCl_3$): δ = 7.88 – 7.86 (m, 2H, H_{SO2Ph}), 7.68 – 7.59 (m, 5H, $H_{SO2Ph,}$ H_{OTBDPS}), 7.57 – 7.51 (m, 2H, H_{SO2Ph}), 7.45 – 7.34 (m, 6H, H_{OTBDPS}), 5.39 – 5.24 (m, 3H, H_2, H_7, H_8), 5.04 – 4.98 (m, 1H, H_{10}), 4.13 (d, J = 6.1 Hz, 2H, H_1), 3.14 – 3.06 (m, 1H, H_5), 2.64 – 2.46 (m, 4H, H_4, H_9), 2.31 – 2.18 (m, 2H, H_6), 1.67 – 1.66 (m, 3H, $H_{12/14}$), 1.56 (s, 3H, $H_{12/14}$), 1.31 (s, 3H, H_{13}), 1.03 (s, 9H, $H_{tert\text{-}Bu}$) ppm; ^{13}C NMR (101 MHz, $CDCl_3$): δ = 138.3 (C_{SO2Ph}), 135.7 (d, C_{OTBDPS}), 133.9 (d, C_{OTBDPS}), 133.7 (C_{SO2Ph}), 133.0 (C_8), 132.7 (C_{11}), 131.8 (C_3), 129.8 (d, C_{OTBDPS}), 129.2 (C_{SO2Ph}), 129.1 (C_{SO2Ph}), 127.8 (C_{OTBDPS}), 125.0 (C_7), 121.9 (C_{10}), 62.7 (C_5), 61.0 (C_1), 37.8 (C_4), 31.3 (C_9), 31.1 (C_6), 26.9 ($C_{tert\text{-}Bu}$), 25.8 ($C_{12/14}$), 19.3 ($C_{tert\text{-}Bu}$), 17.8 ($C_{12/14}$), 16.0 (C_{13}) ppm; HRMS (ESI) m/z: $[M+Na]^+$ Calcd for $C_{36}H_{46}O_3NaSSi$ 609.2835; Found: 609.2809.

Silylether S7

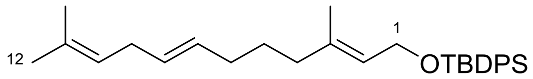

S7
$C_{30}H_{42}OSi$
446.75

To a flask containing Na_2HPO_4 (1.81 g, 12.8 mmol, 1.57 eq.) was added sulfone **15** (4.78 g, 8.14 mmol, 1.00 eq.) in THF (10 mL + 20 mL) and MeOH (60 mL) at −14 °C. NaHg (5% Na in Hg, 17.9 g) was added and after 15 min the reaction was warmed to rt and stirred o/n. The reaction was filtered through Celite® 535 (Et_2O). The organic phase was washed with water, the phases were separated and the aqueous phase was extracted with Et_2O (3x). The combined organic phases were washed with a sat. aq. NH_4Cl solution, a sat. aq. $NaHCO_3$ solution, water and brine. After drying with $MgSO_4{\cdot}H_2O$ and filtration the solvent was removed *in vacuo*. The crude product was purified by column chromatography (PE:EtOAc= 40:1 → 20:1). Silylether **S7** (3.19 g, 7.13 mmol; 88%) was obtained as a colorless oil. When progressing with the column chromatography (PE:EtOAc= 20:1 → 2:1) alcohol **S8** (119 mg, 0.57 mmol, 7%) was also collected as a yellow-orange oil.

R_f = 0.84 (PE:EtOAc, 20:1); ^{1}H-NMR (400 MHz, $CDCl_3$): δ = 7.71 – 7.68 (m, 4H, H_{Ar}), 7.44 – 7.35 (m, 6H, H_{Ar}), 5.41 – 5.34 (m, 3H, H_2, H_7, H_8), 5.17 – 5.12 (m, 1H, H_{10}), 4.22 (d, J = 6.3 Hz, 2H, H_1), 2.70 – 2.67 (m, 2H, H_9), 1.97 – 1.93 (m, 4H, H_4, H_6), 1.71 (m, 3H, H_{12}, H_{14}), 1.62 (s,

3H, H_{12}, H_{14}), 1.47 – 1.39 (m, 5H, H_5, H_{13}), 1.04 (s, 9H, $H_{tert\text{-}Bu}$) ppm; ^{13}C NMR (101 MHz, $CDCl_3$): δ = 137.3 (C_3), 135.8 (C_{Ar}), 134.3 (C_{Ar}), 132.2 (C_{11}), 130.2 (C_7), 129.6 (C_{Ar}), 129.2 (C_8), 127.7 (C_{Ar}), 124.2 (C_2), 122.8 (C_{10}), 61.3 (C_1), 39.1 (C_4), 32.2 (C_6), 31.5 (C_9), 27.7 (C_5), 27.0 ($C_{tert\text{-}Bu}$), 25.9 ($C_{12/14}$), 19.3 ($C_{tert\text{-}Bu}$), 17.8 ($C_{12/14}$), 16.4 (C_{13}) ppm; HRMS (ESI) m/z: $[M+Na]^+$ Calcd for $C_{30}H_{42}ONaSi$ 469.2903; Found: 469.2892.

(2*E*,7*E*)-3,11-Dimethyldodeca-2,7,10-trien-1-ol (S8)

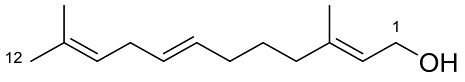

S8
$C_{14}H_{24}O$
208.35

Silylether **S7** (3.19 g, 7.13 mmol, 1.00 eq.) was dissolved in THF (70 mL) and cooled to 0 °C. TBAF (1 M in THF, 9 mL, 9.00 mmol, 1.26 eq.) was added and the reaction was warmed up to rt and stirring was continued o/n. Brine was added to the reaction mixture and the aqueous phase was extracted with EtOAc (3x). The combined organic phases were dried over $MgSO_4 \cdot H_2O$, filtered and the solvent was removed *in vacuo*. The crude product was purified by column chromatography (PE:EtOAc= 6:1 → 3:1) and alcohol **S8** (1.35 g, 6.48 mmol, 91%) was obtained as a colorless oil.

R_f = 0.47 (PE:EtOAc, 3:1); ^1H-NMR (400 MHz, $CDCl_3$): δ = 5.43 – 5.37 (m, 3H, H_2, H_7, H_8), 5.15 – 5.10 (m, 1H, H_{10}), 4.15 (dd, J = 5.8 Hz, 5.8 Hz, 2H, H_1), 2.69 – 2.66 (m, 2H, H_9), 2.03 – 1.95 (m, 4H, H_4, H_6), 1.71 – 1.70 (m, 3H, $H_{12/14}$), 1.66 (s, 3H, H_{13}), 1.61 (s, 3H, $H_{12/14}$), 1.51 – 1.44 (m, 2H, H_5), 1.09 (t, J = 5.3 Hz, 1H, H_{OH}) ppm; ^{13}C NMR (101 MHz, $CDCl_3$): δ = 140.1 (C_3), 132.3 (C_{11}), 130.0 (C_7), 129.3 (C_8), 123.5 (C_2), 122.7 (C_{10}), 59.6 (C_1), 39.2 (C_4), 32.3 (C_6), 31.5 (C_9), 27.7 (C_5), 25.9 ($C_{12/14}$), 17.8 ($C_{12/14}$), 16.3 (C_{13}) ppm; HRMS (ESI) m/z: $[M+Na]^+$ Calcd for $C_{14}H_{24}ONa$ 231.1725; Found: 231.1722.

FPP derivative 7

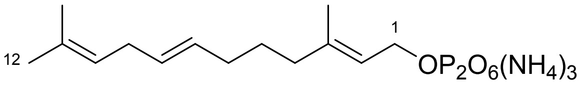

7
$C_{14}H_{35}N_3O_7P_2$
419.40

Alcohol **S8** (145 mg, 0.70 mmol, 1.00 eq.) was dissolved in THF (10 mL) and cooled to 0 °C. Et_3N (0.14 mL, 102 mg, 1.01 mmol, 1.45 eq.) followed by MsCl (0.10 mL, 148 mg, 1.29 mmol, 1.85 eq.) were added and the reaction mixture was stirred at 0 °C for 1 h. LiCl (137 mg, 3.23 mmol, 4.64 eq.) was added at 0°C and the reaction mixture was warmed up to rt over a period of 45 minutes. *n*-Pentane and water were added after 70 minutes and the phases were separated. The aqueous phase was extracted with *n*-pentane (3x) and the combined organic

Hier müsste das entsprechende Malzeichen hin, wie auch zuvor bei den Vorschriften

phases were dried over $MgSO_4$□H_2O. After filtration, the solvent was removed *in vacuo* and the crude chloride was used directly in the next step.

Tris(tetra-*n*-butylammonium) hydrogen pyrophosphate (1.33 g, 1.47 mmol, 2.11 eq,) was dissolved in MeCN (5 mL) at rt. The crude allyl chloride (from above) was dissolved in MeCN (2 mL + 3 mL) and added at rt. The reaction mixture was stirred o/n before the solvent was removed *in vacuo*. The residue was subjected to purification using an ion exchange resin (see general information) resulting in FPP-derivative **7** (265 mg, 0.63 mmol, 91% o.2.s) as a fluffy yellowish solid showing a slight impurity (likely $MeSO_3H$). In order to remove the impurity, the product was purified by column chromatography (*i*PrOH:NH_3 (conc.):H_2O= 6:3:1) leading to a colorless fluffy solid (96 mg, 0.23 mmol, 33% o.2.s). The solvent signal for D_2O was supressed in the NMR spectrum to enhance the product signal visibility.

^{1}H NMR (400 MHz, D_2O): δ = 5.49 – 5.35 (m, 3H, H_7, H_8, H_{10}), 5.18 – 5.14 (m, 1H, H_2), 4.39 (dd, J = 6.6 Hz, 6.6 Hz, 2H, H_1), 2.62 (dd, J = 6.5 Hz, 6.0 Hz, H_9), 1.99 – 1.88 (m, 4H, H_4, H_6), 1.62 (s, 6H, H_{12}/H_{14}, H_{13}), 1.54 (s, 3H, H_{12}/H_{14}), 1.45 – 1.37 (m, 2H, H_5) ppm; ^{13}C NMR (101 MHz, D_2O): δ = 143.2 (C_3), 134.1 (C_{11}), 130.9 (C_7/C_8), 129.1 (C_7/C_8), 122.4 (C_{10}), 119.7 (d, C_2), 62.5 (d, C_1), 38.3 (C_4), 31.4 (C_6), 30.6 (C_9), 26.8 (C_5), 24.8 (C_{12}/C_{14}), 16.8 (C_{12}/C_{14}), 15.5 (C_{13}) ppm; ^{31}P NMR (162 MHZ, D_2O): δ = −6.62 (d, $J_{P,P}$ = 22.0 Hz), −10.0 (d, $J_{P,P}$ = 21.9 Hz) ppm. HRMS [ESI-MS]: *m/z* calcd for $C_{14}H_{25}O_7P_2$ [M-$(NH_4)_3$+H_2]$^-$: 367.1076, found: 367.1074.

Ammonium salt of IPP (2)[S6]

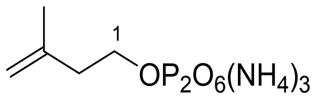

2

$C_5H_{21}N_3O_7P_2$

297.18

Isoprenol (0.3 mL, 256 mg, 2.97 mmol, 1.00 eq.) was dissolved in CH_2Cl_2 (10 mL) and cooled to 0 °C. DMAP (1.23 g, 10.0 mmol, 3.38 eq.) and TsCl (1.42 g, 7.45 mmol, 2.51 eq.) were added and the reaction mixture was stirred at rt for 3 h. After adding a sat. aq. NH_4Cl solution, the phases were separated and the aqueous phase was extracted with EtOAc (3x). The combined organic phases were dried over $MgSO_4\cdot H_2O$, filtered and the solvent was removed *in vacuo*. The crude product was purified by column chromatography (PE:EtOAc= 4:1) and the corresponding tosylate (614 mg, 2.55 mmol; 86%) was obtained, which was then dissolved in MeCN (5 mL) and added to a solution of tris(tetra-*n*-butylammonium)hydrogen pyrophosphate (3.47 g, 3.85 mmol, 1.29 eq.) in MeCN (20 mL). The reaction mixture stirred at rt o/n before the solvent was removed *in vacuo*. The residue was purified by ion exchange chromatography (see general information) resulting in the ammonium salt of IPP (**2**) (1.33 g, 4.47 mmol; quant.; still containing impurities resulting from tosylate) as a semisolid white material.

^{1}H NMR (400 MHz, D_2O): δ = 4.88 – 4.81 (m, 2H, H_4), 4.06 (dt, J = 6.7 Hz, 6.7 Hz, 2H, H_1), 2.40 (t, J = 6.7 Hz, 2H, H_2), 1.78 (s, 3H, H_5) ppm; ^{13}C NMR (151 MHz, D_2O): δ = 142.5 (C_3), 111.5 (C_4), 64.3 (d, J = 5.5 Hz, C_1), 37.9 (d, J = 7.5 Hz, C_2), 21.6 (C_5), ppm.

1.3 Microbiological methods and biotransformations

1.3.1 Procedure A

Heterologous protein expression and cell lysis *via* ultrasound

In order to cultivate the *E.coli* BL21 (DE3) cells, carrying the required plasmids, a seed culture (50 µL) was incubated with kanamycin (50 mg/mL, 3 µL) in LB-media (3 mL) for 4.5 h at 37 °C and 200 rpm. Alternatively, a seed culture (5 µL) can be incubated with kanamycin (50 mg/mL, 5 µL) in LB-Media (5 mL) at 37 °C and 180 rpm o/n. From this pre-culture (1 mL) a main culture was created by incubation with kanamycin (50 mg/mL) in 2-TY media (50 mL) at 37 °C and 200 rpm until the culture reached an OD_{600} value of a 0.4 to 0.8. To initiate the protein overexpression IPTG (1 M, 25 µL or 50 µL) was added to the culture that was stirred at 16 °C and 180 rpm for approximately 22 h. After centrifugation, the cell pellets were stored at −20 °C or used immediately for cell lysis. Cells were resuspended in lysis buffer (20 mL) at 0 °C and lyzed by ultrasonication (10 min, 45% amplitude, 4 s ultrasound to 6 s pause). The resulting solution was centrifuged (4 °C, 20 min, 10000 g) to give the crude enzyme solution.

Immobilized metal-affinity chromatography

For conditioning the column was rinsed with water (10x the column volume) and lysis buffer (5x column volume). The lysate was loaded onto the column (2x) and eluted with Ni-NTA buffers (5 mL each) with increasing imidazole concentrations (25 mM, 50 mM, 100 mM, 250 mM, 500 mM). During this time the solutions were cooled at 0 °C. The fractions were analyzed using a Brentford assay and those fractions containing protein were united and concentrated by centrifugation (20 min, 4 °C, 4500 rpm).

Buffer exchange

To perform the buffer exchange the column was rinsed with water (10x column volume) and HEPES buffer (5x column Volume). The protein solutions were loaded onto the column and eluted with HEPES buffer (5 mL). After centrifugation (4 °C, 4500 rpm) the solutions can be used or stored as a mixture of water and glycerol (1/1) between −70 °C and −80 °C.

Concentration measurement

Concentrations were determined by measuring the absorption (λ = 280 nm) of the purified protein solutions, using the extinction coefficient for reduced cysteine side chains.

***In-vitro* biotransformation (analytical scale)**

Screening for new biotransformation products was performed in a reaction scale of 500 µL containing the corresponding enzyme (50 µg), the FPP derivatives **6-8** (1.5 µL, 50 mM), IPP **2**

(excess) and a $MgCl_2$ solution (1.25 µL, 2 M). In parallel also negative (without FPP derivative or in the absence of enzymes) as well as positive control experiments were performed (using geranylgeranylpyrophosphate) under analogous conditions. All reactions were carried out in HEPES buffer (pH = 7.5) at 30 °C and 100 rpm o/n. In order to extract the products *n*-hexane was added and the phases were separated by centrifugation (3000 rpm, 6 min, 4 °C). The hexane extract was used for GC-MS analysis.

1.3.1 Procedure B

Heterologous protein expression and cell lysis *via* ultrasound

To grow 1 L of cell culture (*E. coli* BL21 (DE3)) in LB media with kanamycin (1 mL, 50 mg/mL), a culture was incubated at 37 °C and 150 rpm to OD_{600} = 0.4 to 0.6. The culture was then allowed to equilibrate at 20 °C for 30 min, before inducing overexpression by adding IPTG (final concentration: 0.4 mM). The culture was then incubated at 20 °C for 15 h during the overexpression. The cells were centrifugated (4 °C, 4000 rpm, 30 min) and the supernatant was discarded. The cells were then resuspended in ddH_2O (25 mL), centrifuged (4 °C, 5000 rpm, 10 min) and stored at −80 °C until further use. To extract the proteins, the cells (of 1.5 L cell culture) were resuspended in binding buffer (25 mL to 30 mL) and kept at 4 °C or below from here on. Cell lysis was performed by ultrasonication (47%, cycle 5·10%) followed by centrifugation (12 °C, 11000 rpm, 10 min).

Immobilized metal-affinity chromatography

For conditioning the column was rinsed with water (3 to 5 x the column volume) and cold binding buffer (at least 5 x column volume). The soluble protein fraction was filtered through a 0.22 µm or a 0.45 µm filter or a usual filter paper immediately before applying it to the column. The column was then rinsed again with binding buffer (10-15 x column volume, approx. 30 mL). The proteins were then eluted with cold elution buffer (approx. 20 mL). If desired the proteins can be concentrated by centrifugation (3000 g, 4 °C).

Concentration measurement

Preparation of 5x Bradford solution Coomassie Brilliant Blue G-250 (100 mg) was dissolved in ethanol (95%, 50 mL). To this solution was added H_3PO_4 (85% w/v, 100 mL) and the mixture was filled up to 200 mL with H_2O. The solution was diluted 5 times before usage.

The protein solution (3 µL) was added to 1x Brandford solution (1 mL) and the absorption was measured at λ = 595 nm. Blank control was made by using the elution buffer. The concentration was obtained based on the calibration curve made by mixing Bovine Serum Albumin solutions of defined concentrations and Bradford solution (Figure S1).

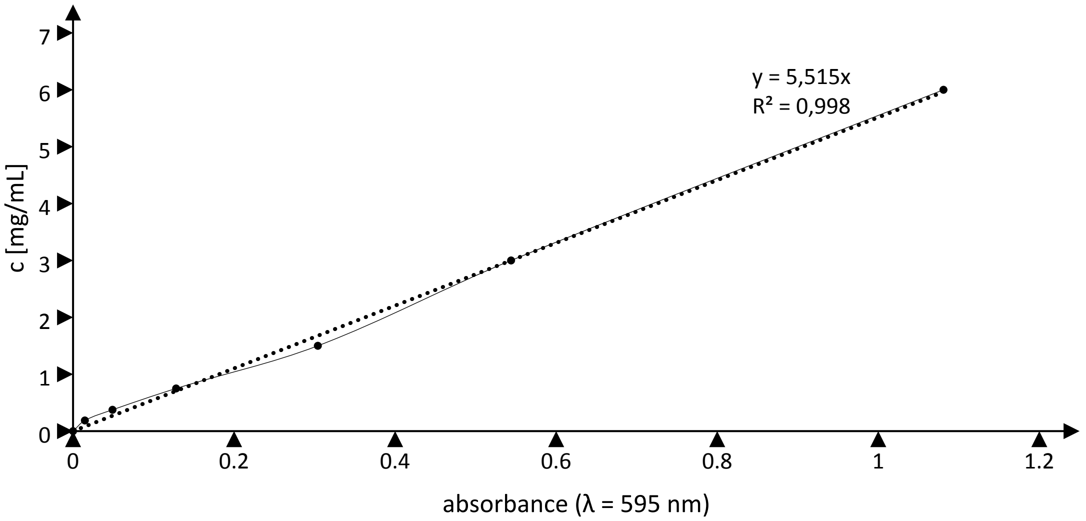

Figure S1. Calibration curve for the determination of protein concentrations.

Biotransformation for product isolation with FPP derivative 16

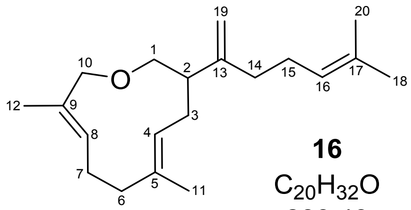

16
$C_{20}H_{32}O$
288.48

FPP derivative **6** (45 mg, 0.10 mmol) and IPP **2** (79 mg, 0.27 mmol) were dissolved in an aq. NH_4HCO_3 solution (0.05 M, 5.34 mL) and cooled at 0 °C. HEPES buffer (48.2 mL), Tween® 20 (3 µL) and PPase (100 U/mL, 2 µL) were mixed. Then a GGPPS solution (29.9 mg/mL, 168 µL), a SpS solution (4.16 mg/mL, 1.20 mL) as well as an aq. $MgCl_2$ solution (2 M, 625 µL) were added. The reaction was tempered at 30 °C and 180 rpm. Then, the FPP derivative **6**/ IPP **2** solutions (530 µL) were slowly added to the reaction mixture (3.47 mL, 1.06 mL/h). After 2 h continuous addition was stopped for a short period to add additional GGPPS (29.9 mg/mL, 168 µL) and SpS (4.16 mg/mL, 150 µL and 2.62 mg/mL, 1.15 mL) and HEPES buffer (375 µL). The reaction mixture was shaken at 30 °C o/n at 180 rpm before it was extracted with *n*-pentane resulting in formation of a sufficient amount of macrocyclic ether **16** (4 mg crude, 14%; due to the volatility of the products substantial loss of material can be assumed). The product was purified by column chromatography (*n*-pentane : Et_2O).

R_f = 0.50 (PE / Et_2O, 10:1); ^{1}H NMR (600 MHz, C_6D_6): δ = 5.31 – 5.29 (m, 1H, H_{16}), 5.12 (t, J = 7.7 Hz, 1H, H_4), 4.97 – 4.93 (m, 1H, H_8), 4.92 (d, J = 2.9 Hz, 2H, H_{19}), 4.07 (d, J = 11.5 Hz, 1H, H_{10}), 3.63 (dd, J = 11.3 Hz, 7.0 Hz, 1H, H_1), 3.57 (d, J = 11.5 Hz, 1H, H_{10}), 3.34 (dd, J = 11.3 Hz, 1.6 Hz, 1H, H_1), 2.36 – 2.28 (m, 4H, H_{14}, H_{15}), 2.20 – 2.16 (m, 1H, H_3), 2.14 – 2.05 (m, 2H, H_2, H_7), 2.04 – 2.01 (m, 1H, H_6), 1.98 – 1.94 (2H, m, H_6, H_7), 1.85 – 1.79 (m, 1H, H_3), 1.67 (s, 3H, H_{18}/H_{20}), 1.59 (s, 6H, H_{18}/H_{20}, H_{12}), 1.44 (s, 3H, H_{11}) ppm; ^{13}C NMR (151 MHz, C_6D_6): δ = 154.1 (C_{13}), 134.4 (C_5), 132.9 (C_9), 132.0 (C_8), 131.3 (C_{17}), 126.9 (C_4), 125.1 (C_{16}), 108.0 (C_{19}), 78.6 (C_{10}), 71.4 (C_1), 49.0 (C_2), 39.3 (C_6), 36.1 (C_{14}), 30.2 (C_3), 27.3 (C_{15}), 25.9 (C_{18}/C_{20}), 25.5 (C_7), 17.8 (C_{18}/C_{20}), 15.3 (C_{11}/C_{12}), 15.2 (C_{11}/C_{12}) ppm; HRMS [GC-MS, EI]: *m/z* calcd for $C_{20}H_{32}O$ $[M]^+$:288.2453, found:288.2455.

Biotransformation for product isolation with FPP derivative 17

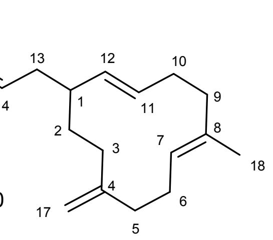

17
$C_{19}H_{30}$
258.45

FPP derivative **7** (46 mg, 0.11 mmol) and IPP **2** (50 mg, 0.17 mmol) were dissolved in an aq. NH_4HCO_3 (25 mM, 2 mL) solution. A mixture of this solution with incubation buffer (16 mL), GGPPS solution (10 mL, 13.68 mg) and SpS (12 mL, 2.05 mg) was stirred at 30 °C and 200 rpm. Shortly after starting the reaction, petroleum ether was added to the reaction mixture and shaking under these conditions was continued o/n. The phases were separated and the aqueous phase was extracted with PE (3x). The combined organic phases were dried over $MgSO_4{\cdot}H_2O$, filtered and the solvent was carefully removed *in vacuo*. The crude product was purified by column chromatography (*n*-pentane 100%) and macrocyclic terpenoid **17** (4 mg, 14%; due to the volatility of the products substantial loss of material can be assumed). was obtained with traces of *n*-pentane still present.

R_f = 0.58 (*n*-pentane 100%); ^{1}H NMR (600 MHz, C_6D_6): δ = 5.33 – 5.30 (m, 1H, H_{14}), 5.11 – 5.06 (m, 2H, H_7, H_{11}), 4.93 (dd, J = 15.2 Hz, 8.9 Hz, 1H, H_{12}), 4.86 – 4.80 (m, 2H, H_{17}), 2.30 – 2.24 (m, 2H, H_3, H_6), 2.22 – 2.18 (m, 1H, H_5), 2.15 – 1.99 (m, 8H, H_1, H_5, H_6, H_9, H_{10}, H_{13}), 1.95 – 1.83 (m, 2H, H_3, H_9), 1.74 – 1.69 (m, 1H, H_2), 1.68 (m, 3H, H_{16}), 1.59 (s, 3H, H_{19}), 1.43 (s, 3H, H_{18}), 1.16 – 1.10 (m, 1H, H_2) ppm; ^{13}C NMR (151 MHz, $CDCl_3$): δ = 151.6 (C_4), 135.2 (C_{12}), 133.5 (C_8), 131.7 (C_{15}), 130.6 (C_{11}), 127.7 (C_7), 123.8 (C_{14}), 109.1 (C_{17}), 43.7 (C_1), 39.9 (C_9), 36.5 (C_5), 34.7 (C_{13}), 32.2 (C_2), 32.0 (C_3), 30.6 (C_{10}), 29.0 (C_6), 26.0 (C_{16}), 18.0 (C_{19}), 15.1 (C_{18}) ppm; HRMS [GC-MS, CI]: *m/z* calcd for $C_{19}H_{30}$ $[M]^+$:258.2348, found: 258.2351.

Biotransformation for product isolation with FPP derivative 18a,b

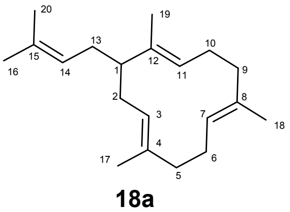

18a
$C_{20}H_{32}$
272.48

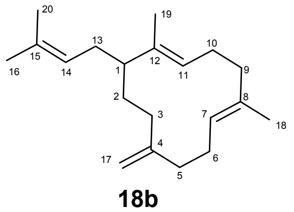

18b
$C_{20}H_{32}$
272.48

FPP derivative **8** (119 mg, 0.27 mmol) and IPP **2** (761 mg, 2.56 mmol) were dissolved in an aq. NH_4HCO_3 solution (0.05 M, 14.1 mL). The entire reaction volume (150 mL) was divided into 4 batches (2 x 50 mL and 2 x 25 mL). For simplification, the work up of a 50 mL batch will be described below. HEPES buffer (43.6 mL), Tween® 20 (10 µL), Ppase (1 µL), a GGPPS solution (5.4 mg/mL, 464 µL), a SpS solution (4.1 mg/mL, 610 µL), the FPP derivative/IPP solution (500 µL) and finally $MgCl_2$ (2 M, 250 µL) were mixed at 30 °C and 100 rpm. In intervals of 30 minutes more of the FPP derivative **8**/ IPP **2** solution (500 µL) was added up to a total volume of 4 mL. After 2 h a second batch of GGPPS (5.4 mg/mL, 462 µL) and SpS (4.1 mg/mL, 610 µL) was added. After the final substrate addition, the reaction mixture was shaken o/n. The batches were combined and extracted with *n*-pentane. The combined organic phases were dried over $MgSO_4{\cdot}H_2O$, filtered and the solvent was carefully removed *in vacuo*. The crude product was purified by column chromatography (*n*-pentane 100% → *n*-pentane:MTBE= 5:1) and a product mixture containing terpenoids **18a** + **18b** was obtained (3 mg, 4%; due to the volatility of the products

substantial loss of material can be assumed). After a first NMR recording was performed, traces of *n*-pentane were carefully co-evaporated with C_6D_6 under an inert gas stream.

Analytical data for terpenoid **18a**

R_f = 0.44 (*n*-pentane 100%); ^{1}H NMR (500 MHz, C_6D_6): δ = 5.31 – 5.27 (m, 1H, H_{14}), 4.90 (m, 2H, H_3, H_{11}), 4.81 (m, 1H, H_7), 2.29 (m, 1H, H_{10}), 2.25 (m, 1H, H_6), 2.15 (m, 2H, H_{13}), 2.14 (m, 1H, H_1), 2.13 (m, 1H, H_2), 2.13 – 1.94 (m, 4H, H_5, H_9), 2.04 (m, 1H, H_2), 1.96 (m, 1H, H_6), 1.93 (m, 1H, H_{10}), 1.68 (s, 3H, H_{16}/H_{20}), 1.60 (s, 3H, H_{16}/H_{20}), 1.47 (s, 3H, H_{17}), 1.46 (s, 3H, H_{18}), 1.39 (t, *J* = 1.2 Hz, 3H, H_{19}) ppm; ^{13}C NMR (126 MHz, $CDCl_3$): δ = 136.2 (C_{12}), 132.9 (C_8), 132.6 (C_4), 131.4 (C_{15}), 127.1 (C_7), 127.1 (C_{11}), 126.7 (C_3), 124.4 (C_{14}), 51.1 (C_1), 40.2 (C_5), 40.2 (C_9), 32.6 (C_{13}), 31.7 (C_2), 26.0 (C_{16}/C_{20}), two signals of 25.6/25.4/25.2 (C_6, C_{10}), 18.0 (C_{16}/C_{20})15.4 (C_{17}), 15.4 (C_{18}), 11.4 (C_{19}) ppm.

Analytical data for terpenoid **18b**

R_f = 0.44 (*n*-pentane 100%); ^{1}H NMR (500 MHz, C_6D_6): δ = 5.31 – 5.27 (m, 1H, H_{14}), 5.03 (m, 1H, H_7), 4.95 (m, 1H, H_{11}), 4.85 (m, 2H, H_{17}), 2.25 (m, 1H, H_{10}), 2.23 (m, 1H, H_6), 2.21 (m, 1H, H_1), 2.21 – 2.10 (m, 2H, H_5), 2.13 – 1.94 (m, 2H, H_9), 2.11 (m, 2H, H_{13}), 2.07 (m, 1H, H_3), 2.03 (m, 1H, H_6), 1.99 (m, 1H, H_{10}), 1.83 (m, 1H, H_3), 1.70 – 1.65 (m, 1H, H_2), 1.68 (s, (3H, H_{16}/H_{20}), 1.60 (s, 3H, H_{16}/H_{20}), 1.45 (s, 3H, H_{19}), 1.44 (s, 3H, H_{18}), 1.41 – 1.34 (m, 1H, H_2) ppm; ^{13}C NMR (126 MHz, $CDCl_3$): δ = 150.6 (C_4), 135.9 (C_{12}), 133.5 (C_8), 131.4 (C_{15}), 127.2 (C_7), 127.1 (C_{11}), 124.2 (C_{14}), 109.0 (C_{17}), 48.7 (C_1), 39.9 (C_9), 36.8 (C_5), 33.3 (C_{13}), 31.4 (C_3), 29.9 (C_2), 27.7 (C_6), 26.0 (C_{16}/C_{20}),), one signal of 25.6/25.4/25.2 (C_{10}), 18.0 (C_{16}/C_{20}), 15.1 (C_{18}), 12.6 (C_{19}) ppm;

For one compound each: HRMS [GC-MS, EI]: *m/z* calcd for $C_{20}H_{32}$ $[M]^+$: 272.2504, found: 272.2507. and HRMS [GC-MS, EI]: *m/z* calcd for $C_{20}H_{32}$ $[M]^+$: 272.2504, found: 272.2511.

1.4 GC-MS data

When employing the GGPPS-SpS enzyme cascade different FPP-derivatives were tested. FPP derivatives that showed product formation on an analytical scale suitable for structure elucidation as judged by GC-MS analysis are depicted in the following.

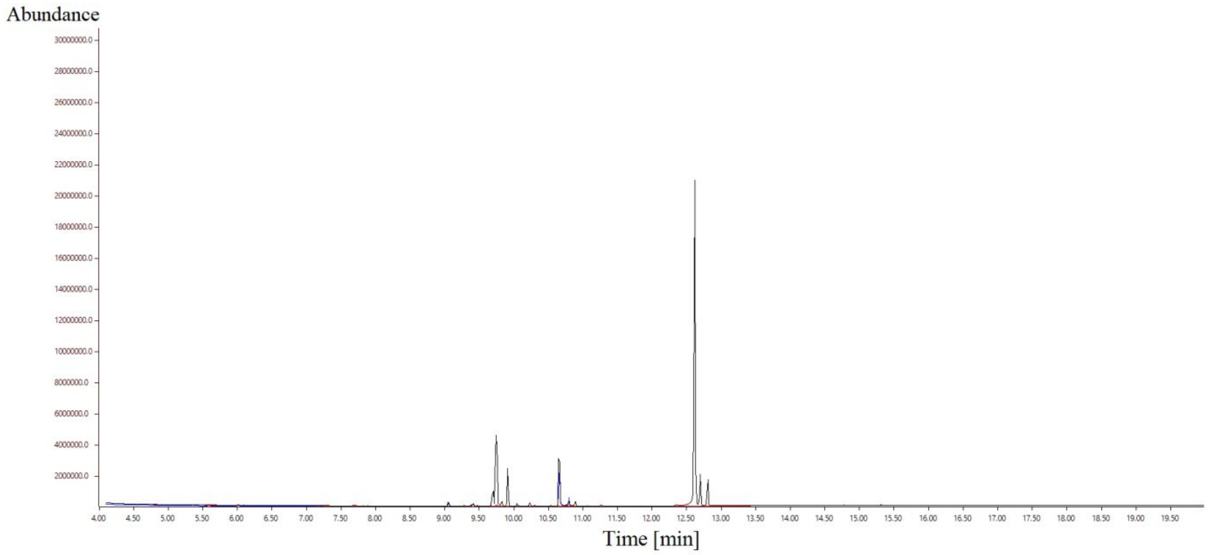

Figure S2. GC-data for the biotransformation of GGPPS-SpS cascade with FPP derivative **6** (black), and negative controls without enzyme (blue) and without derivative (red) (Agilent 5977B GC/MSD with 7890B GC-system).

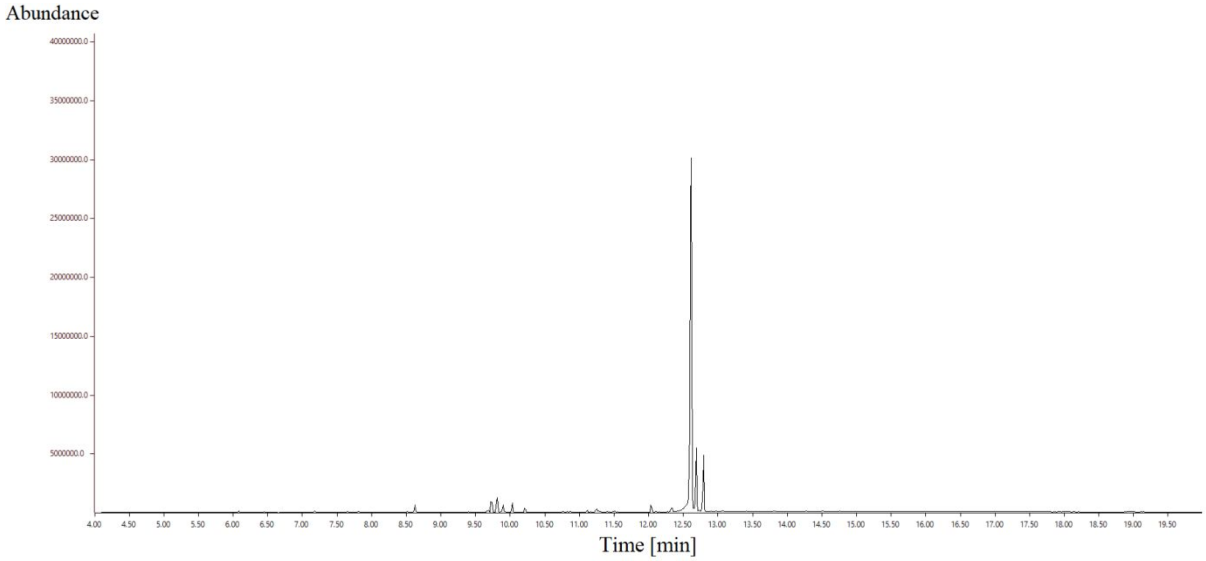

Figure S3. GC-data for the biotransformation product of GGPPS-SpS cascade with FPP derivative **6** (Agilent 5977B GC/MSD with 7890B GC-system).

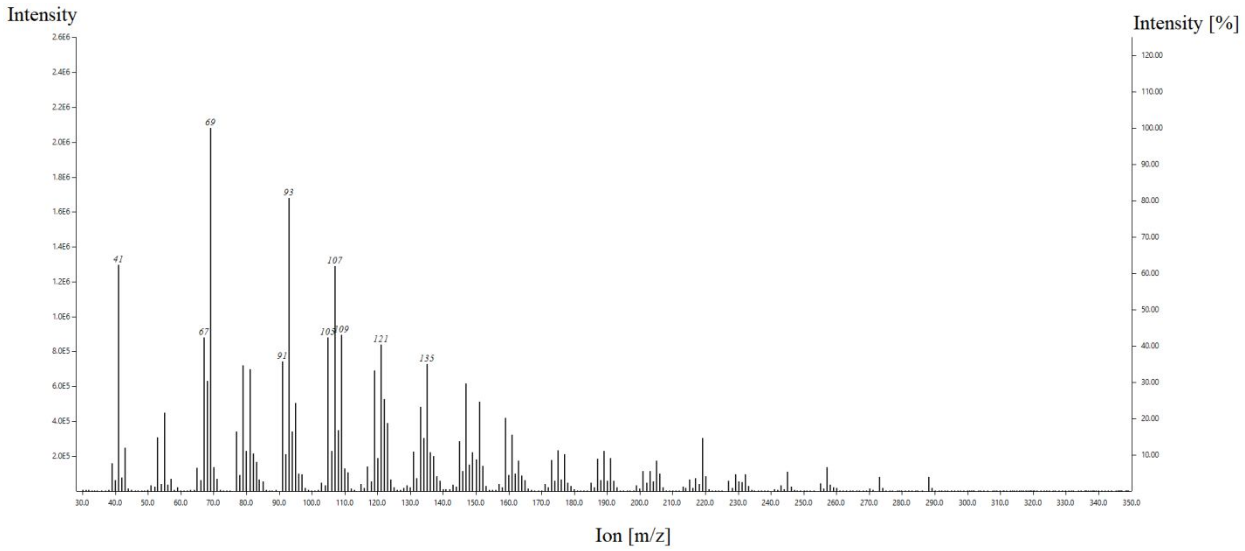

Figure S4. Mass spectrum for GC signal at 12.617 min obtained from the biotransformation product of **6** with the GGPPS-SpS cascade.

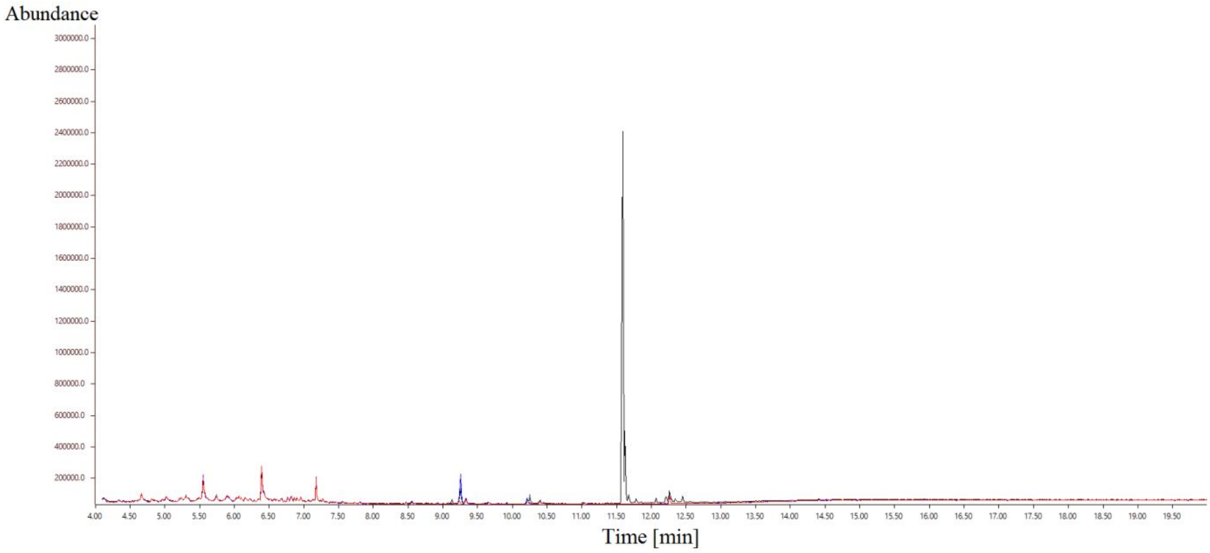

Figure S5. GC-data for the biotransformation of GGPPS-SpS cascade with FPP derivative **7** (black), and negative controls without enzyme (blue) and without derivative (red) **(**Agilent 5977B GC/MSD with 7890B GC-system).

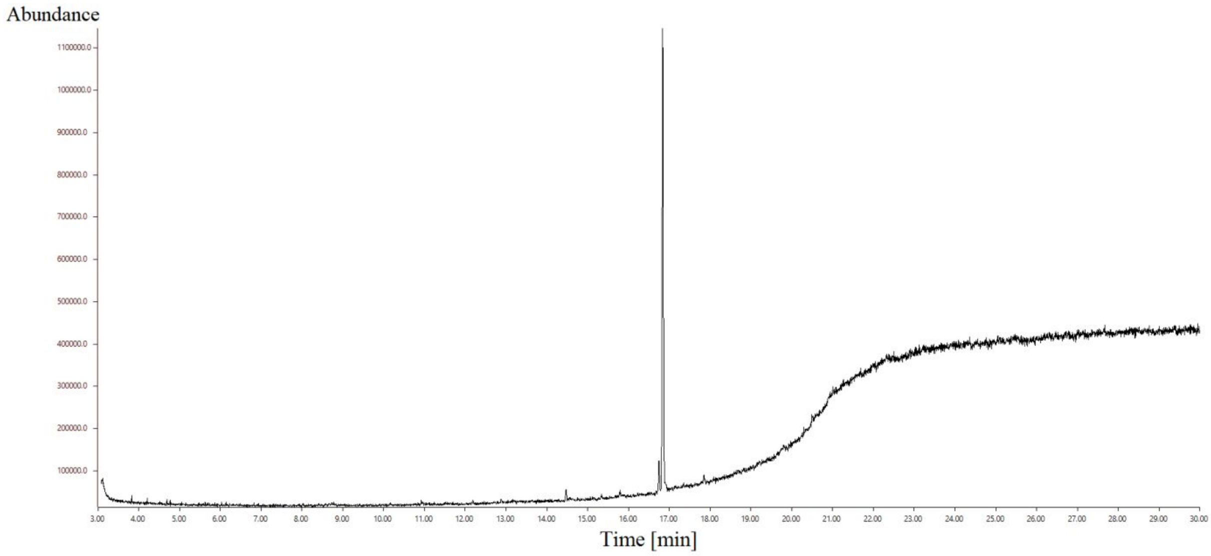

Figure S6. GC data for the biotransformation product of GGPPS-SpS cascade with FPP derivative **7** **(**GC-MS HP MSD-5973 / GC-6890).

For a better comparison of the different products the GC data of the purified product **7** conducted with the Agilent 5977B GC/MSD coupled to the 7890B GC-system is shown below.

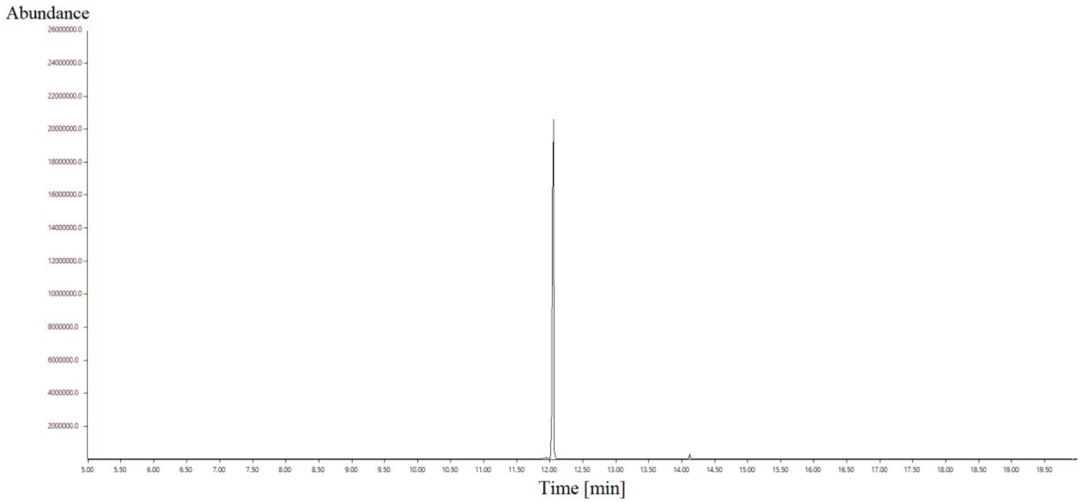

Figure S7. GC data for the biotransformation product of GGPPS-SpS cascade with FPP derivative **7** **(**Agilent 5977B GC/MSD with 7890B GC-system).

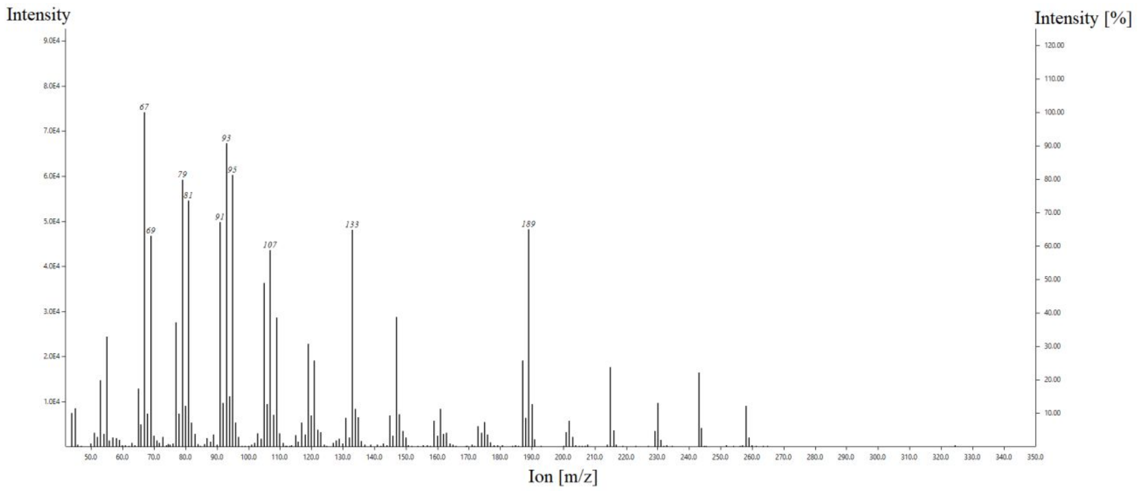

Figure S8. Mass spectrum for GC signal at 16.840 min obtained from the biotransformation of **7** with the GGPPS-SpS cascade.

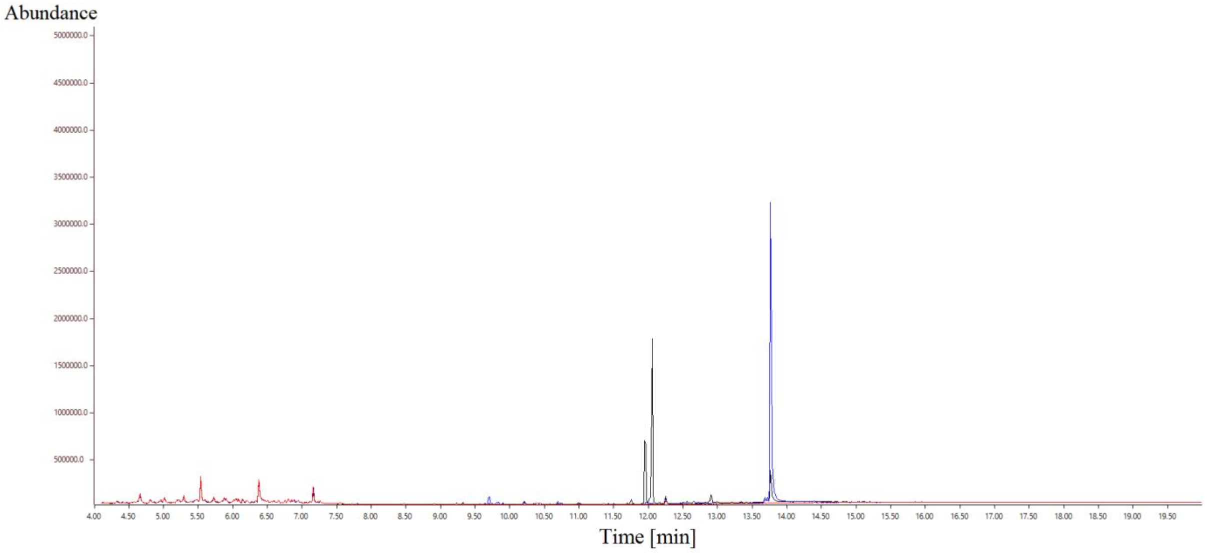

Figure S9. GC-data for the biotransformation of GGPPS-SpS cascade with FPP derivative **8** (black), and negative controls without enzyme (blue) and without derivative (red) **(**Agilent 5977B GC/MSD with 7890B GC-system).

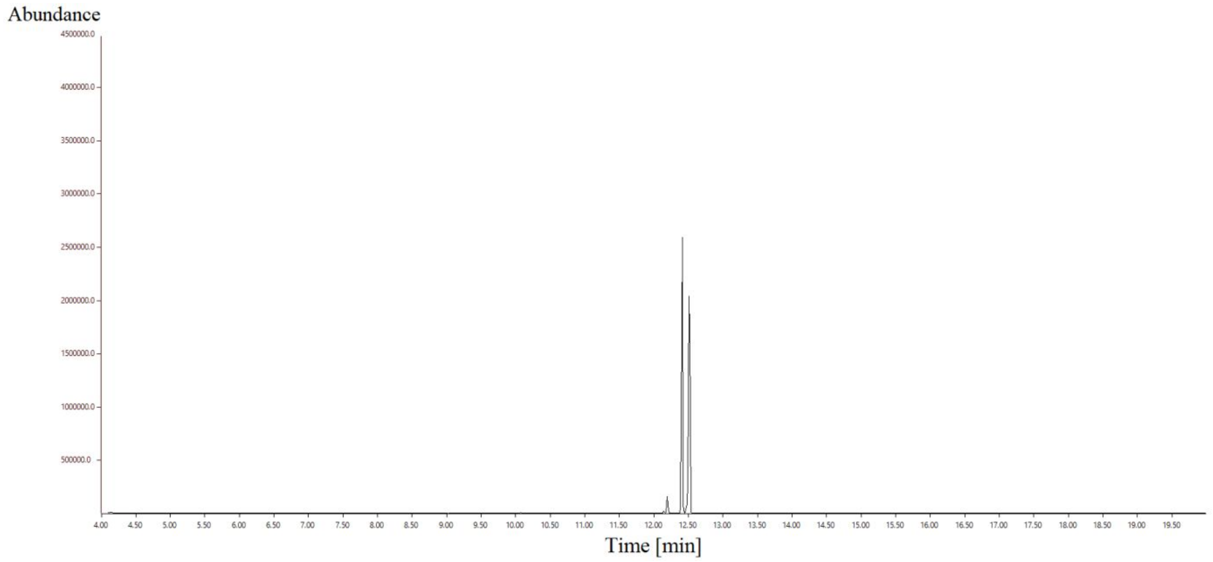

Figure S10. GC data for the purified product mixture from the biotransformation of GGPPS-SpS cascade with FPP derivative **8** (Agilent 5977B GC/MSD with 7890B GC-system). The ratio was determined to be 1:1.1.

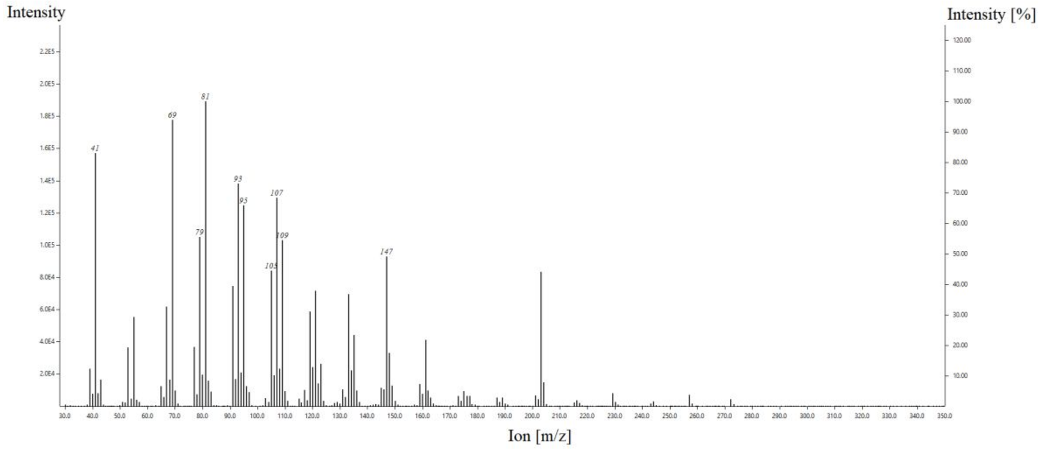

Figure S11. Mass spectrum for GC signal at 12.412 min obtained from the biotransformation of **8** with the GGPPS-SpS cascade.

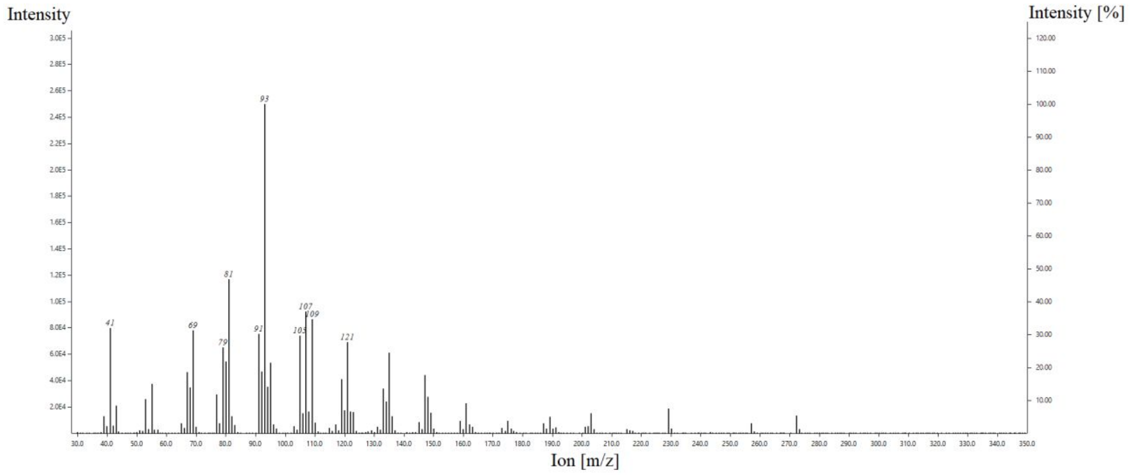

Figure S12. Mass spectrum for GC signal at 12.508 min obtained from the biotransformation of **8** by GGPPS and SpS.

1.5 Structure elucidation of macrocyclic ether 16

Copies of ^{1}H-NMR and ^{13}C spectra are found in section 2. Copies of NMR spectra.

Table S1. ^{1}H NMR signals and the corresponding ^{13}C NMR signals as analyzed by ^{1}H-^{13}C HSQC and ^{13}C{^{1}H} DEPT135 measurements. The quaternary carbon atoms are listed at the bottom.

δ (^{1}H)/ppm	δ (^{13}C)/ppm	DEPT135/HSQC phase
1.44	15.2 or 15.3	CH/CH_3
1.59	15.2 or 15.3	CH/CH_3
1.59	25.9 or 17.7	CH/CH_3
1.67	25.9 or 17.7	CH/CH_3
1.85 – 1.79	30.2	CH_2
1.98 – 1.94	25.5 and 39.3	CH_2 and CH_2
2.04 – 2.01	39.3	CH_2
2.14 – 2.05	25.5 and 49.0	CH_2 and CH/CH_3
2.20 – 2.16	30.2	CH_2
2.36 – 2.28	36.1 and 27.3	CH_2 and CH_2
3.63 + 3.34	71.4	CH_2
4.07 + 3.57	78.6	No information
4.92	108.0	CH_2
4.99 - 4.97	132.0	CH/CH_3
5.12	126.9	CH/CH_3
5.31 – 5.29	125.1	CH/CH_3
	131.3	C_{quart}
	132.0	C_{quart}
	132.9	C_{quart}
	134.4	C_{quart}
	154.1	C_{quart}

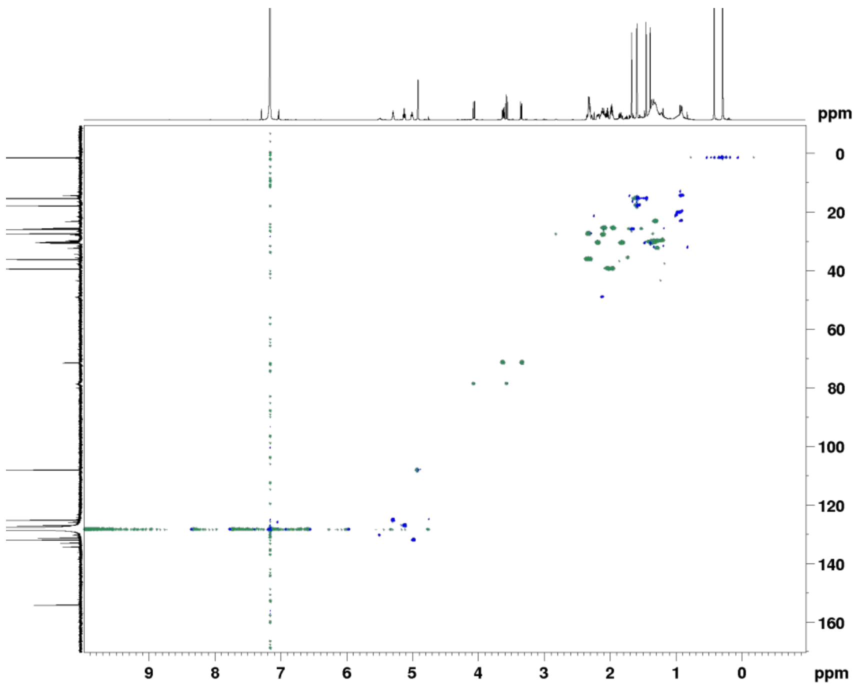

Figure S13. ^{1}H-^{13}C HSQC NMR spectrum of macrocyclic ether **16** in C_6D_6 (pos. phase = blue (CH/CH_3), neg. phase = green (CH_2)) at $T = 310.0$ K.

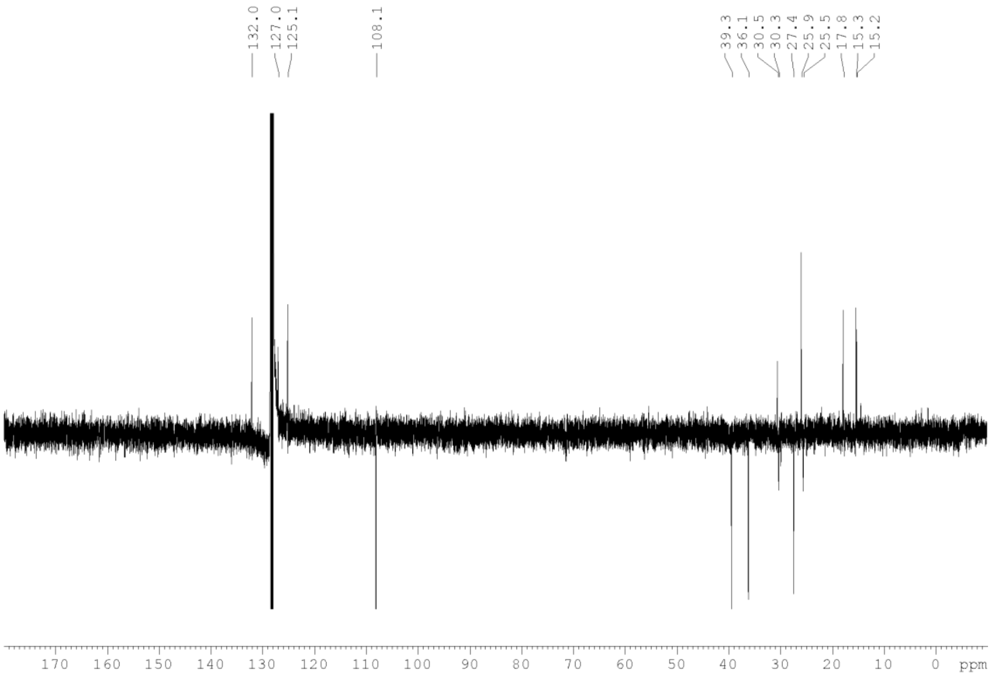

Figure S14. $^{13}C\{^{1}H\}$ DEPT135 NMR spectrum of macrocyclic ether **16** in C_6D_6.

Table S2: ^{1}H NMR signals and their corresponding ^{1}H-^{1}H COSY correlation signals for macrocyclic ether **16**. Weak signals are given in parentheses. Signals found in the aromatic region likely are associated with minor impurities present in C_6D_6 employed.

δ (^{1}H)/ppm	COSY correlations
5.31 – 5.29	1.59, 1.67, 2.36 – 2.28
5.12	1.44, 1.85 – 1.79, 2.20 – 2.16
4.99 - 4.97	1.59, 1.98 – 1.94, 2.06 – 2.11,
4.92	(2.36 – 2.28)
4.07	3.57
3.63	2.14 – 2.05, 3.34
3.57	4.07
3.34	3.63
2.36 – 2.28	1.59, 1.67, 4.92, 5.31 – 5.29
2.20 – 2.16	(1.44), 1.85 – 1.79, 5.12
2.14 – 2.05	1.85 – 1.79, 1.98 – 1.94, (3.34), 3.63, 4.99 - 4.97
2.04 – 2.01	hard to distinguish
1.98 – 1.94	4.99 - 4.97, 2.14 – 2.05
1.85 – 1.79	2.14 – 2.05, 2.20 – 2.16, 5.12
1.67	1.59, 2.36 – 2.28, 5.31 – 5.29
1.59	5.31 – 5.29, 4.99 - 4.97, 2.36 – 2.28, 1.67
1.44	5.12

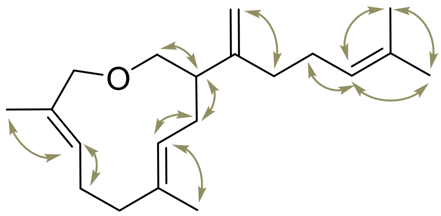

Figure S15. Key ^{1}H-^{1}H COSY NMR of macrocyclic ether **16** indicated by arrows.

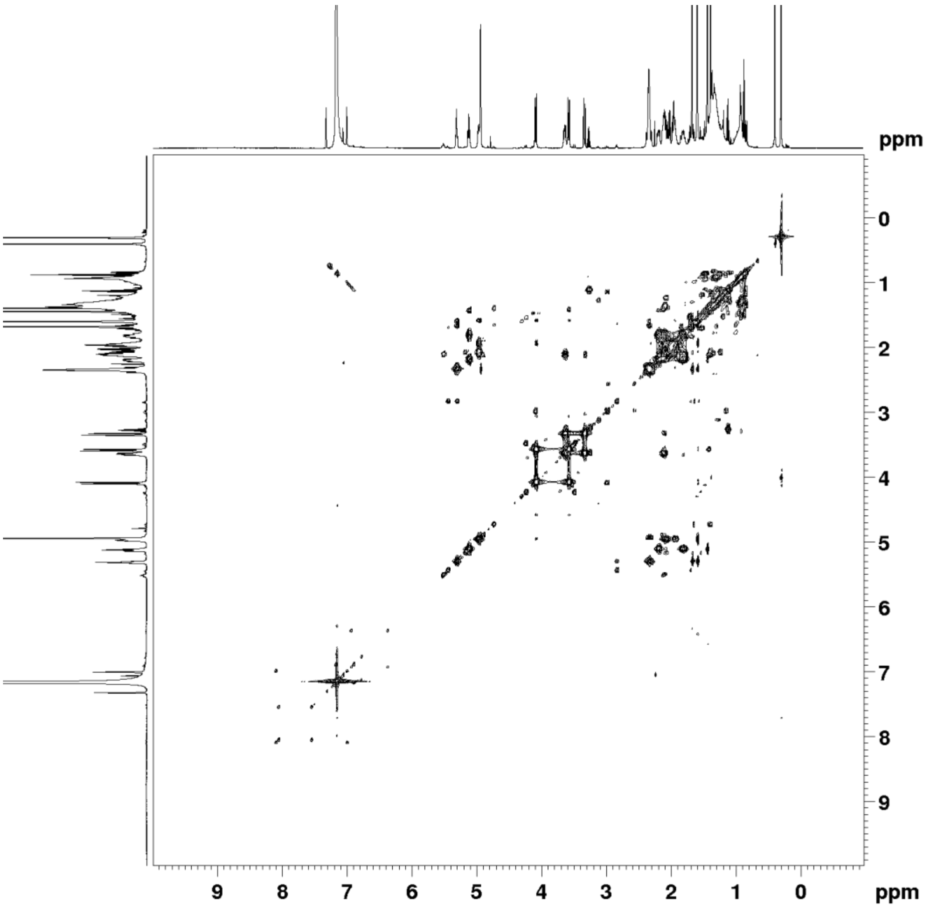

Figure S16. ^{1}H-^{1}H COSY NMR spectrum of macrocyclic ether **16** in C_6D_6.

Table S3: ^{1}H NMR signals correlated with ^{13}C NMR signals obtained from ^{1}H-^{13}C HMBC measurements.

δ (^{1}H)/ppm	δ (^{13}C)/ppm	δ (^{1}H)/ppm	δ (^{13}C)/ppm
5.31 – 5.29	17.8, 25.9, 27.3	2.20 – 2.16	71.4, 126.9, 134.4
5.12	15.2 or 15.3, 30.2, 39.3,	2.14 – 2.05	39.3
4.99 - 4.97	15.2 or 15.3	2.04 – 2.01	15.2 or 15.3, 25.5, 126.9, 132.0, 134.4
4.92	49.0, 36.1, 154.1	1.98 – 1.94	15.2 or 15.3, 25.5, 126.9, 132.0, 134.4
4.07	15.2 or 15.3, 71.4, 132.0, 132.9	1.85 – 1.79	126.9, 134.4
3.63	154.1	1.67	17.8, 125.1, 131.3
3.57	15.2 or 15.3, 71.4, 132.0	1.59	25.9, 78.6, 125.1, 131.3, 132.0, 132.9
3.34	30.2, 49.0, 78.6, 154.1	1.44	39.3, 126.9, 134.4
2.36 – 2.28	27.3, 36.1, 108.0, 125.1, 131.3, 154.1		

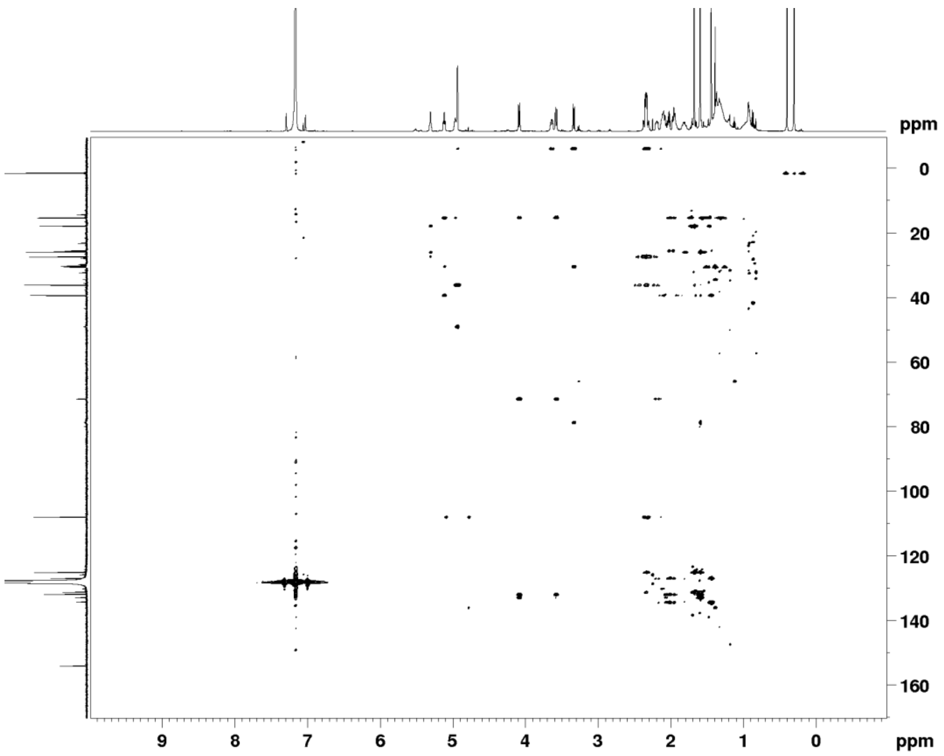

Figure S17. ^{1}H-^{13}C HMBC NMR spectrum of macrocyclic ether **16** in C_6D_6 at T =298.0 K.

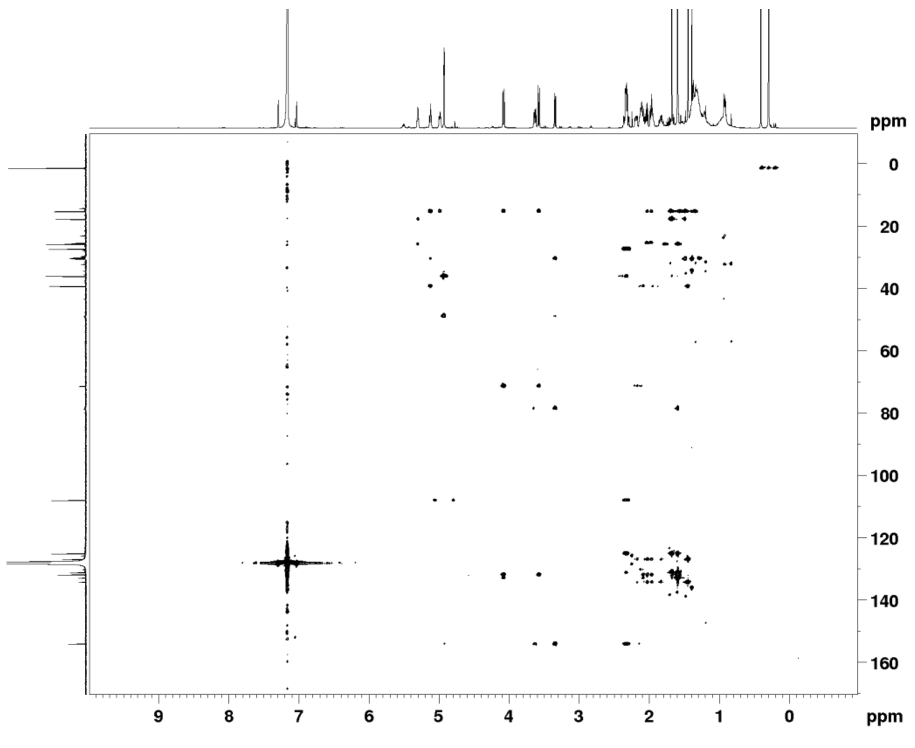

Figure S18. ^{1}H-^{13}C HMBC NMR spectrum of macrocyclic ether **16** in C_6D_6 at T = 310.0 K.

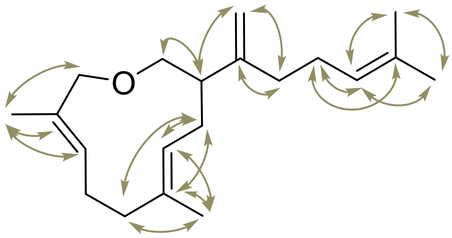

Figure S19. Key ^{1}H-^{13}C HMBC NMR correlations of **16** indicated by arrows.

Figure S20. Full assignment of NMR spectra for **16**.

.

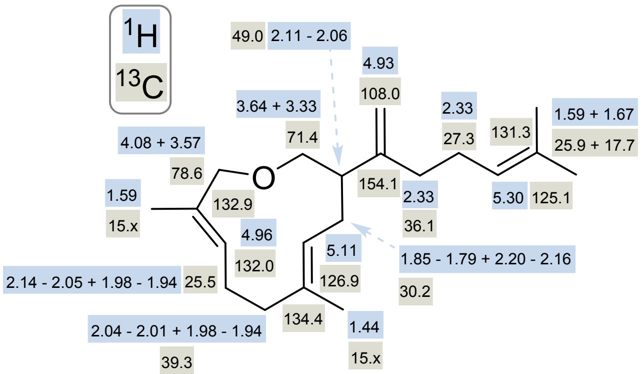

The structure elucidation of macrocyclic ether **16** revealed that carbon atoms bound to ether oxygen atom and the carbon atom of the stereogenic center could not be detected in the 1D ^{13}C NMR spectra. However, in the HSQC and HMBC, spectra signals between the protons and the missing ^{13}C carbon signals were clearly detectable. To gain more information on these ^{13}C signals, we performed high temperature NMR measurements. These furnished a small increase of the signal intensity and allowed to assign the ^{1}H data with the corresponding carbon atoms. The assignment of C-4 and C-5 is based on the fact that C-4 shows a COSY correlation to the proton at δ= 1.44 ppm (a methyl group). This is a typical pattern for trisubstituted olefins in terpenes. Since the protons of that methyl group also correlate with the quaternary carbon at δ= 126.9 ppm (C-4) as well as with δ= 134.4 ppm (C-5) in the HMBC spectrum in can be concluded that the protons at δ= 1.44 ppm and the C atom δ= 134.4 ppm are linked. In order to determine the geometry of the newly formed tri-substituted double bond we performed an 1D NOE analysis. If the double bond would be (*Z*)-configured, a NOE correlation between the protons at δ= 1.44 ppm and δ= 5.12 ppm could have been expected. This correlation was not observed so that an (*E*)-configuration can be assumed. This is supported by a weak correlation between δ= 1.44 ppm and δ= 2.20-2,16 ppm.

Regarding the macrocyclic ether **16** four different low energy conformations can be suggested (s. figure **21**).

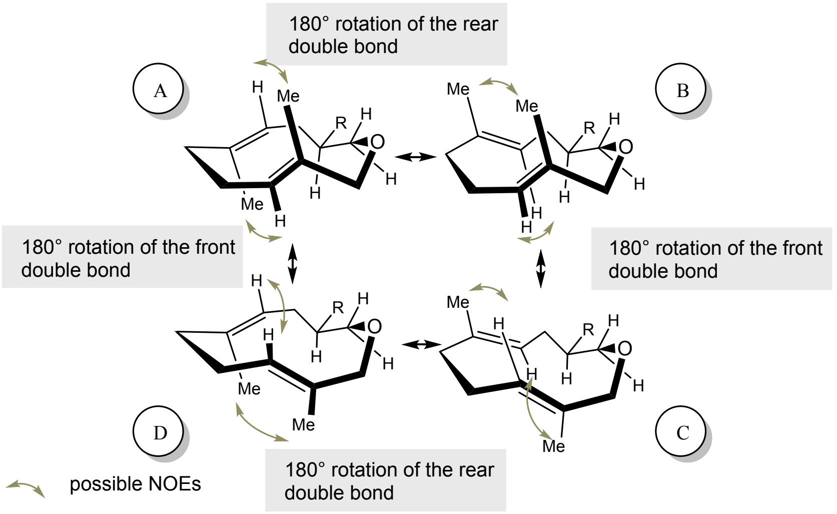

Figure S21. Possible conformations A-D of macrocyclic ether **16** to be considered prior to NOE analysis.

When analyzing the 1D NOE experiments of $H_{1.44}$ one would expect either a strong correlation between $H_{1.44}$ and $H_{4.99-4.97}$ if the methyl groups are *anti* orientated or between $H_{1.44}$ and $H_{1.59}$ if they are *syn* orientated. As the 1D NOE shows a clear proton correlation between signals at δ= 1.44 ppm and δ= 1.59 ppm, we suggest that the conformations on the top right (B) or bottom left (D) are likely to be present. Due to proton overlap in thh ^{1}H NMR spectrum it was not possible to unequivocally determine which of these two conformations is the preferred one.

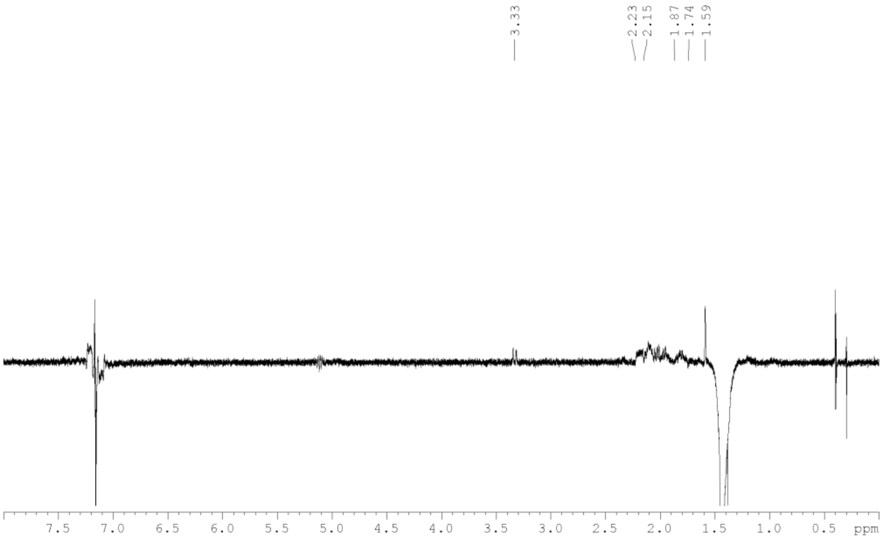

Figure S22. 1D NOE spectra of compound **16** with irradiation at δ= 1.43 ppm.

1.6 Structure elucidation of compound 17

Copies of ^{1}H-NMR and ^{13}C spectra are found in section 2 (Copies of NMR spectra).

Table S4. ^{1}H NMR signals and their corresponding ^{13}C NMR signals as analyzed with the support of ^{1}H-^{13}C HSQC and ^{13}C{^{1}H} DEPT135 experiments. The quaternary carbon atoms are listed at the bottom.

δ (^{1}H)/ppm	δ (^{13}C)/ppm	DEPT135/HSQC phase
5.33 – 5.30	123.8	CH/CH_3
5.11 – 5.06	130.6, 127.7	CH/CH_3, CH/CH_3
4.93	135.2	CH/CH_3
4.86 – 4.80	109.1	CH_2
2.30 – 2.24	32.0, 29.0	CH_2, CH_2
2.22 – 2.18	36.5	CH_2
2.15 – 1.99	43.7, 39.9, 36.5, 34.7, 30.6, 29.0	CH/CH_3, CH_2, CH_2, CH_2, CH_2, CH_2
1.95 – 1.83	39.9, 32.0	CH_2, CH_2
1.74 – 1.69	32.2	CH_2
1.68	26.0	CH/CH_3
1.59	18.0	CH/CH_3
1.43	15.1	CH/CH_3
1.16 – 1.10	32.2	CH_2
	151.6	C_{quart}
	133.5	C_{quart}
	131.7	C_{quart}

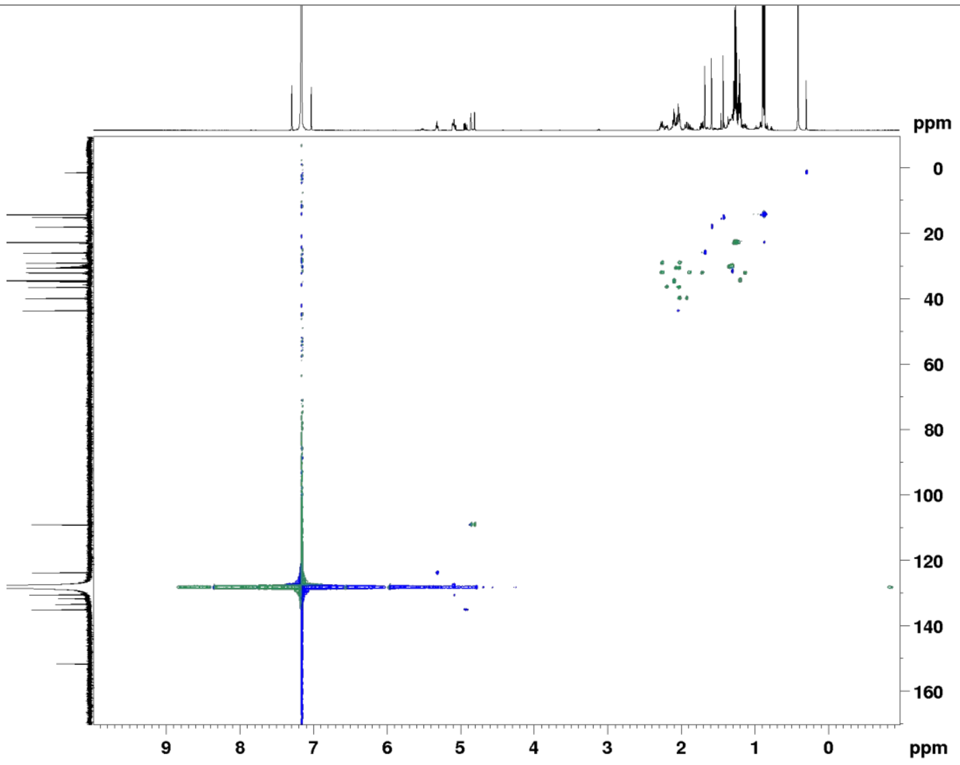

Figure S23. ^{1}H-^{13}C HSQC NMR spectrum of compound **17** in C_6D_6 (pos. phase = blue (CH/CH_3), neg. phase = green (CH_2)).

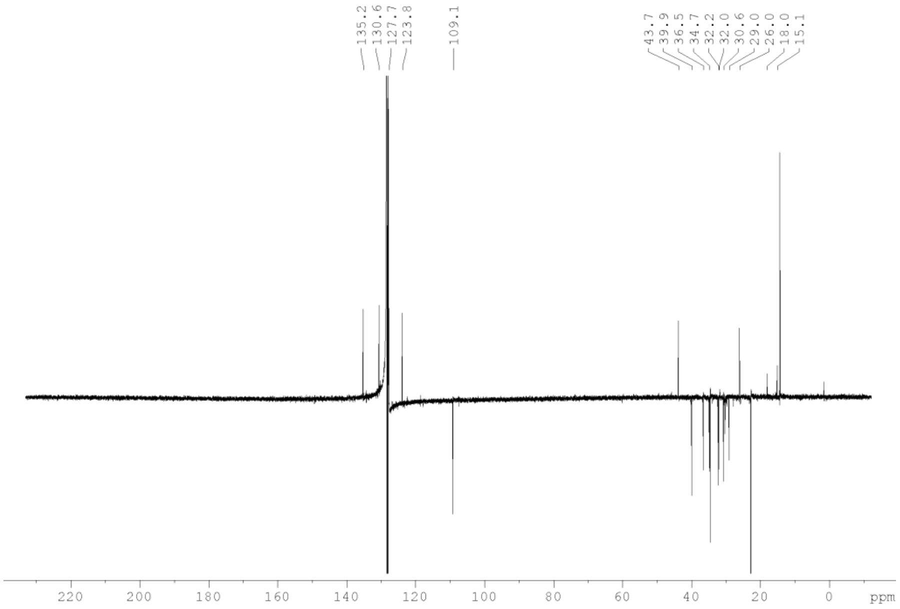

Figure S24. $^{13}C\{^{1}H\}$ DEPT135 NMR spectrum of compound **17** in C_6D_6.

Table S5: ^{1}H NMR signals and the corresponding ^{1}H-^{1}H COSY correlations for compound **17**. Signals with weak intensities are given in parentheses.

δ (^{1}H)/ppm	COSY correlations
5.33 – 5.30	1.59, 1.68, 2.15 – 1.99 at ≈ 2.10
5.11 – 5.06	1.43, 1.95 – 1.83, 2.15 – 1.99 at ≈ 2.04, 2.26, 4.93
4.93	2.04, 5.11 – 5.06
4.86 – 4.80	2.30 – 2.24, 2.22 – 2.18, 1.95 – 1.83
2.30 – 2.24	5.11 – 5.06, 4.86 – 4.80, 2.22 – 2.18
2.22 – 2.18	4.86 – 4.80, 2.30 – 2.24, 2.15 – 1.99
2.15 – 1.99	5.33 – 5.30, 5.11 – 5.06, 4.93, 4.86 – 4.80, 2.15 – 1.99, 1.95 – 1.83, 1.74 – 1.69, 1.16 – 1.10, 1.68, 1.59
1.95 – 1.83	4.86 – 4.80, 2.30 – 2.24, 2.15 – 1.99, 1.74 – 1.69, 1.16 – 1.10
1.74 – 1.69	2.30 – 2.24, 2.15 – 1.99, 1.95 – 1.83, 1.16 – 1.10
1.68	5.33 – 5.30, 2.10, 1.58
1.59	5.33 – 5.30, 2.10, 1.68
1.43	5.11 – 5.06, 2.15 – 1.99
1.16 – 1.10	2.30 – 2.24, 2.15 – 1.99, 1.95 – 1.83, 1.74 – 1.69

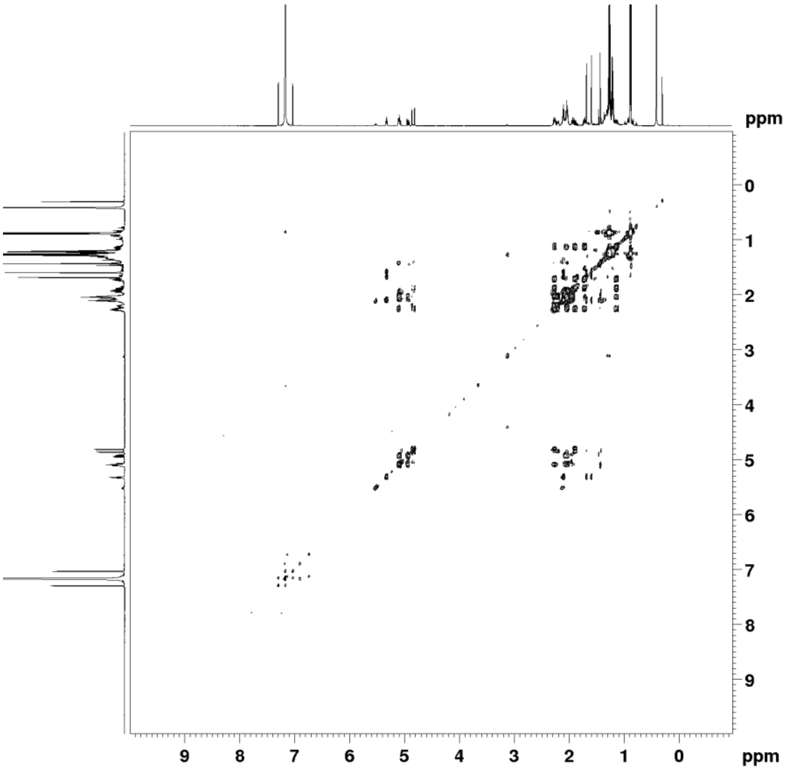

Figure S25. ¹H-¹H COSY NMR spectrum of compound **17** in C_6D_6.

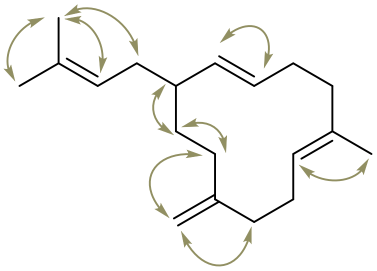

Figure S26. Key ¹H-¹H COSY NMR correlations for **17** indicated by arrows.

Table S6: Selected correlations between ¹³C NMR signals and neighbouring ¹H NMR signals as collected from the ¹H-¹³C HMBC spectrum of compound **17**. Note: Signals with weak intensities are given in parentheses.

δ (¹H)/ppm	δ (¹³C)/ppm
5.33 – 5.30	18.0, 26.0
4.93	30.6, (130.6)
4.86 – 4.80	32.0, 36.5
2.30 – 2.24	36.5, 109.1, 127.7, 133.5, 151.6,
2.15 – 1.99	32.x, 39.9, 43.7, 123.8, 131.7, 135.2, 151.6
1.95 – 1.83	30.6, 32.x, 109.1, 133.6, 151.6
1.74 – 1.69	(43.7), 135.2
1.68	18.0, 123.8, 131.7

δ (^{1}H)/ppm	δ (^{13}C)/ppm
1.59	26.0, 123.8, 131.7
1.43	39.9, 127.7, 133.5

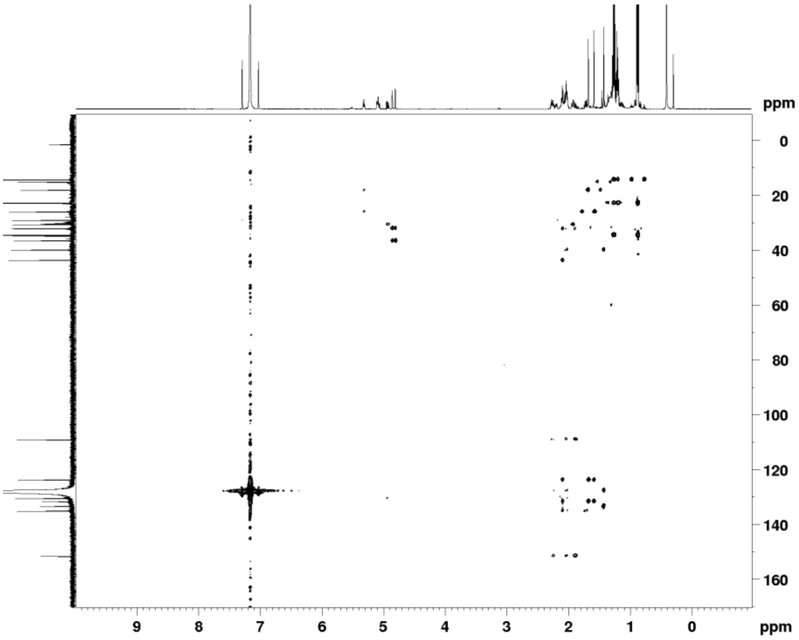

Figure S27. ^{1}H-^{13}C HMBC NMR spectrum of compound **17** in C_6D_6.

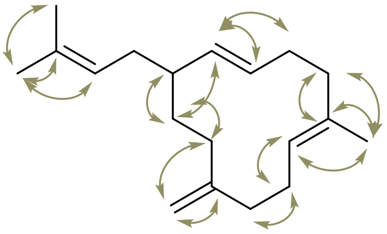

Figure S28. Key ^{1}H-^{13}C HMBC NMR correlations of **17** as indicated by arrows.

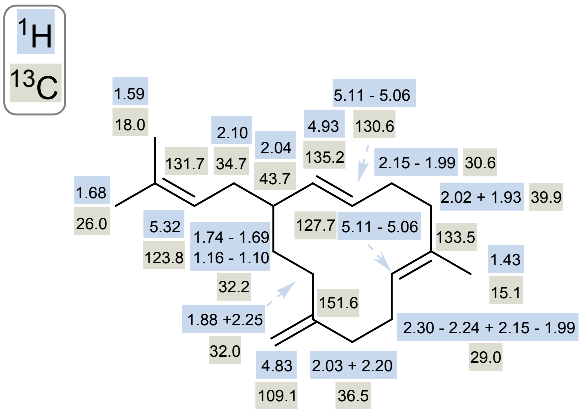

Details of the structure elucidation: The proton at δ= 1.43 ppm (methyl group at C-8) showed a HMBC correlation to the carbon atom δ= 39.9 ppm (C-9) and d= 133.5 ppm (C-8). This is a typical pattern for trisubstituted olefines in terpenes.

Figure S29. Full assignment of NMR spectra for **17**.

1.7 Structure elucidation of compound mixtures 18a and 18b

Copies of ^{1}H-NMR and ^{13}C spectra are found in section 2 (Copies of NMR spectra).

The structure elucidation could only be performed with the inseparable mixture of isomers.

Table S7. ^{1}H NMR signals with their corresponding ^{13}C NMR signals as determined by ^{1}H-^{13}C HSQC and ^{13}C{^{1}H} DEPT135 experiments. The quaternary carbon atoms are listed at the bottom. In cases were the chemical shifts (δ) of ^{13}C signals are very close, an x is used as a label. Carbon atoms showing two signals with very similar δ values are marked with (d).

δ (^{1}H)/ppm	δ (^{13}C)/ppm	DEPT135/HSQC phase	Part of structure **18a/18b**
5.31 – 5.27	124.2 (d)	CH/CH_3	**18a**, **18b**
5.03	127.2	CH/CH_3	**18b**
4.95	127.1	CH/CH_3	**18b**
4.90	127.1, 126.7	CH/CH_3	**18a**
4.85	109.0	CH_2	**18b**
4.81	127.1	CH/CH_3	**18a**
2.29 + 1.93	25.x	CH_2	**18a**
2.25 + 1.99	25.x	CH_2	**18b**
2.25 + 1.96	25.x	CH_2	**18a**
2.23 + 2.03	27.7	CH_2	**18b**
2.21	48.7	CH/CH_3	**18b**
2.21 – 2.10	36.8	CH_2	**18b**
2.15	32.6	CH_2	**18a**
2.14	51.1	CH/CH_3	**18a**
2.13 – 1.94	39.9, 40.2, 40.2	CH_2	**18a, 18b**
2.13 + 2.04	31.7	CH_2	**18a**
2.11	33.3	CH_2	**18b**
2.07 + 1.83	31.4	CH_2	**18b**
1.70 – 1.65 + 1.41 – 1.34	29.9	CH_2	**18b**
1.68	26.0 (d)	CH_3	**18a, 18b**
1.60	18.0 (d)	CH_3	**18a, 18b**
1.47	15.4	CH_3	**18a**

1.46	15.4	CH_3	**18a**
1.45	12.6	CH_3	**18b**
1.44	15.1	CH_3	**18b**
1.39	11.4	CH_3	**18a**
	131.4 (d)	C_{quart}	**18a, 18b**
	132.6	C_{quart}	**18a**
	132.9	C_{quart}	**18a**
	133.5	C_{quart}	**18b**
	135.9	C_{quart}	**18b**
	136.2	C_{quart}	**18a**
	150.6	C_{quart}	**18b**

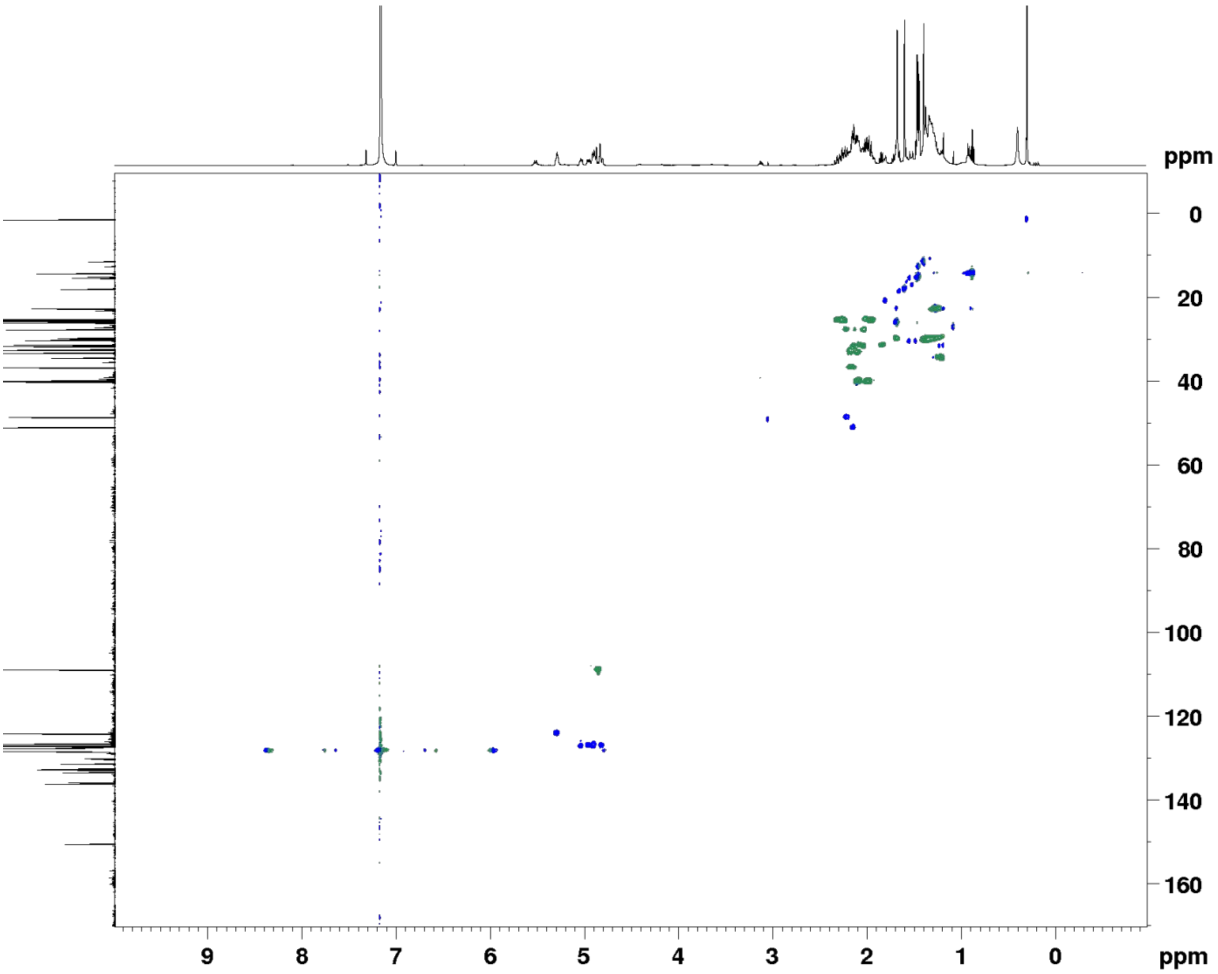

Figure S30. ^{1}H-^{13}C HSQC NMR spectrum of compound mixture **18a** and **18b** in C_6D_6 (pos. phase = blue (CH/CH_3), neg. phase = green (CH_2)).

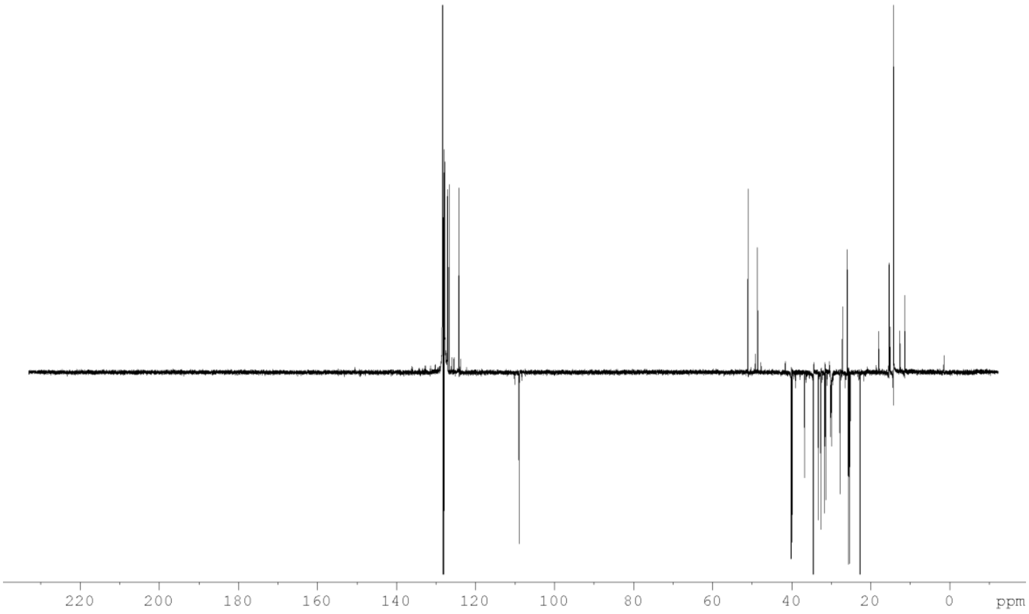

Figure S31. $^{13}C\{^{1}H\}$ DEPT135 NMR spectrum of compound mixtures **18a** and **18b** in C_6D_6.

Table S8: Selected ^{1}H NMR signals and their corresponding ^{1}H-^{1}H COSY correlation signals for compound mixture **18a**,**b**. Weak signals are given in parentheses.

δ (^{1}H)/ppm	COSY correlations
5.31 – 5.27	1.60, 1.68, area from 2.22 to 2.05
5.03	1.44, 2.03, 2.23
4.95	1.45, 2.25 + 1.99
4.90	1.39, 1.47, 2.29 + 1.93, 2.13 + 2.04
4.85	(2.07)
4.81	1.46, 2.25 + 1.96
2.07 + 1.83	1.41 – 1.34, 1.70 – 1.65, 4.81
1.70 – 1.65 + 1.41 – 1.34	1.83, 2.07, 2.21
1.68	1.60, 5.31 – 5.27
1.60	1.68, 5.31 – 5.27
1.47	4.90
1.46	4.81
1.45	4.95
1.44	5.03
1.39	4.90

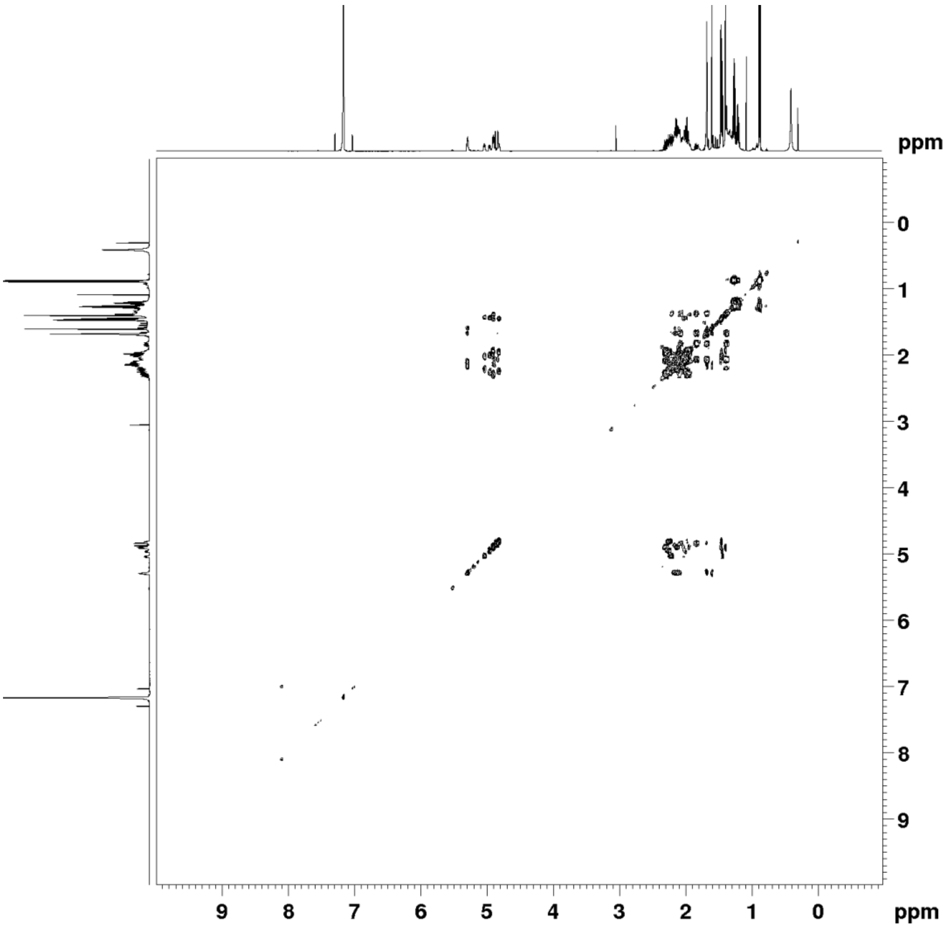

Figure S32. ¹H-¹H COSY NMR spectrum of compound mixture **18a** and **18b** in C_6D_6.

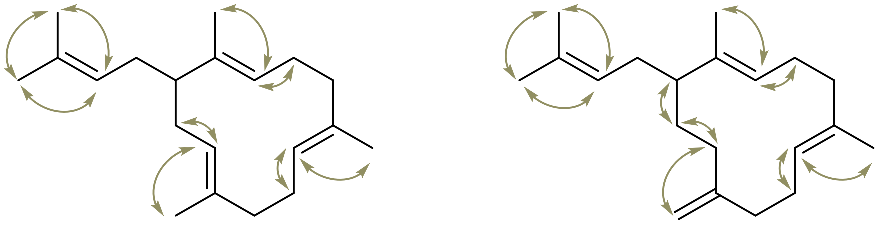

Figure S33. Key ¹H-¹H COSY NMR correlations for compound mixture **18a** and **18b** indicated by arrows.

Table S9: Selected correlations between neighboring ¹³C and ¹H atoms as determined from ¹H-¹³C HMBC data of the mixture of **18a** and **18b**. Note: Weak signals are given in parentheses.

δ (¹H)/ppm	δ (¹³C)/ppm
5.31 – 5.27	18.0, 26.0, (32.6 ,33.3, 48.7, 51.1)
5.03	15.1, 27.7, (36.8), 39.9
4.95	12.6, 48.7
4.90	11.4, 15.4, (25.x), 40.2, 51.1
4.85	31.4, 36.8
4.81	15.4, (25.x), 40.2

δ (^{1}H)/ppm	δ (^{13}C)/ppm
2.23 + 2.03	36.8
2.07 + 1.83	29.9, (36.8, 48.7), 109.0, 150.6
1.68	18.0, (48.7, 51.1), 124.2, 131.4
1.60	26.0, 124.2, 131.4
1.47	40.2, 132.6, 126.7
1.46	40.2, 127.1, 132.9
1.45	48.7, 135.9
1.44	39.9, 127.2, 133.5
1.39	51.1, 127.1, 136.2

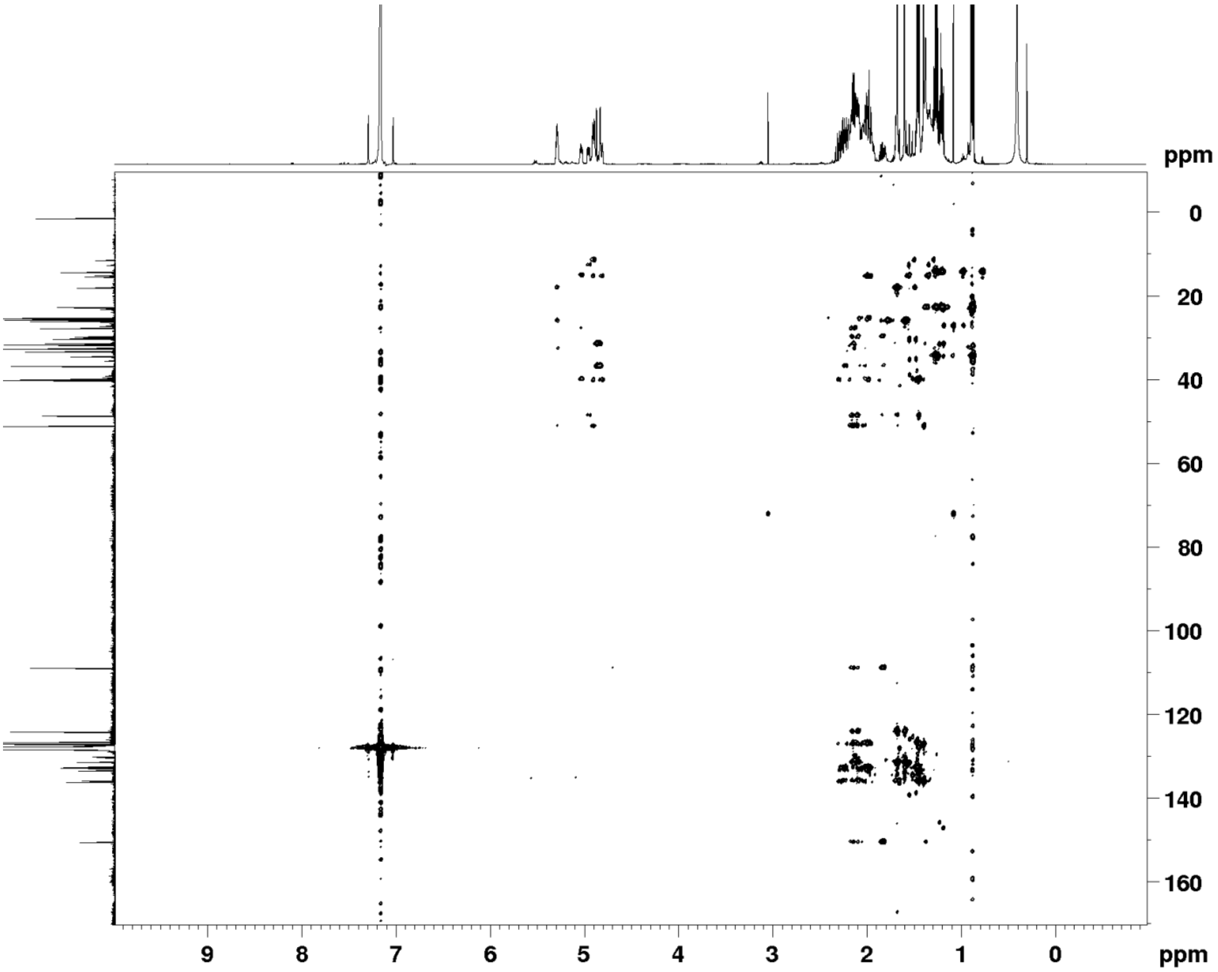

Figure S34. ^{1}H-^{13}C HMBC NMR spectrum of compound mixture **18a** and **18b** in C_6D_6.

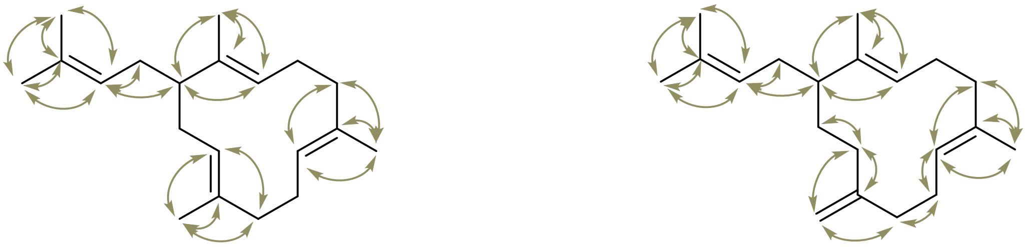

Figure S35. Key ^{1}H-^{13}C HMBC NMR correlations of **18a** and **18b** as indicated by arrows.

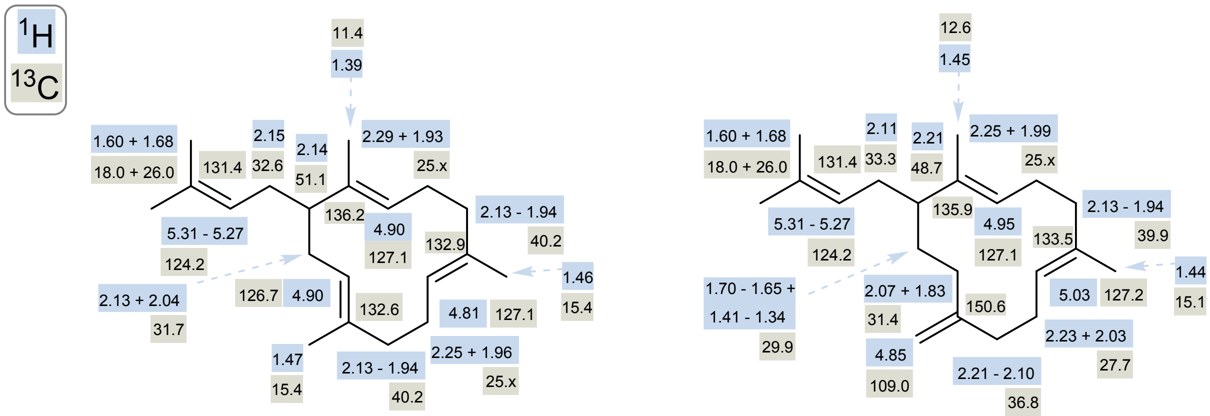

Figure S36. Full assignment of NMR spectra of **18a** and **18b**.

Important elements of the structure elucidation of **18a** and **18b**:

A correlation between a proton at δ= 1.46 ppm (methyl group C-8) and carbon atoms at δ= 40.2 ppm (C-9) and δ= 132.9 ppm (C-8) in the HMBC. This is a typical pattern for trisubstituted olefins.

The constitutional neighborship of C-4 and C-5 in **18b** was determined through correlations between the proton at δ= 4.85 ppm (C-4) and the carbon atom at δ= 36.8 ppm (C-5). The chemical shift at δ= 150.6 ppm (C-4) is indicative for 1,1-disubstituted alkenes. The proton at δ= 1.44 ppm (methyl group at C-8) shows a correlation with the carbon atoms at δ= 39.9 ppm (C-9) and δ= 133.5 ppm (C-8) in the HMBC spectrum. This is a typical pattern for trisubstituted olefins in terpenes. The chemical shift of protons at δ= 14.5 ppm (methyl group C-12) correlates with the carbon atoms at δ= 135.9 ppm (C-12) and δ= 48.7 ppm (C-1) in the HMBC spectrum, which allows to link C-12 with C-1. Finally, the proton at δ= 4.95 ppm correlates with the carbon atom at δ= 48.7 ppm in the HMBC spectrum. Furthermore the H,H-COSY spectrum reveals a correlation between H-1 and H-2.

2. Copies of NMR spectra

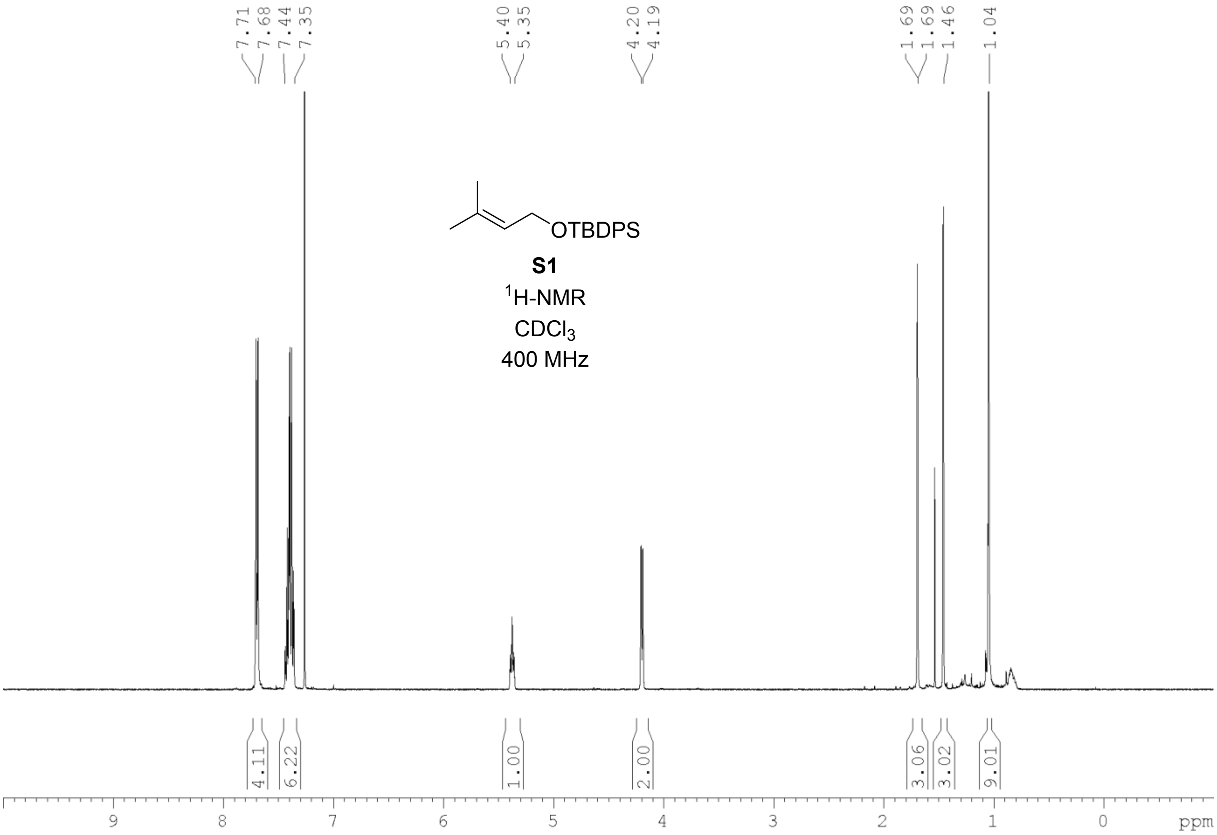

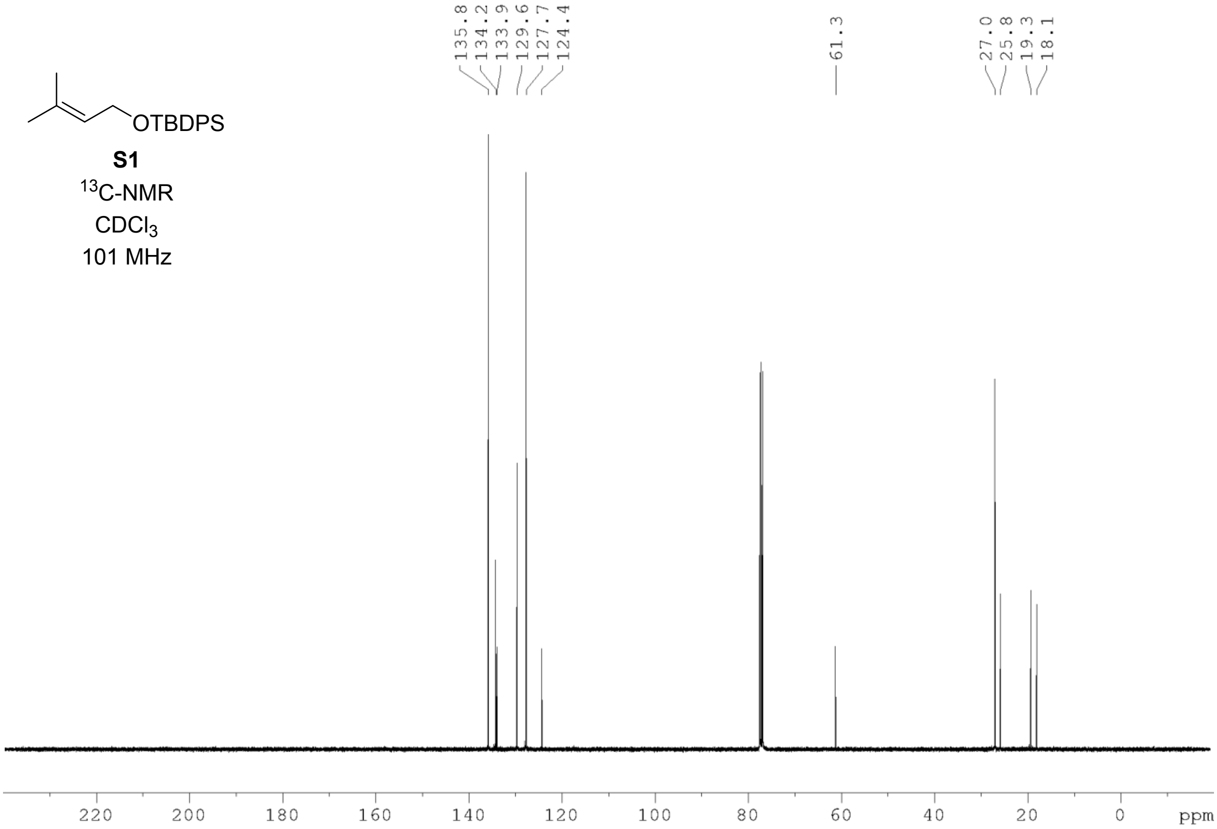

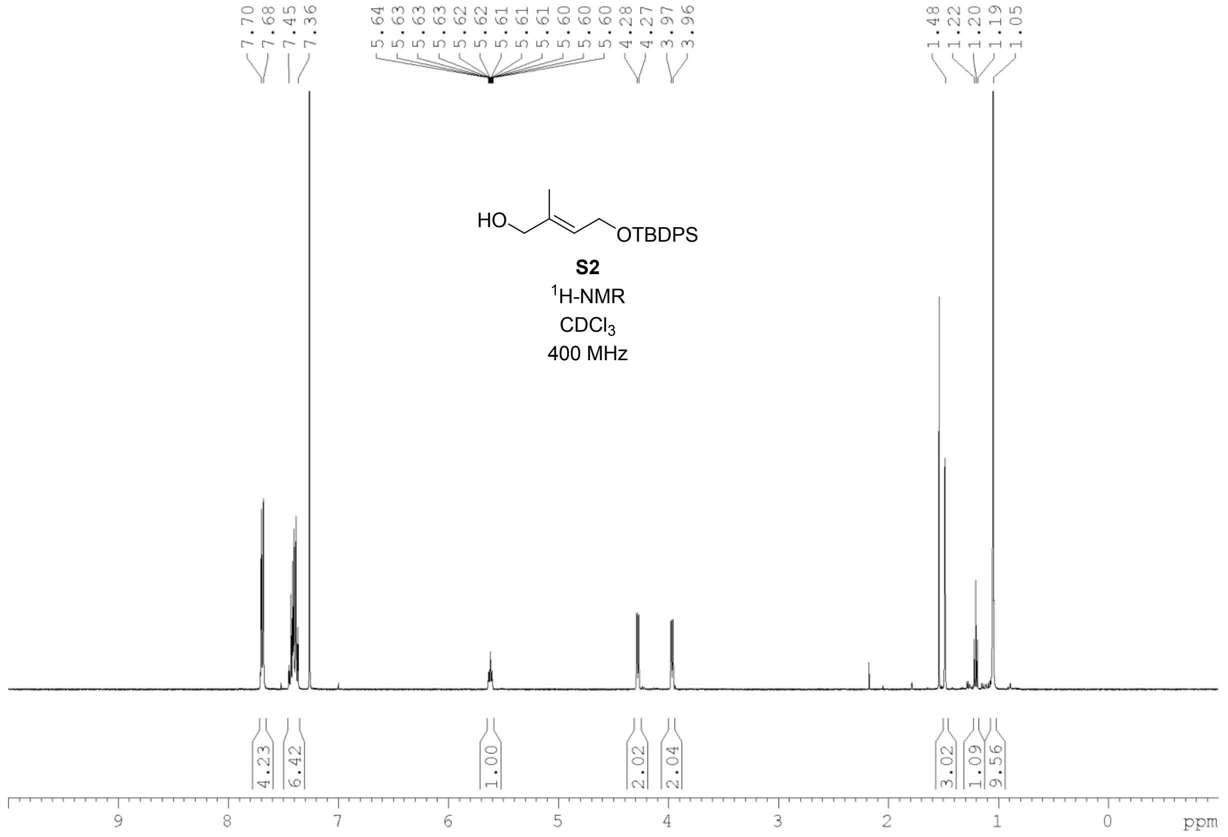
7.70
7.68
7.45
7.36
5.64
5.63
5.63
5.63
5.62
5.62
5.61
5.61
5.61
5.60
5.60
5.60
4.28
4.27
3.97
3.96
1.48
1.22
1.20
1.19
1.05
HO
OTBDPS
S2
1H-NMR
CDCl3
400 MHz
4.23
6.42
1.00
2.02
2.04
3.02
1.09
9.56
9
8
7
6
5
4
3
2
1
0
ppm

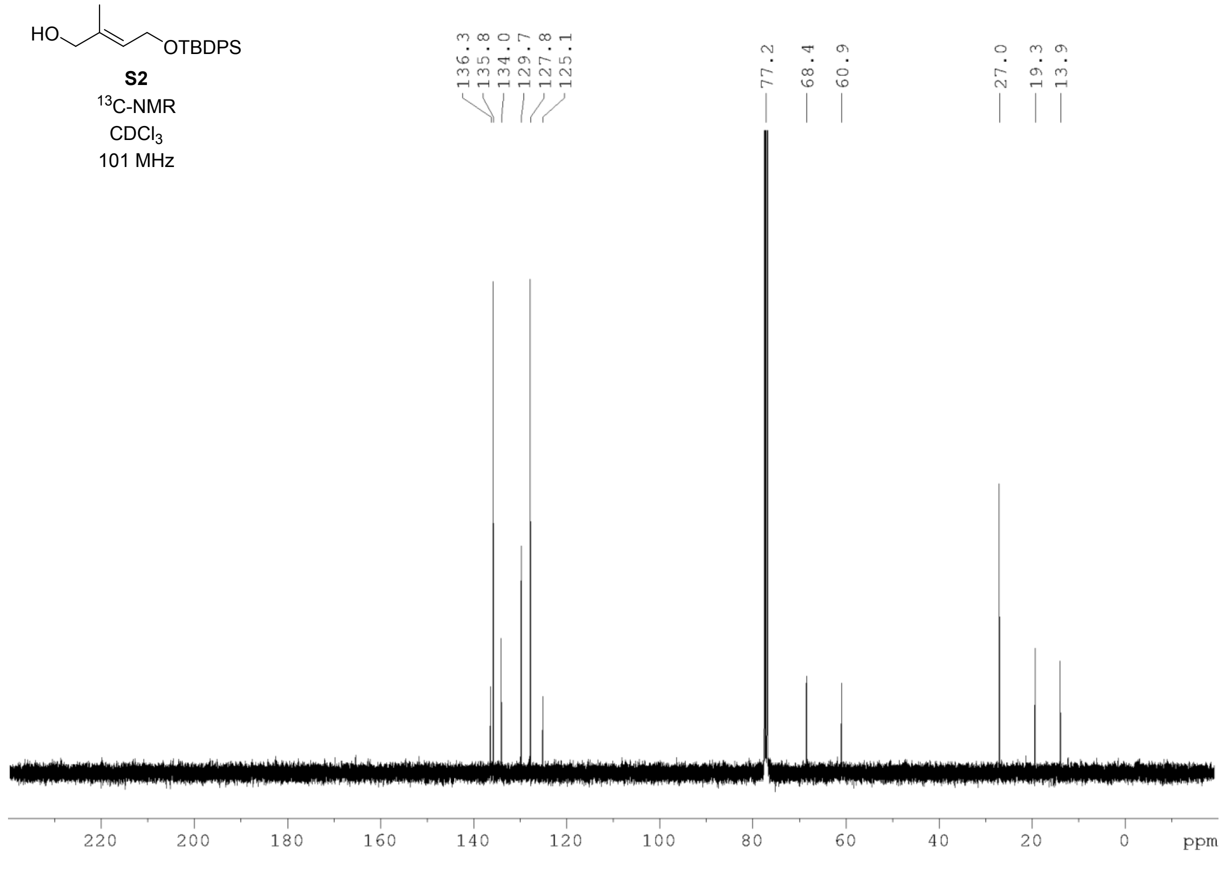
HO
OTBDPS
S2
13C-NMR
CDCl3
101 MHz
136.3
135.8
134.0
129.7
127.8
125.1
77.2
68.4
60.9
27.0
19.3
13.9
220
200
180
160
140
120
100
80
60
40
20
0
ppm

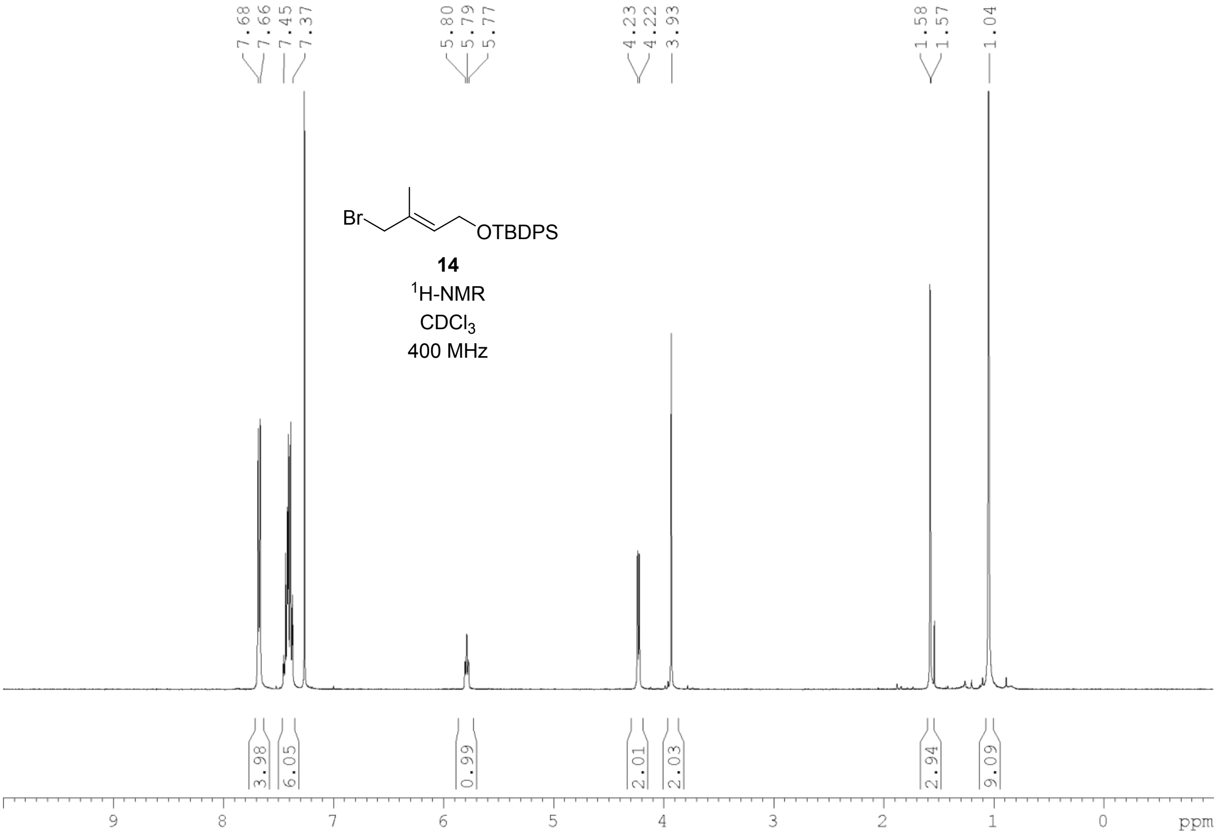
7.68
7.66
7.45
7.37
5.80
5.79
5.77
4.23
4.22
3.93
1.58
1.57
1.04
Br
OTBDPS
14
1H-NMR
CDCl3
400 MHz
3.98
6.05
0.99
2.01
2.03
2.94
9.09
9
8
7
6
5
4
3
2
1
0
ppm

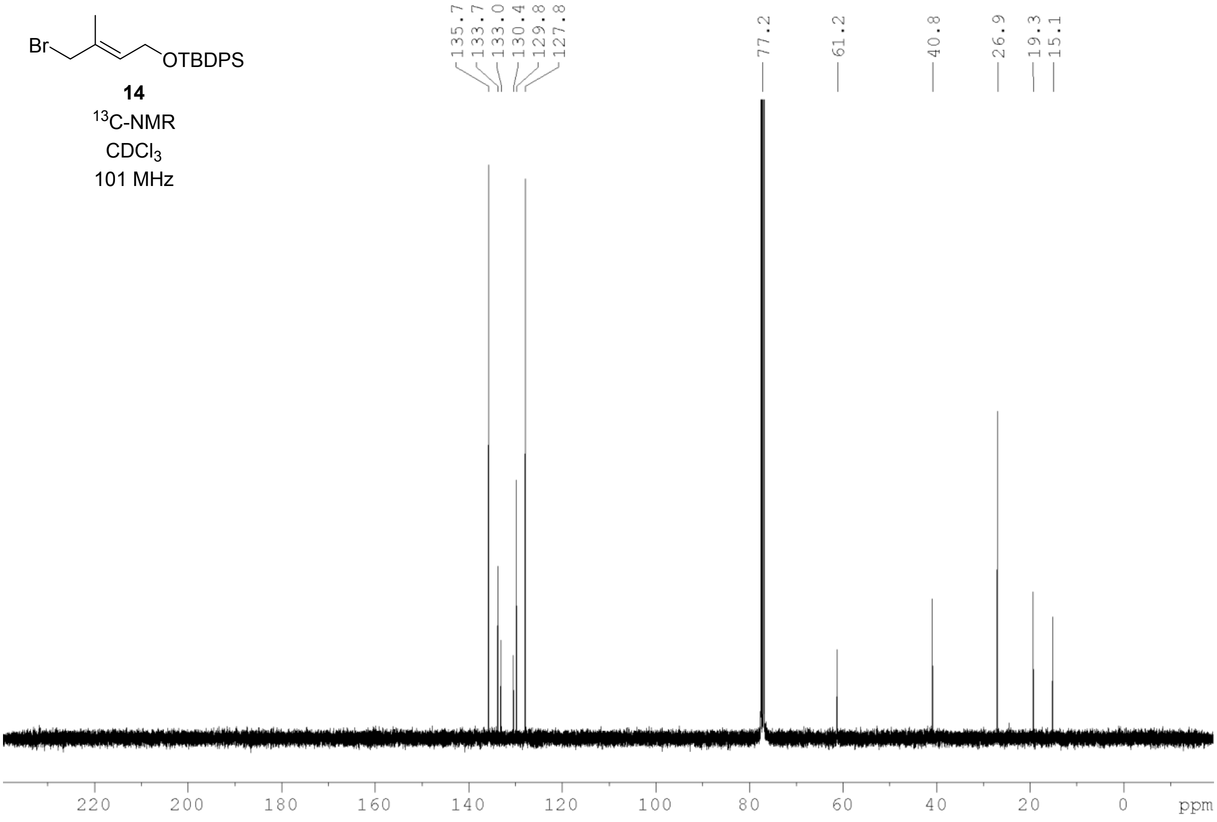
Br
OTBDPS
14
13C-NMR
CDCl3
101 MHz
135.7
133.7
133.0
130.4
129.8
127.8
77.2
61.2
40.8
26.9
19.3
15.1
220
200
180
160
140
120
100
80
60
40
20
0
ppm

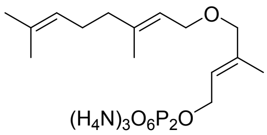

6
^{1}H-NMR
D_2O
400 MHz

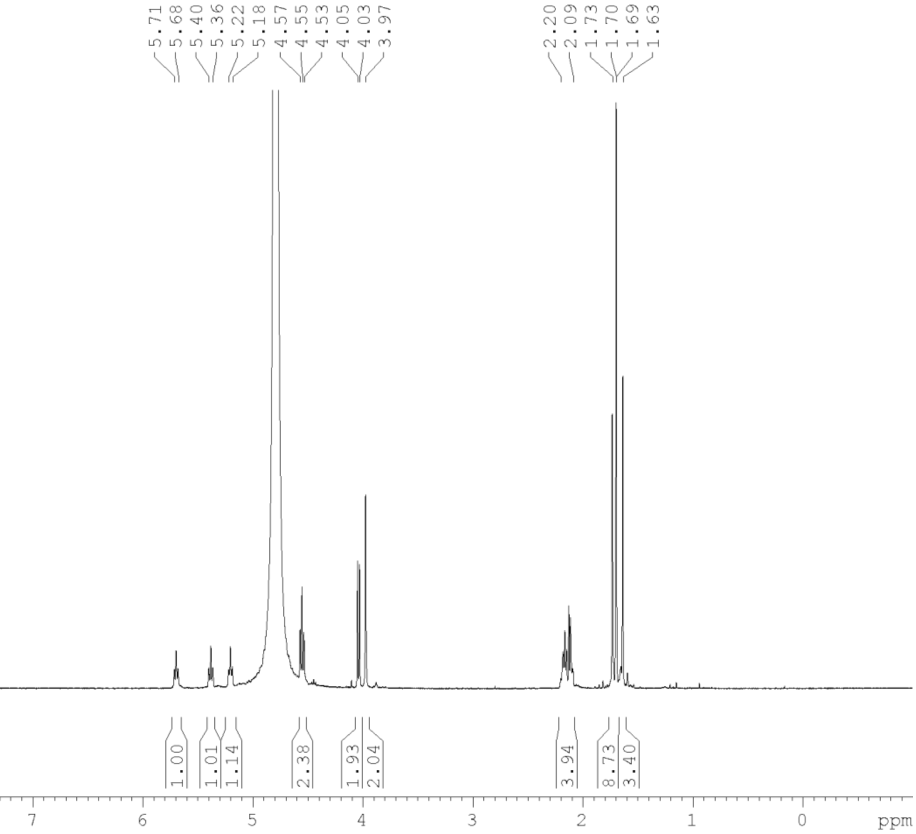

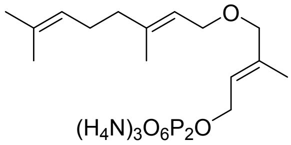

6
^{13}C-NMR
D_2O
101 MHz

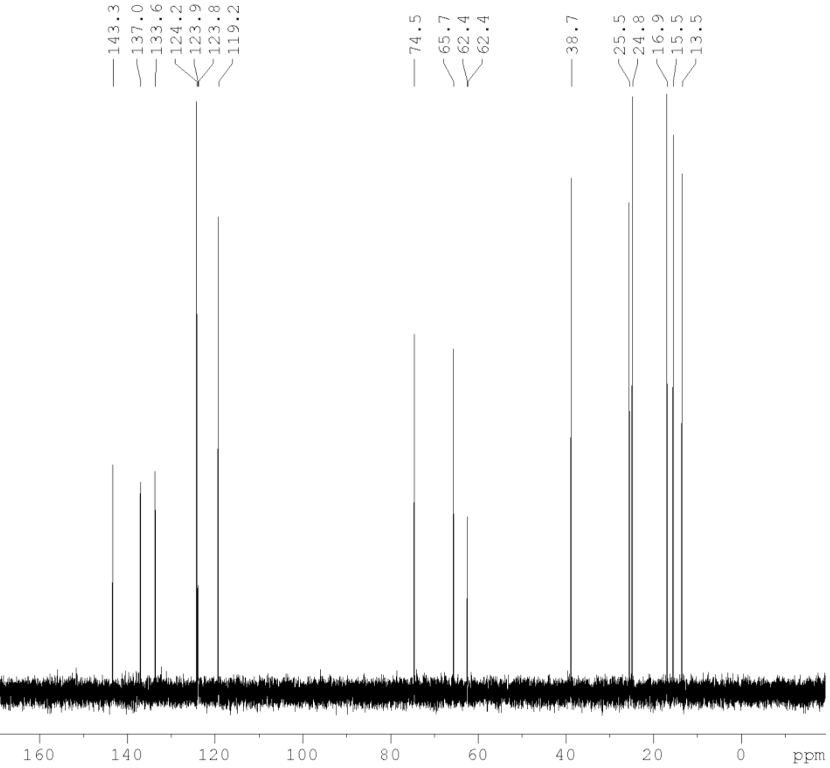

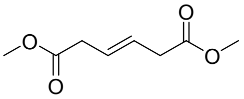

10
^{1}H-NMR
$CDCl_3$
400 MHz

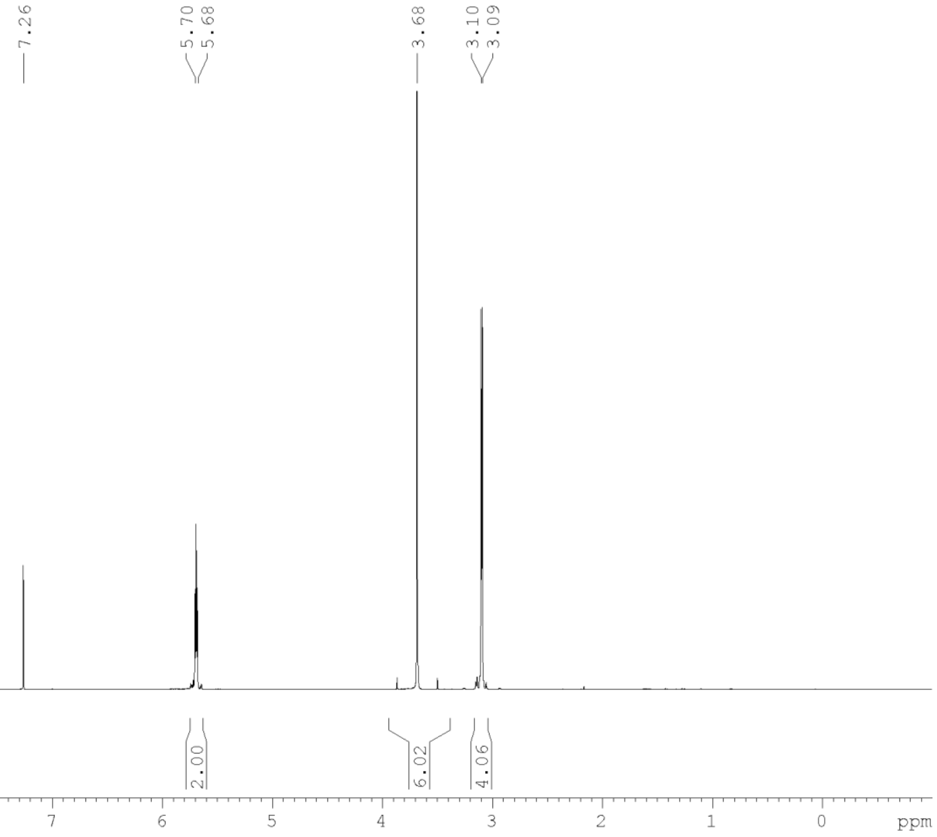

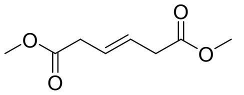

10
^{13}C-NMR
$CDCl_3$
101 MHz

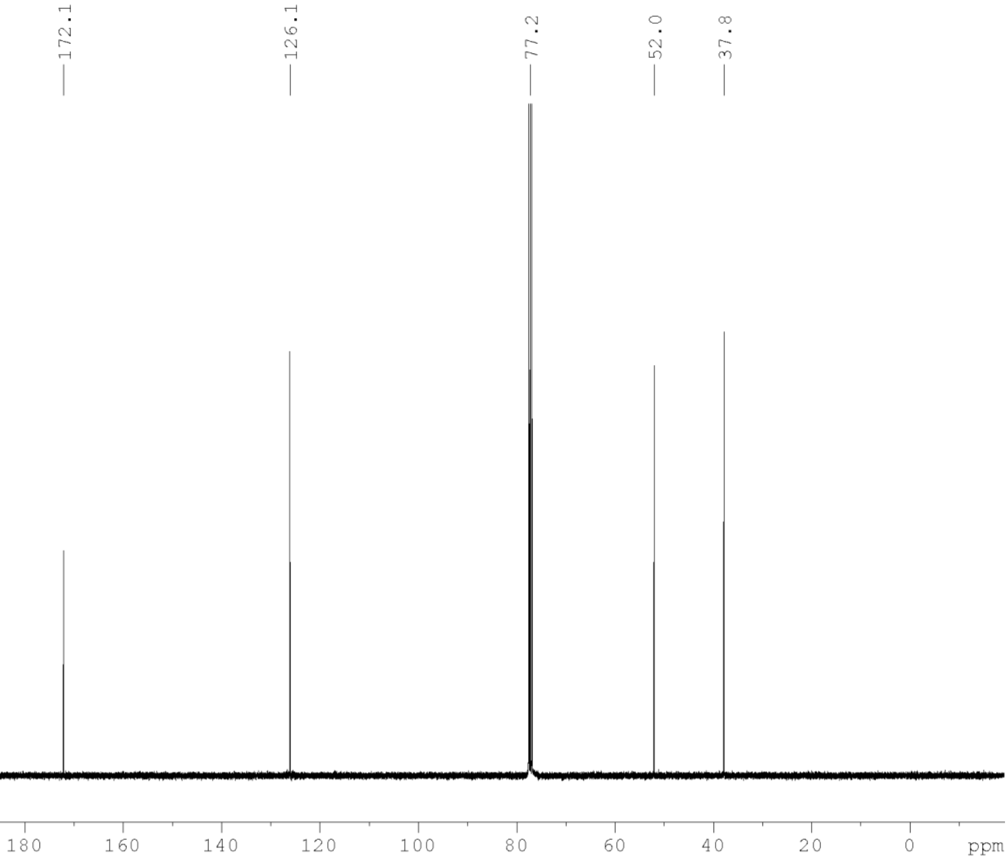

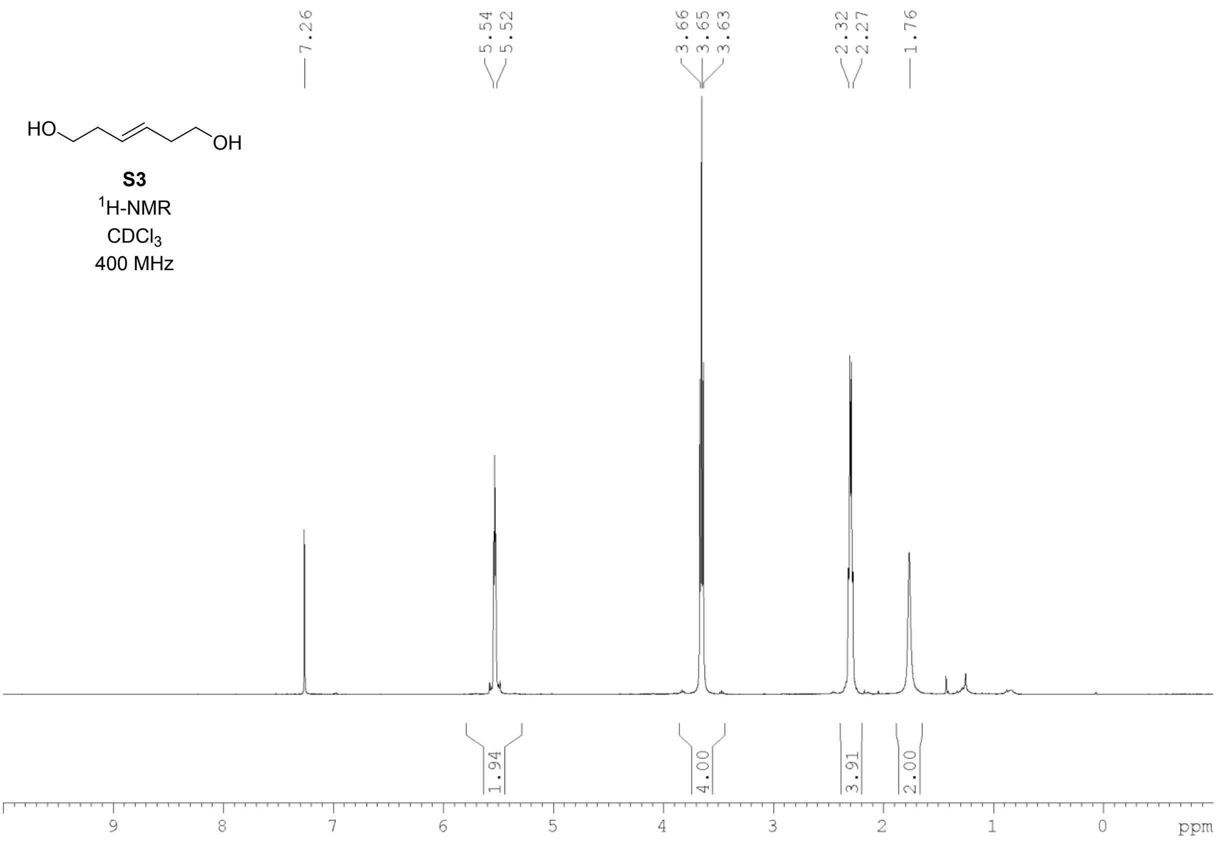

HO
OH
S3
^{1}H-NMR
$CDCl_3$
400 MHz
7.26
5.54
5.52
3.66
3.65
3.63
2.32
2.27
1.76
1.94
4.00
3.91
2.00
9
8
7
6
5
4
3
2
1
0
ppm

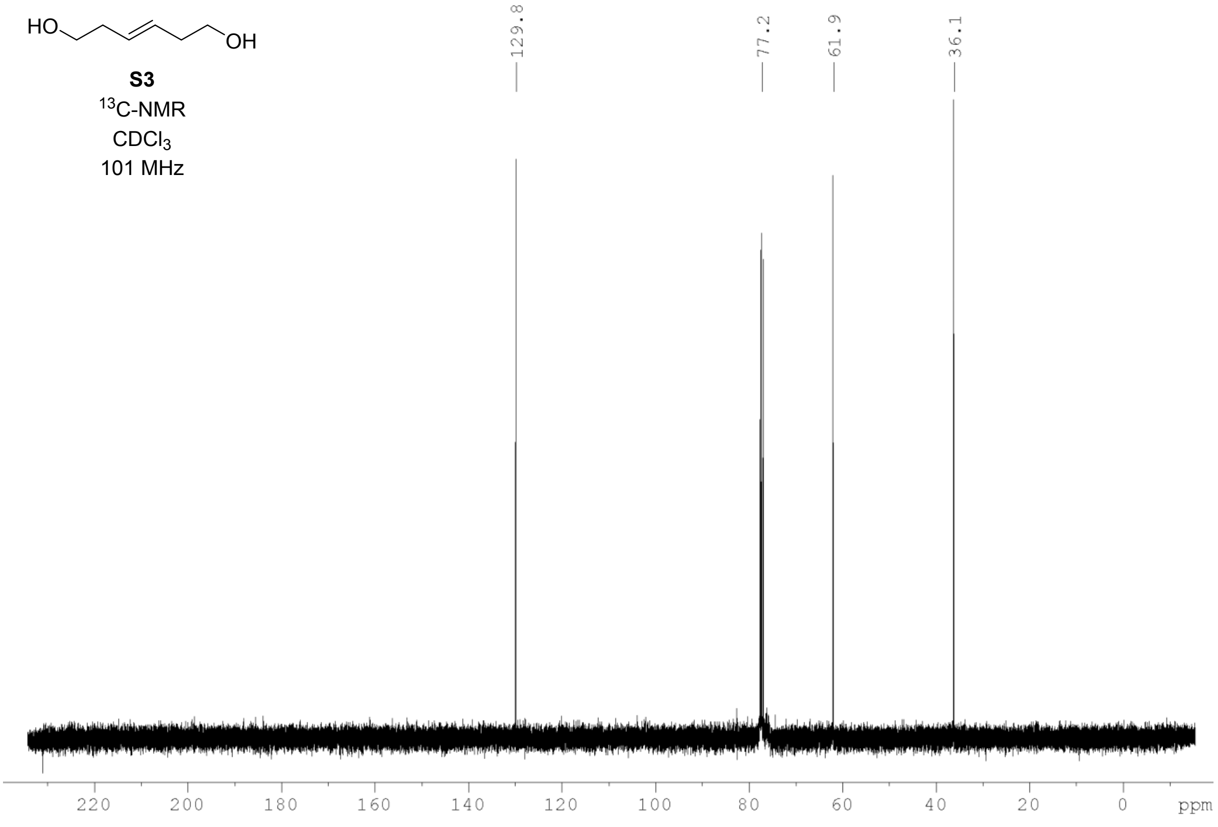

HO
OH
S3
^{13}C-NMR
$CDCl_3$
101 MHz
129.8
77.2
61.9
36.1
220
200
180
160
140
120
100
80
60
40
20
0
ppm

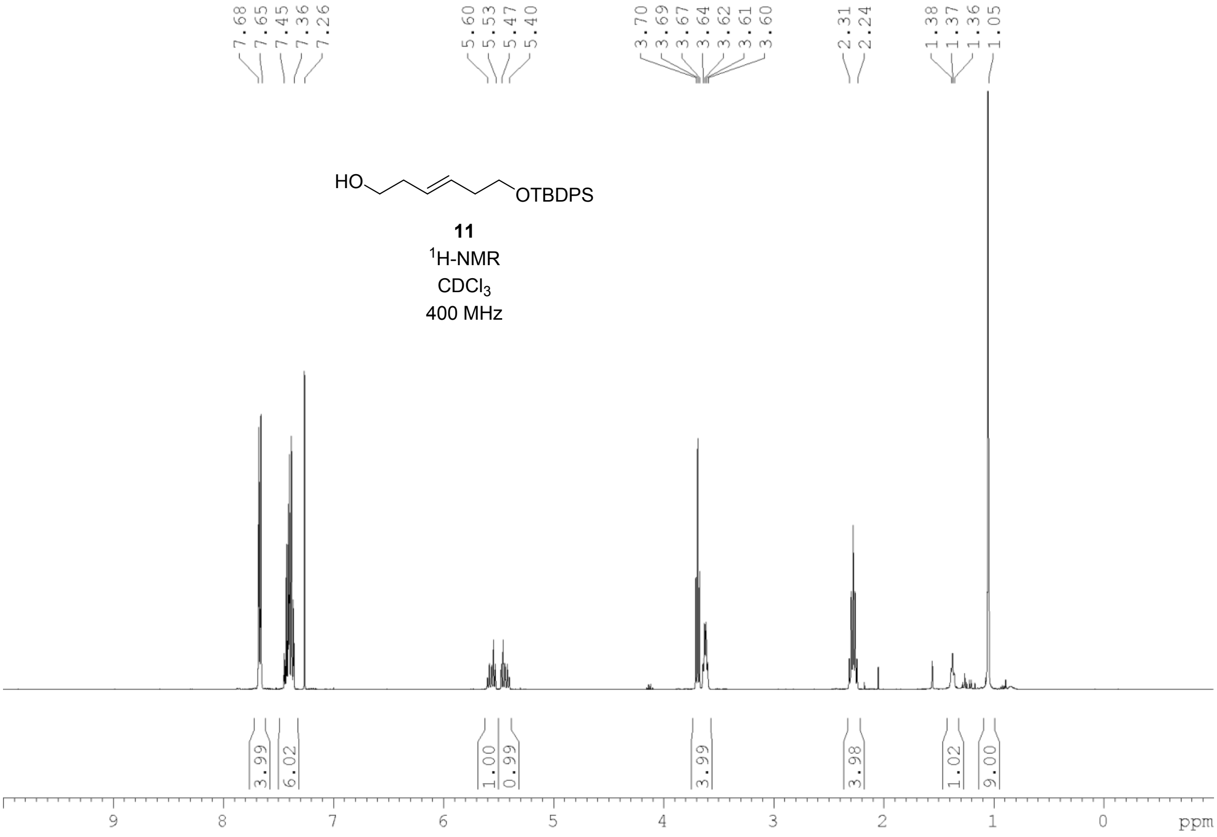
7.68
7.65
7.45
7.36
7.26
5.60
5.53
5.47
5.40
3.70
3.69
3.67
3.64
3.62
3.61
3.60
2.31
2.24
1.38
1.37
1.36
1.05
HO
OTBDPS
11
^{1}H-NMR
$CDCl_3$
400 MHz
3.99
6.02
1.00
0.99
3.99
3.98
1.02
9.00
9
8
7
6
5
4
3
2
1
0
ppm

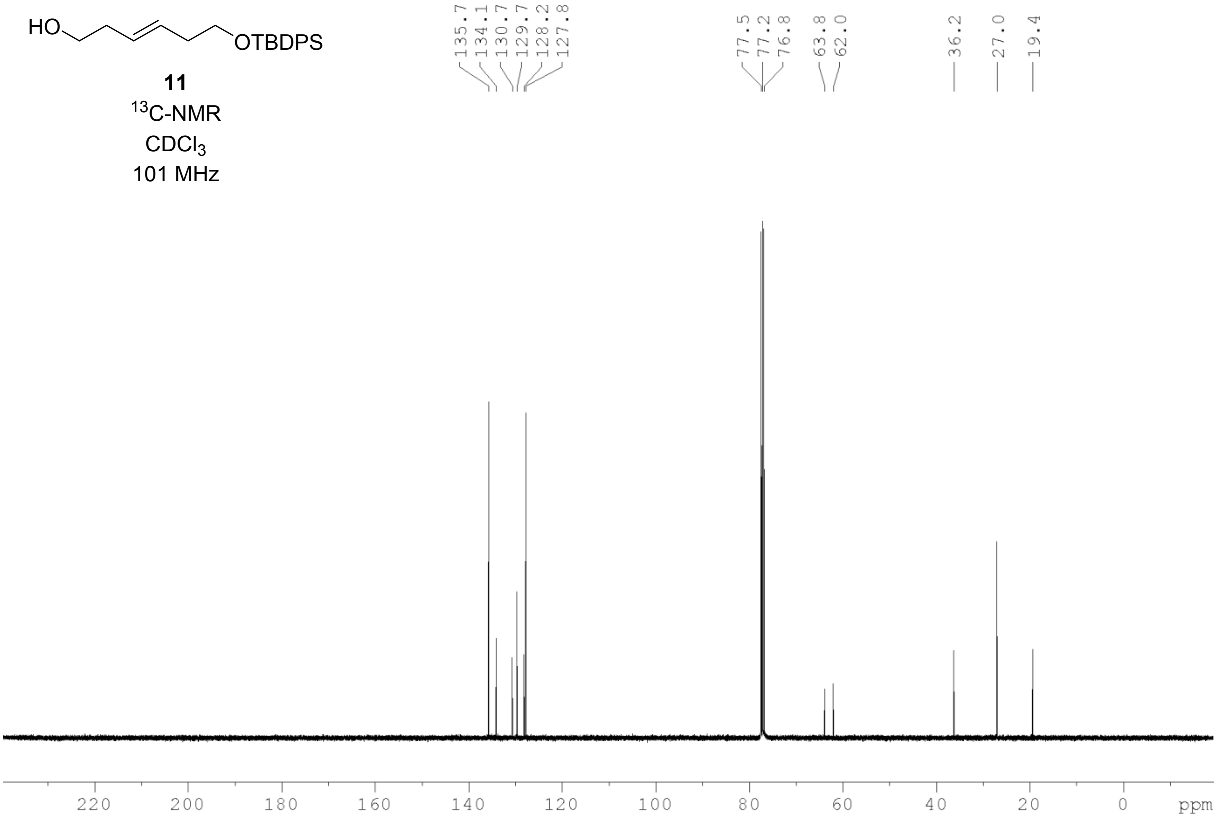
HO
OTBDPS
11
^{13}C-NMR
$CDCl_3$
101 MHz
135.7
134.1
130.7
129.7
128.2
127.8
77.5
77.2
76.8
63.8
62.0
36.2
27.0
19.4
220
200
180
160
140
120
100
80
60
40
20
0
ppm

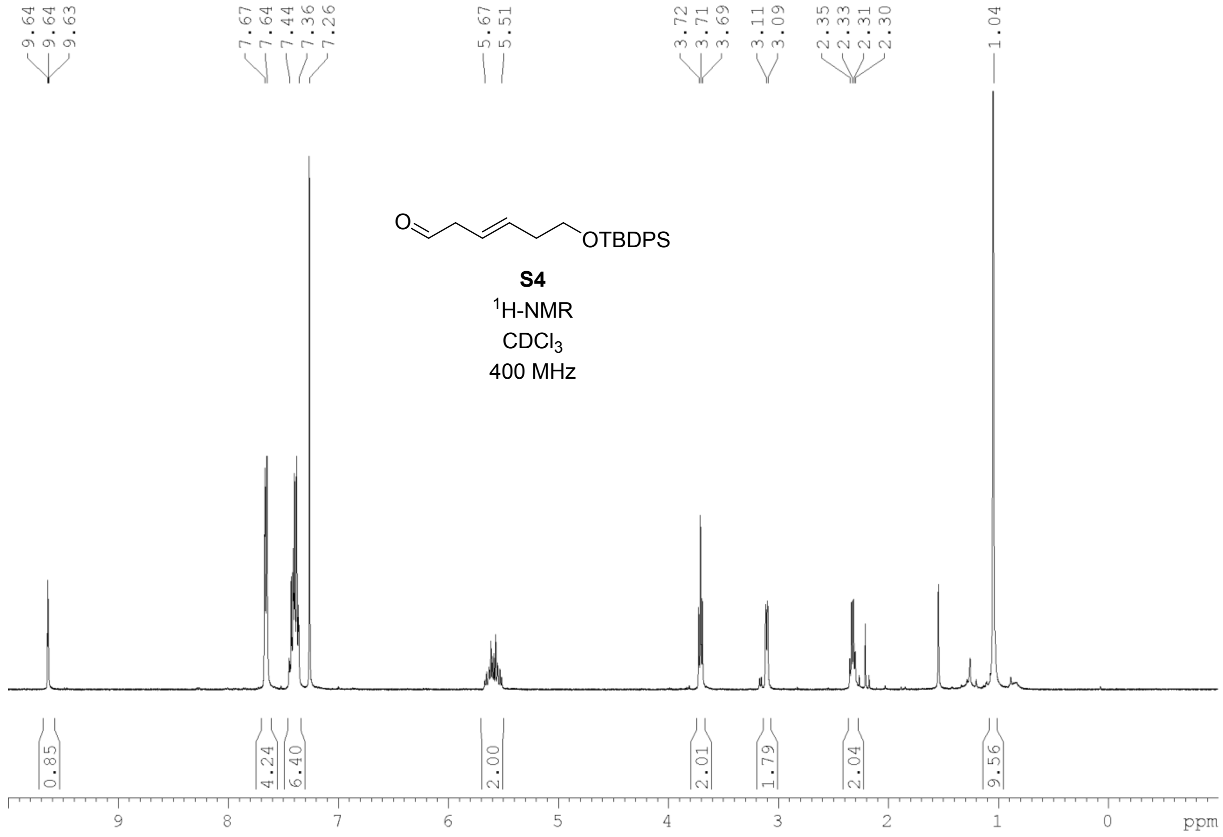
OTBDPS
S4
1H-NMR
CDCl3
400 MHz
9.64 9.64 9.63
7.67 7.64 7.44 7.36 7.26
5.67 5.51
3.72 3.71 3.69
3.11 3.09
2.35 2.33 2.31 2.30
1.04
0.85
4.24
6.40
2.00
2.01
1.79
2.04
9.56
9
8
7
6
5
4
3
2
1
0
ppm

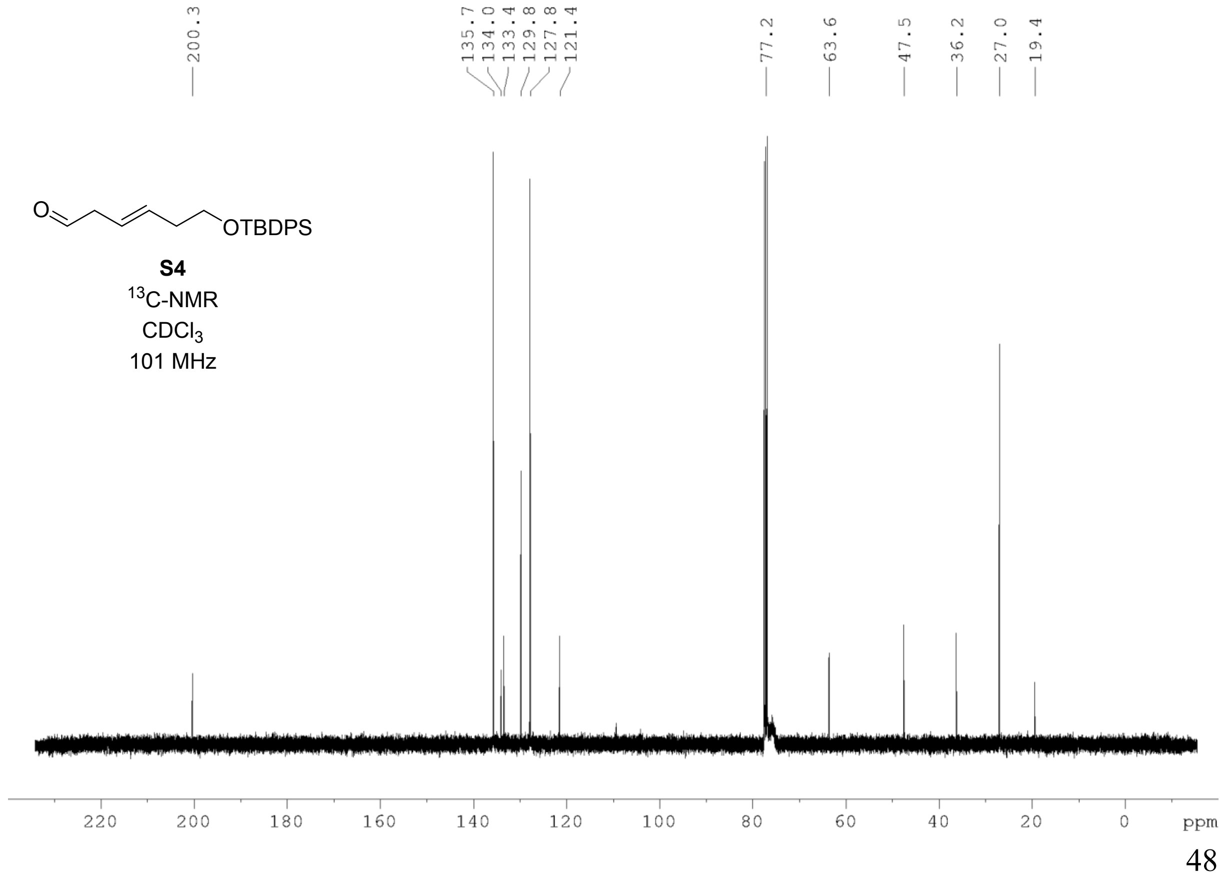
OTBDPS
S4
13C-NMR
CDCl3
101 MHz
200.3
135.7 134.0 133.4 129.8 127.8 121.4
77.2
63.6
47.5
36.2
27.0
19.4
220
200
180
160
140
120
100
80
60
40
20
0
ppm

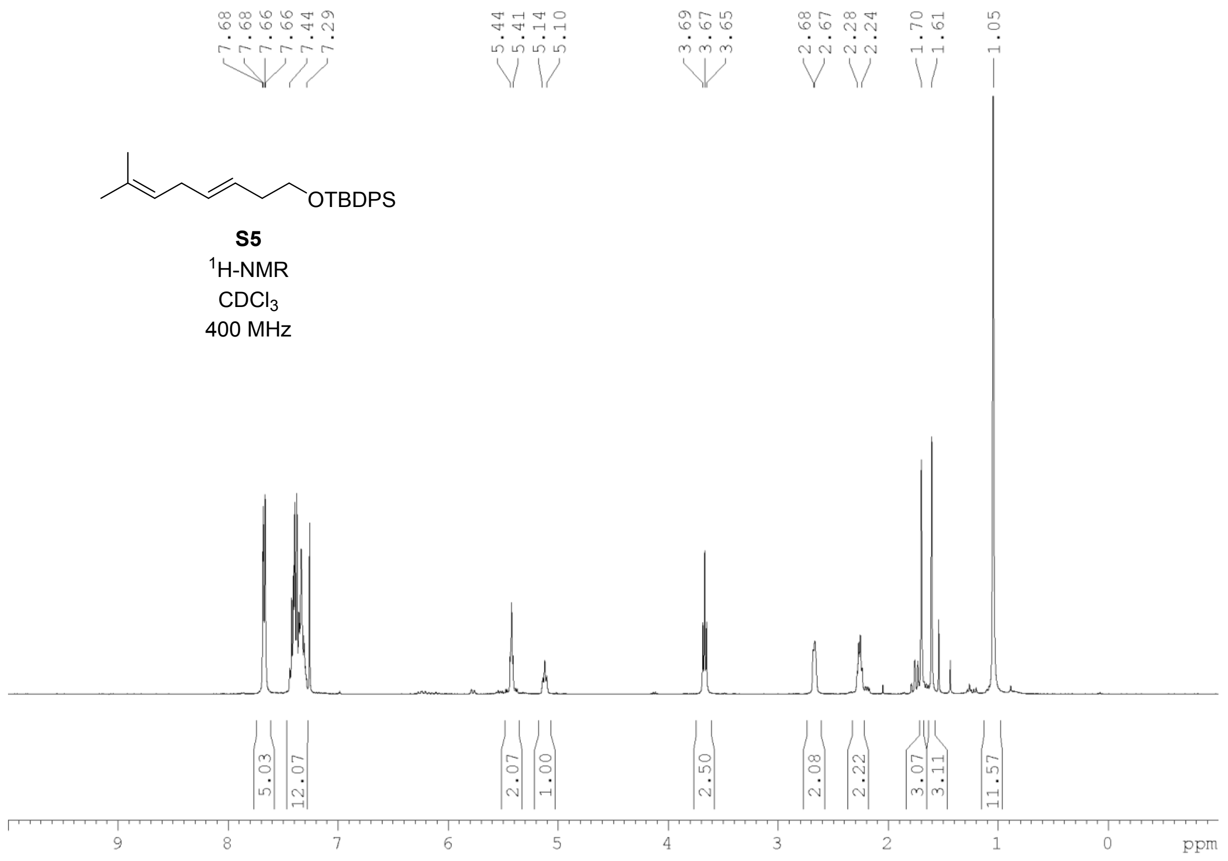
OTBDPS
S5
1H-NMR
CDCl3
400 MHz
7.68 7.68 7.66 7.66 7.44 7.29
5.44 5.41 5.14 5.10
3.69 3.67 3.65
2.68 2.67 2.28 2.24
1.70 1.61
1.05
5.03 12.07 2.07 1.00 2.50 2.08 2.22 3.07 3.11 11.57
9 8 7 6 5 4 3 2 1 0 ppm

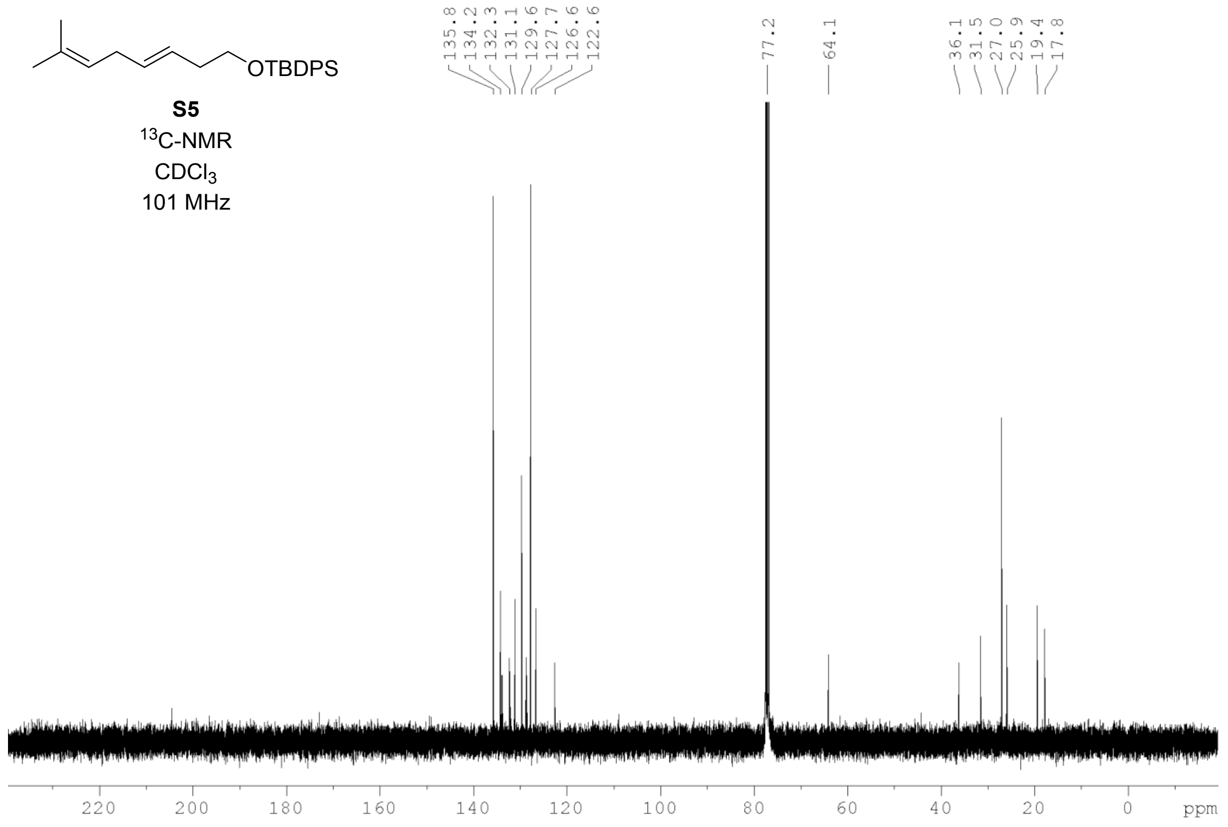
OTBDPS
S5
13C-NMR
CDCl3
101 MHz
135.8 134.2 132.3 131.1 129.6 127.7 126.6 122.6
77.2
64.1
36.1 31.5 27.0 25.9 19.4 17.8
220 200 180 160 140 120 100 80 60 40 20 0 ppm

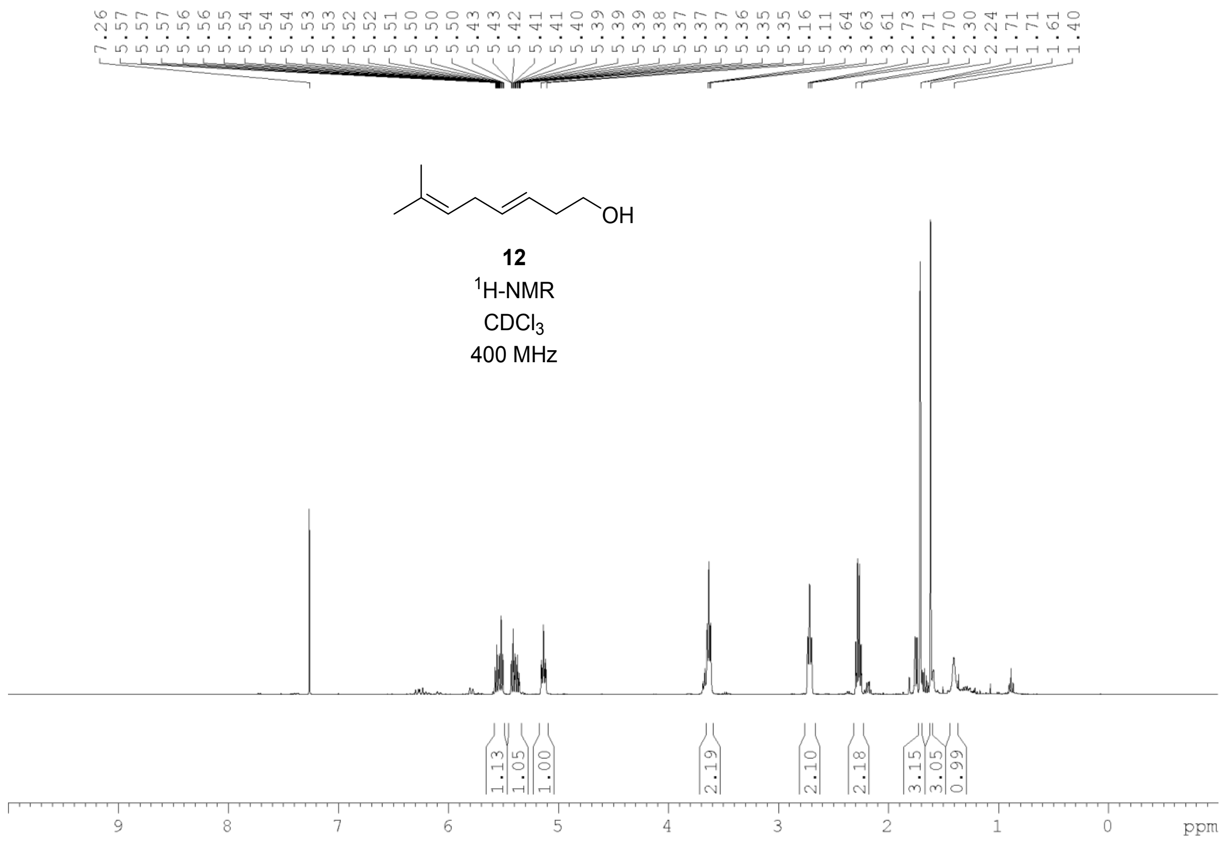
7.26 5.57 5.57 5.57 5.56 5.56 5.55 5.54 5.54 5.54 5.53 5.53 5.52 5.52 5.51 5.50 5.50 5.50 5.43 5.43 5.42 5.41 5.41 5.40 5.39 5.39 5.39 5.38 5.37 5.37 5.37 5.36 5.35 5.35 5.16 5.11 3.64 3.63 3.61 2.73 2.71 2.70 2.30 2.24 1.71 1.71 1.61 1.40
OH
12
1H-NMR
CDCl3
400 MHz
1.13 1.05 1.00 2.19 2.10 2.18 3.15 3.05 0.99
9 8 7 6 5 4 3 2 1 0 ppm

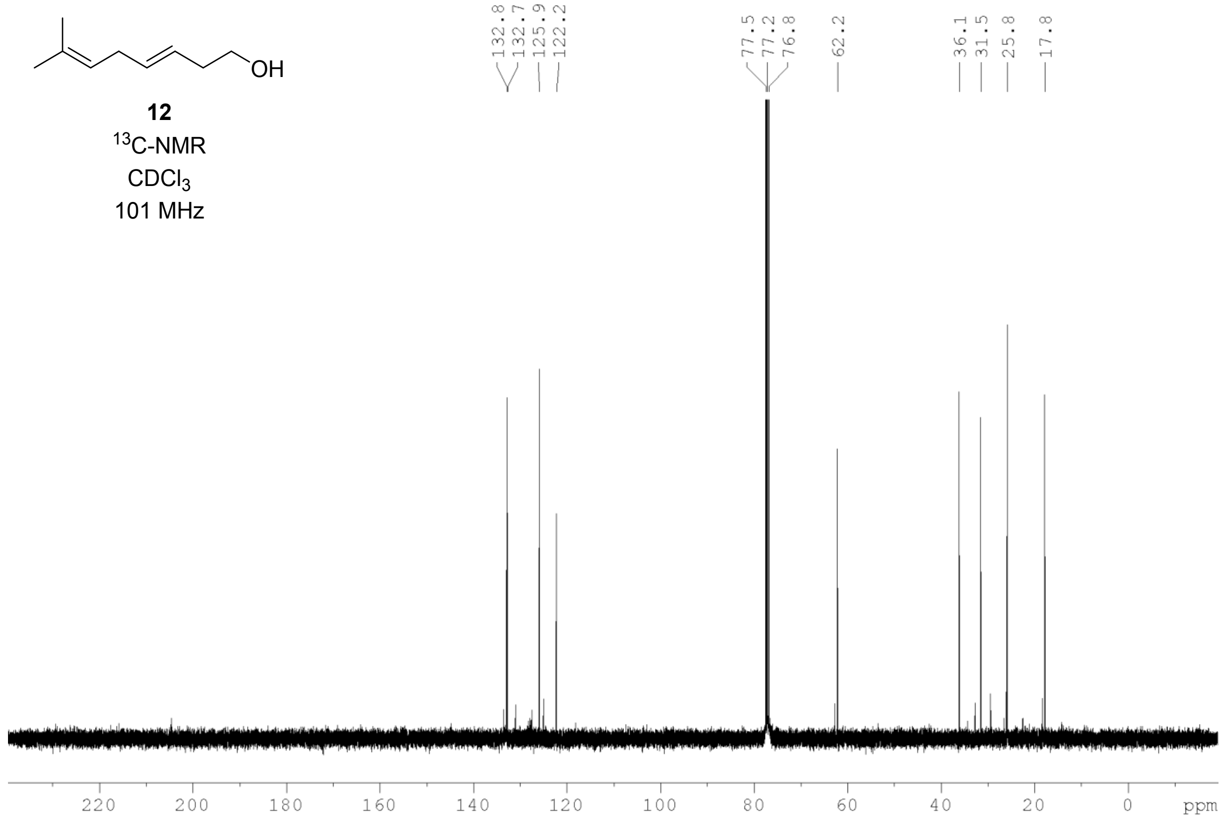
OH
12
13C-NMR
CDCl3
101 MHz
132.8 132.7 125.9 122.2 77.5 77.2 76.8 62.2 36.1 31.5 25.8 17.8
220 200 180 160 140 120 100 80 60 40 20 0 ppm

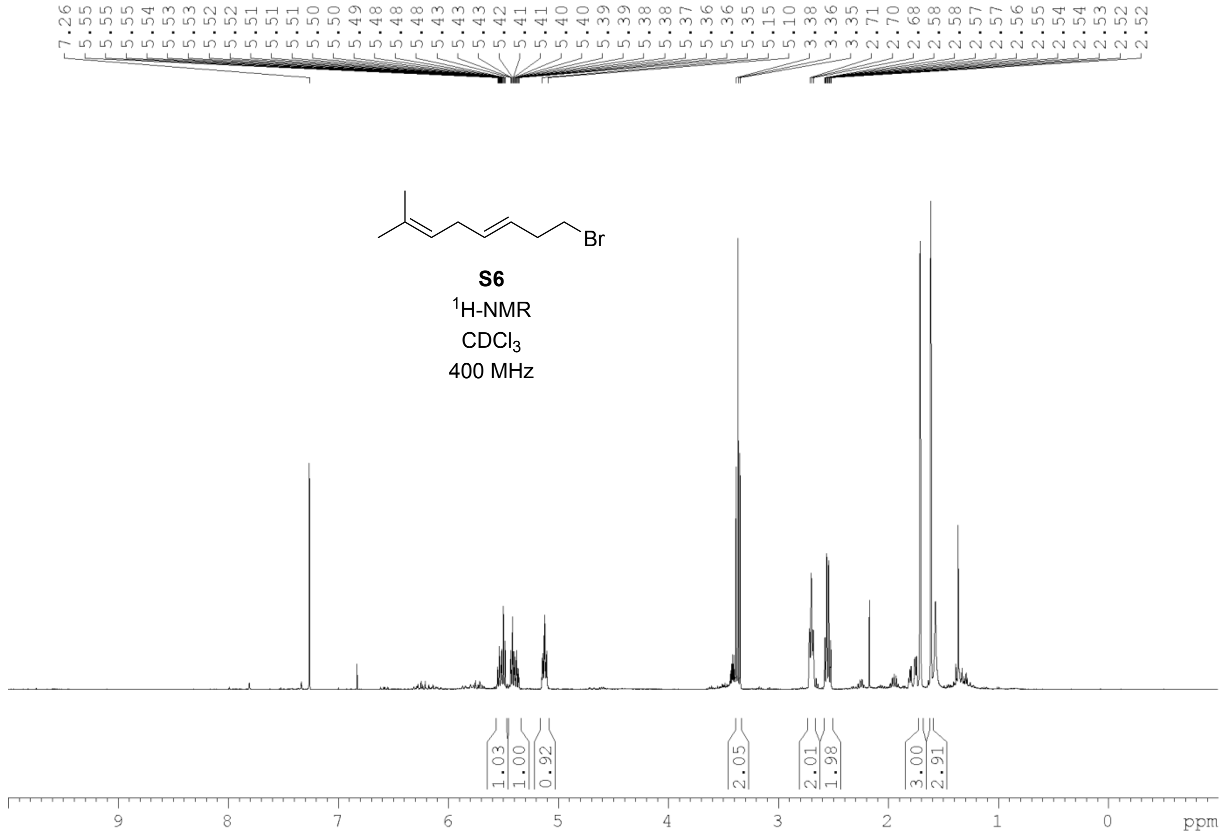
7.26 5.55 5.55 5.54 5.53 5.53 5.52 5.52 5.51 5.51 5.51 5.50 5.50 5.49 5.48 5.48 5.48 5.43 5.43 5.43 5.42 5.41 5.41 5.40 5.40 5.39 5.39 5.38 5.38 5.37 5.36 5.36 5.35 5.15 5.10 3.38 3.36 3.35 2.71 2.70 2.68 2.58 2.58 2.57 2.57 2.56 2.55 2.54 2.54 2.53 2.52 2.52
Br
S6
1H-NMR
CDCl3
400 MHz
1.03 1.00 0.92 2.05 2.01 1.98 3.00 2.91
9 8 7 6 5 4 3 2 1 0 ppm

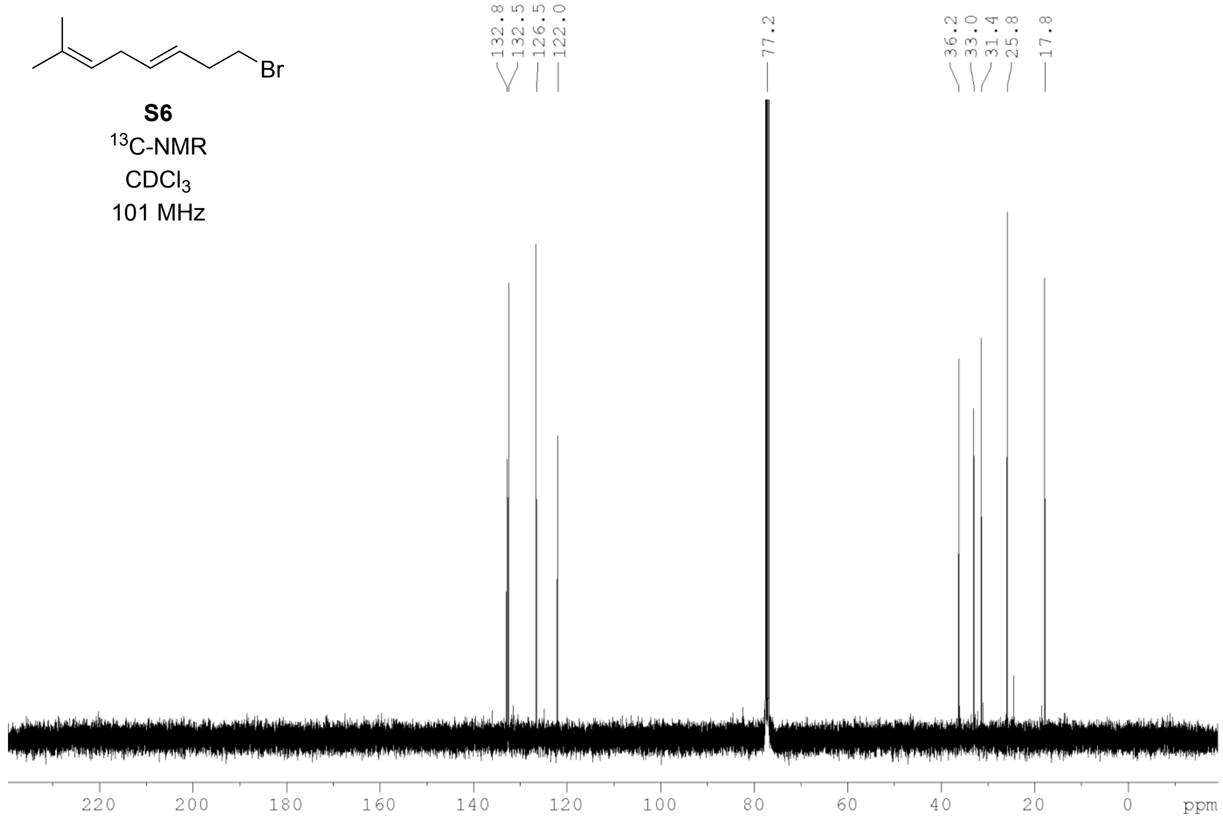
Br
S6
13C-NMR
CDCl3
101 MHz
132.8 132.5 126.5 122.0 77.2 36.2 33.0 31.4 25.8 17.8
220 200 180 160 140 120 100 80 60 40 20 0 ppm

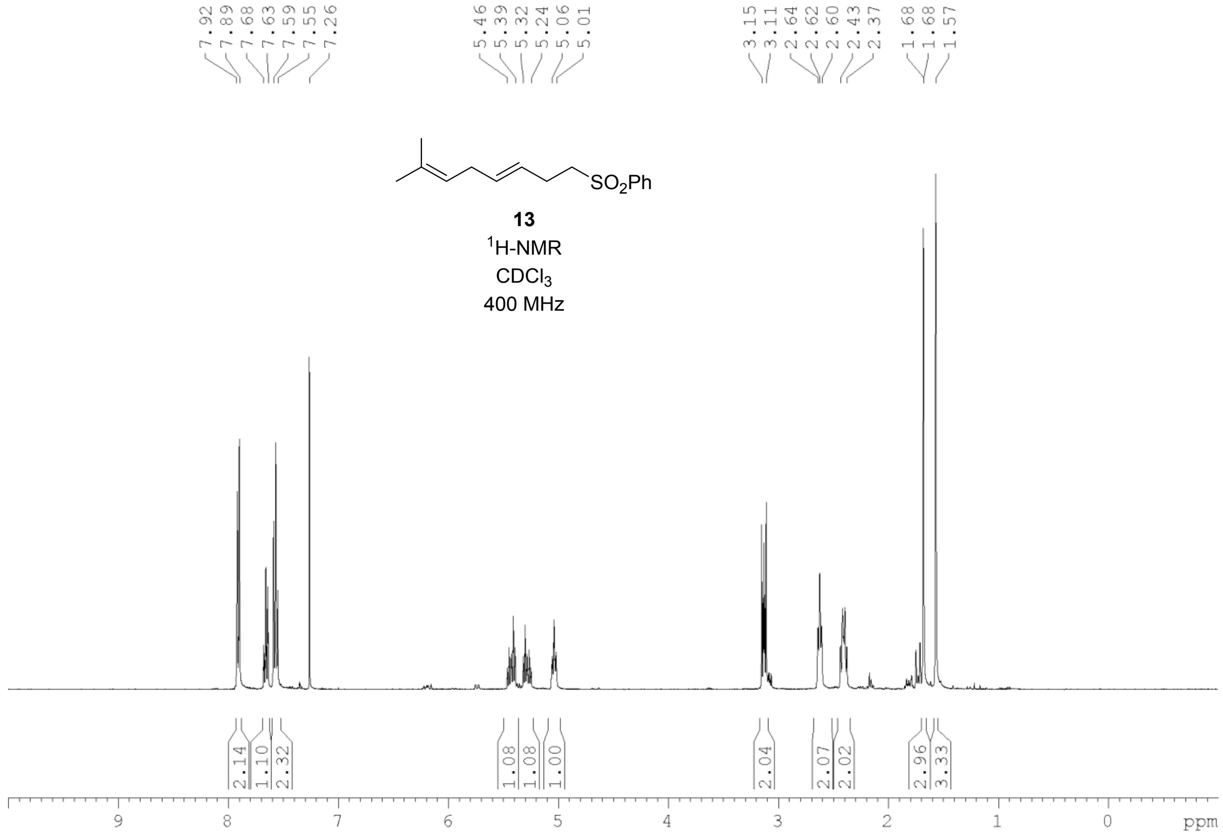
7.92
7.89
7.68
7.63
7.59
7.55
7.26
5.46
5.39
5.32
5.24
5.06
5.01
3.15
3.11
2.64
2.62
2.60
2.43
2.37
1.68
1.68
1.57
SO2Ph
13
1H-NMR
CDCl3
400 MHz
2.14
1.10
2.32
1.08
1.08
1.00
2.04
2.07
2.02
2.96
3.33
9 8 7 6 5 4 3 2 1 0 ppm

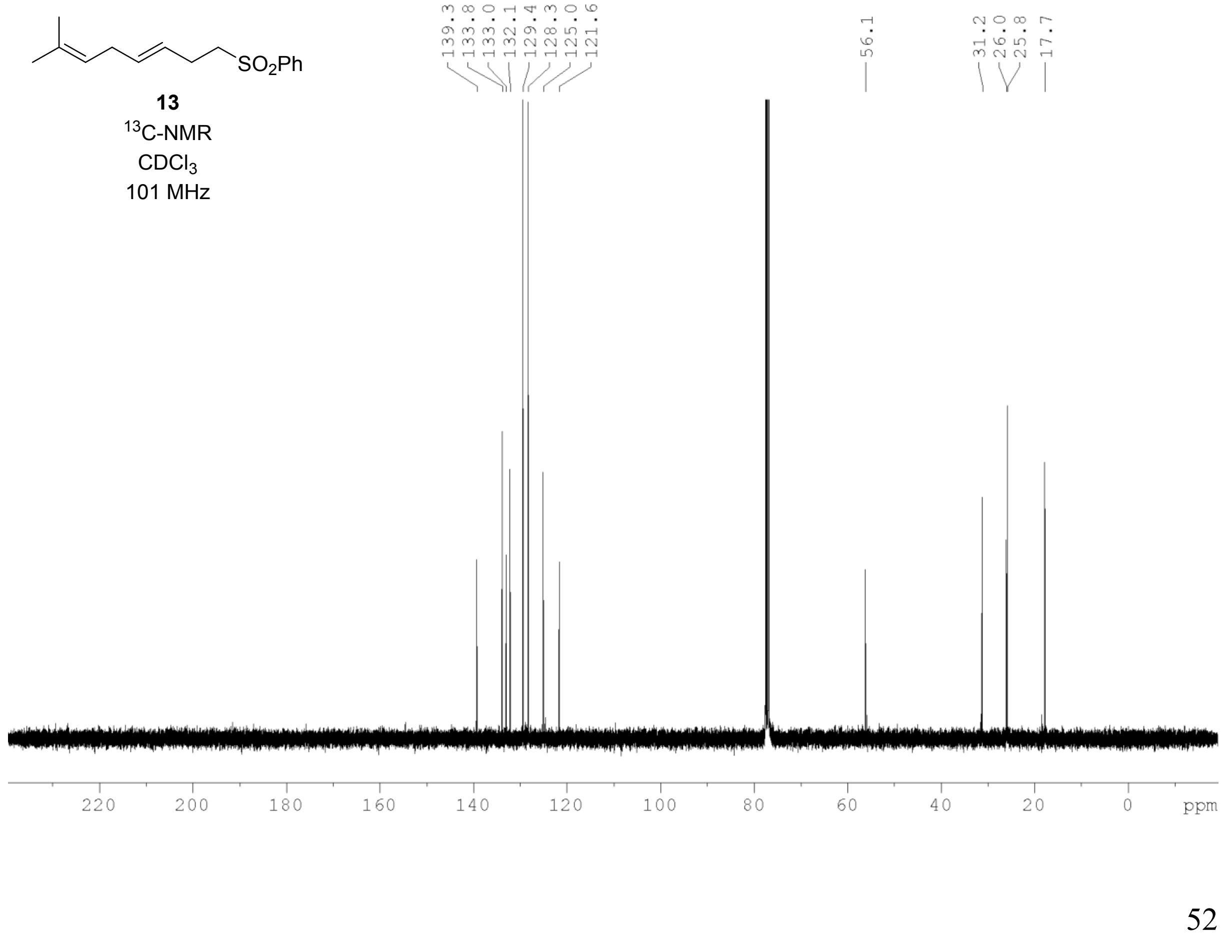
SO2Ph
13
13C-NMR
CDCl3
101 MHz
139.3
133.8
133.0
132.1
129.4
128.3
125.0
121.6
56.1
31.2
26.0
25.8
17.7
220 200 180 160 140 120 100 80 60 40 20 0 ppm

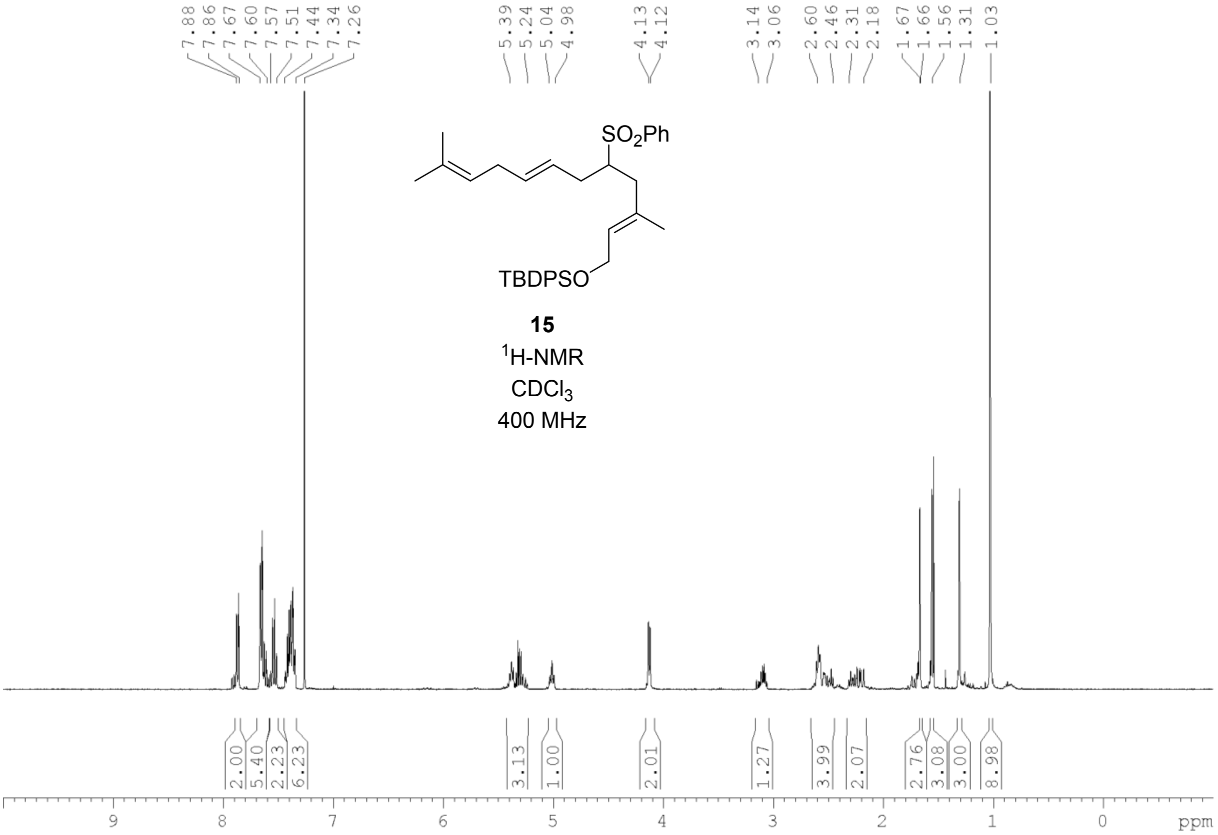
7.88 7.86 7.67 7.60 7.57 7.51 7.44 7.34 7.26
5.39 5.24 5.04 4.98
4.13 4.12
3.14 3.06
2.60 2.46 2.31 2.18
1.67 1.66 1.56 1.31 1.03
SO2Ph
TBDPSO
15
1H-NMR
CDCl3
400 MHz
2.00 5.40 2.23 6.23 3.13 1.00 2.01 1.27 3.99 2.07 2.76 3.08 3.00 8.98
9 8 7 6 5 4 3 2 1 0 ppm

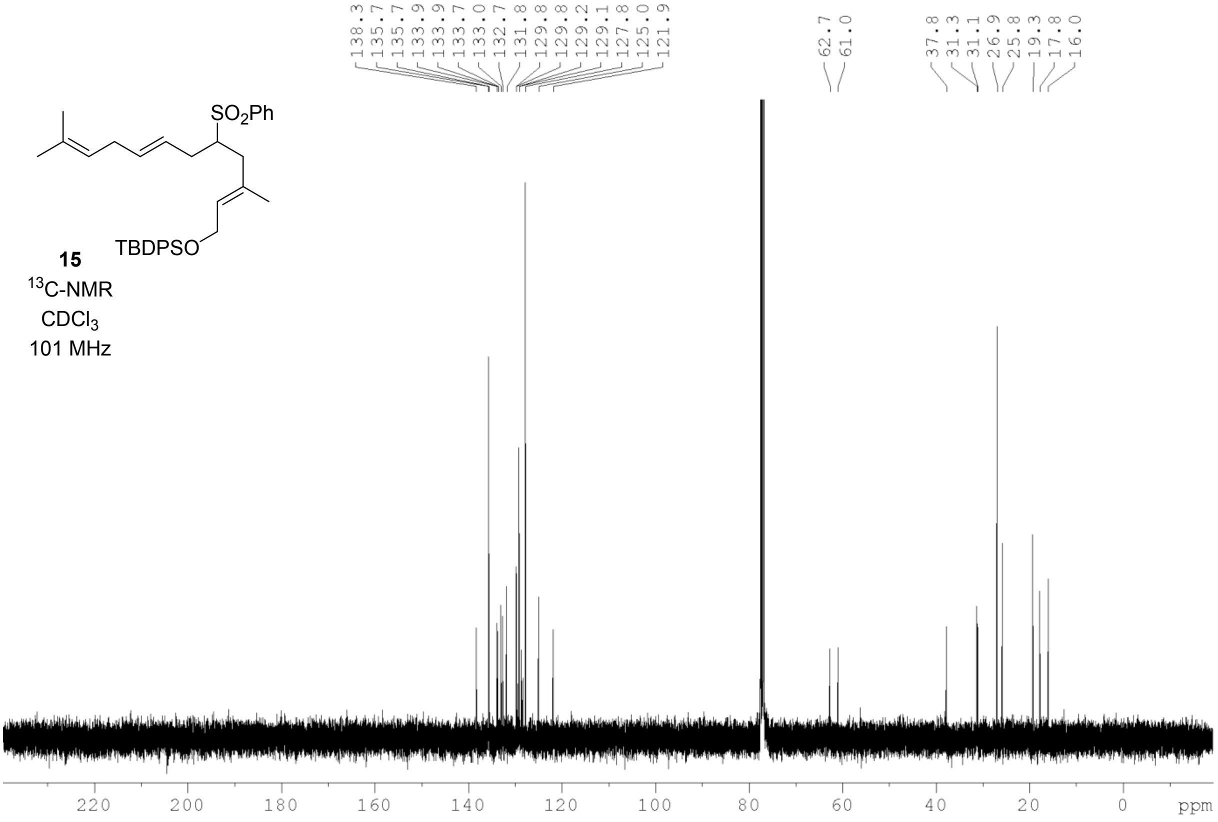
138.3 135.7 135.7 133.9 133.9 133.7 133.0 132.7 131.8 129.8 129.8 129.2 129.1 127.8 125.0 121.9
62.7 61.0
37.8 31.3 31.1 26.9 25.8 19.3 17.8 16.0
SO2Ph
TBDPSO
15
13C-NMR
CDCl3
101 MHz
220 200 180 160 140 120 100 80 60 40 20 0 ppm

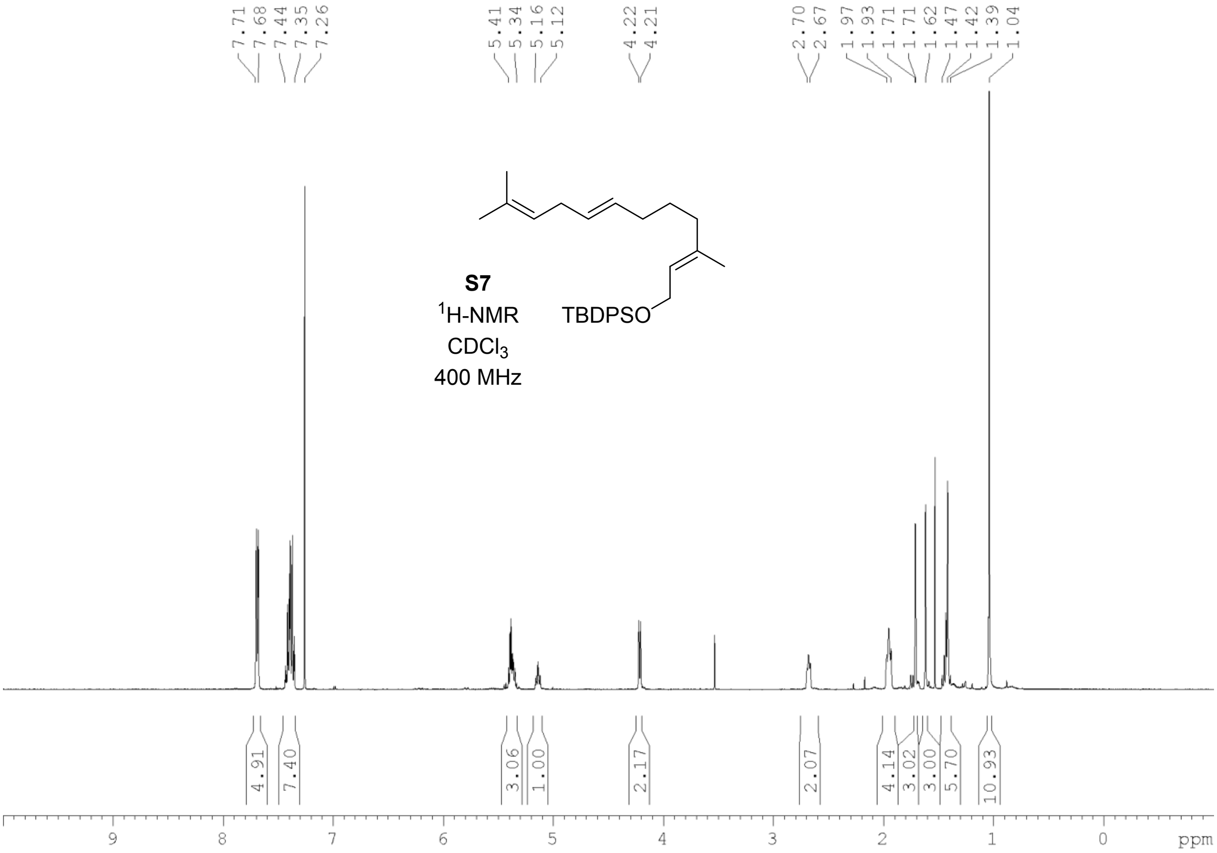
S7
1H-NMR
CDCl3
400 MHz
TBDPSO
7.71 7.68 7.44 7.35 7.26
5.41 5.34 5.16 5.12
4.22 4.21
2.70 2.67 1.97 1.93 1.71 1.71 1.62 1.47 1.42 1.39 1.04
4.91 7.40 3.06 1.00 2.17 2.07 4.14 3.02 3.00 5.70 10.93
9 8 7 6 5 4 3 2 1 0 ppm

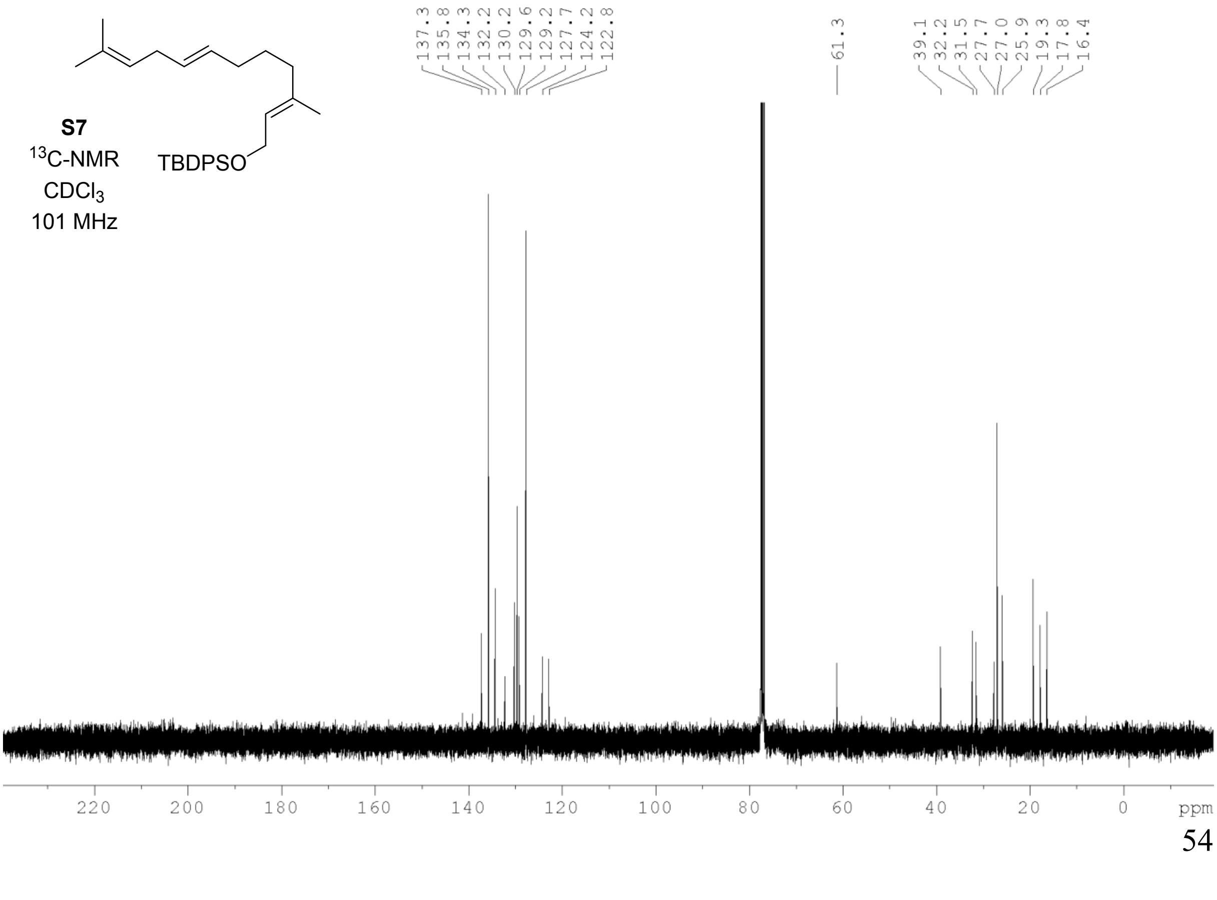
S7
13C-NMR
CDCl3
101 MHz
TBDPSO
137.3 135.8 134.3 132.2 130.2 129.6 129.2 127.7 124.2 122.8
61.3
39.1 32.2 31.5 27.7 27.0 25.9 19.3 17.8 16.4
220 200 180 160 140 120 100 80 60 40 20 0 ppm

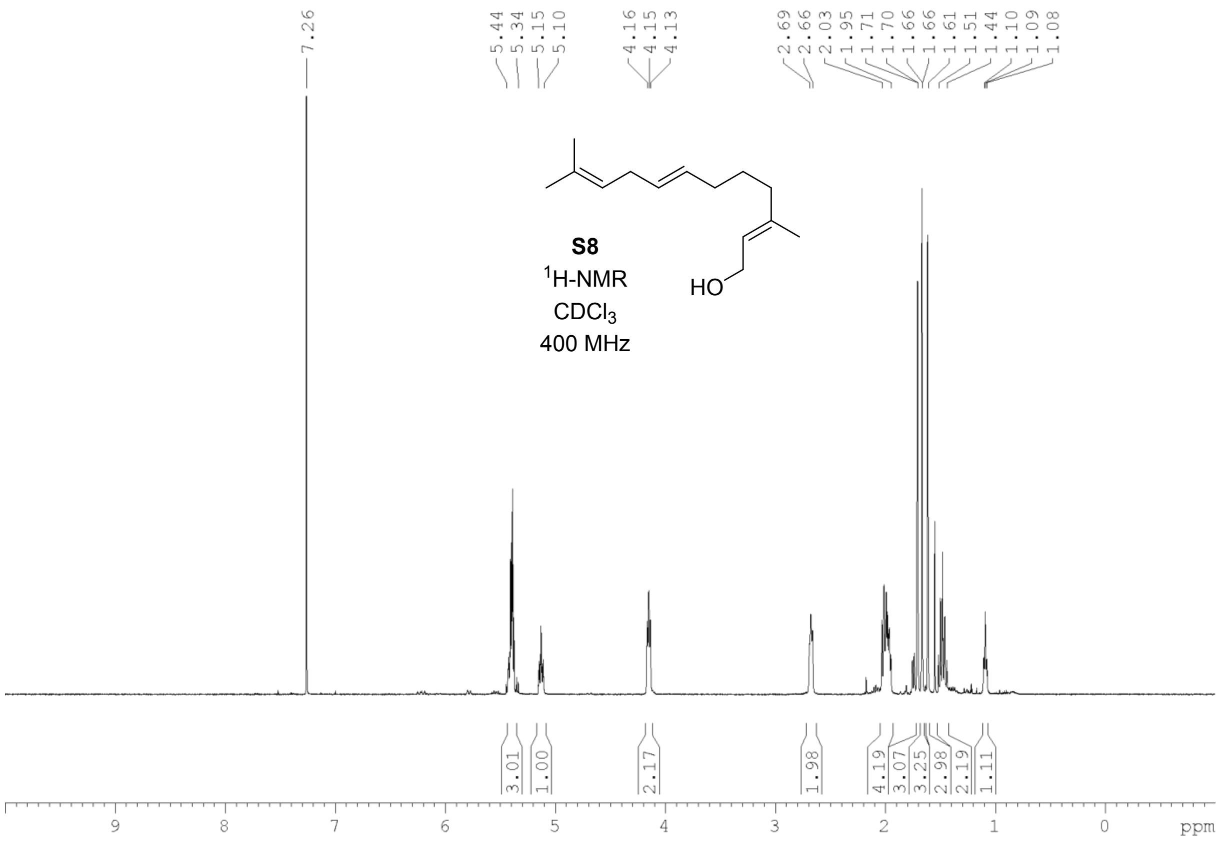
7.26
5.44
5.34
5.15
5.10
4.16
4.15
4.13
2.69
2.66
2.03
1.95
1.71
1.70
1.66
1.66
1.61
1.51
1.44
1.10
1.09
1.08
S8
^{1}H-NMR
$CDCl_3$
400 MHz
HO
3.01
1.00
2.17
1.98
4.19
3.07
3.25
2.98
2.19
1.11
9
8
7
6
5
4
3
2
1
0
ppm

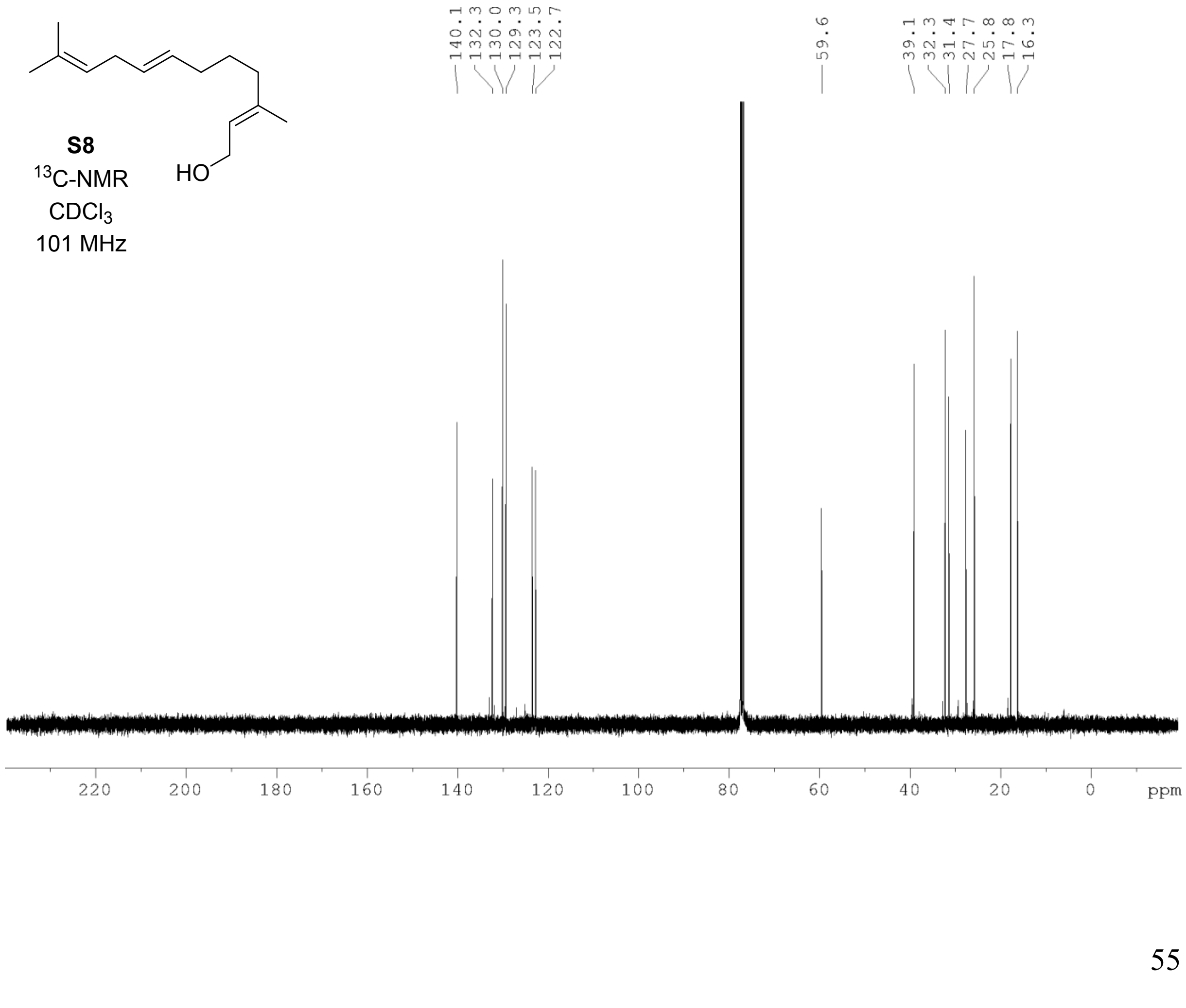
140.1
132.3
130.0
129.3
123.5
122.7
59.6
39.1
32.3
31.4
27.7
25.8
17.8
16.3
S8
^{13}C-NMR
$CDCl_3$
101 MHz
HO
220
200
180
160
140
120
100
80
60
40
20
0
ppm

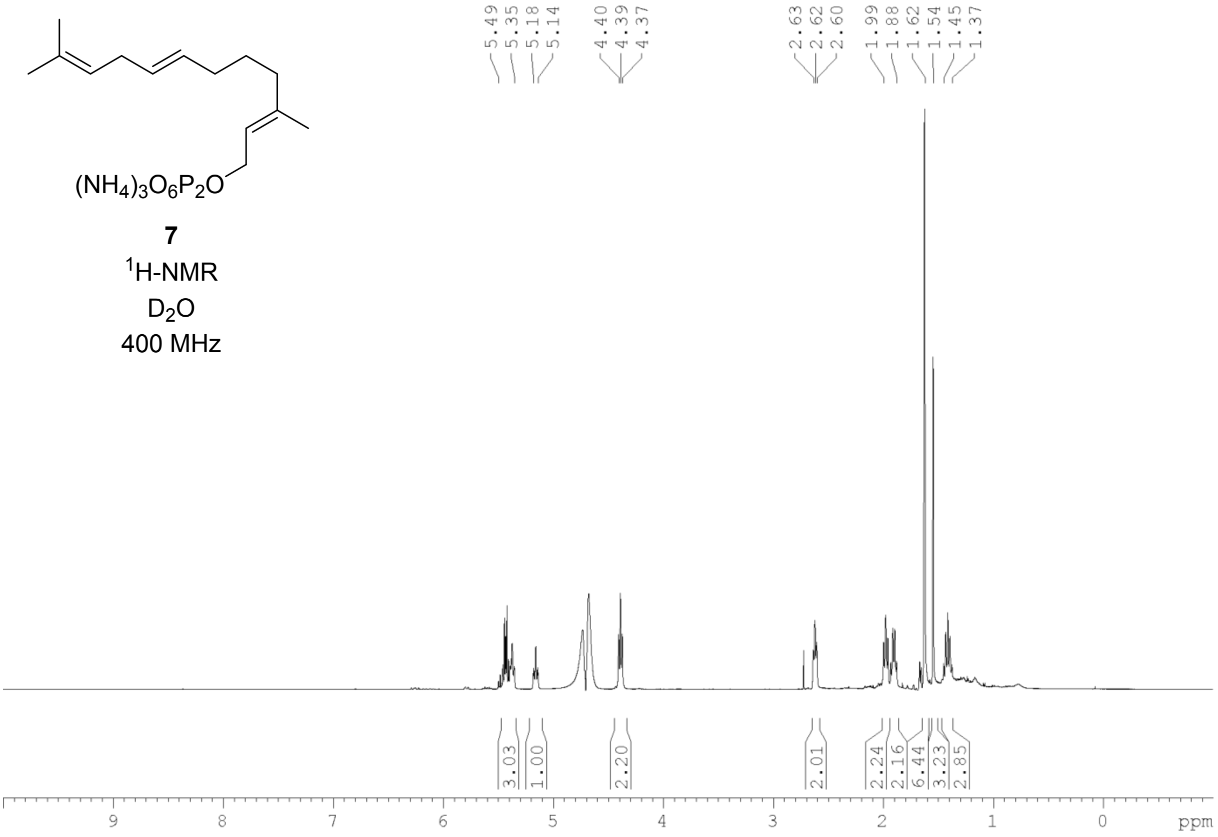

7
^{1}H-NMR
D_2O
400 MHz

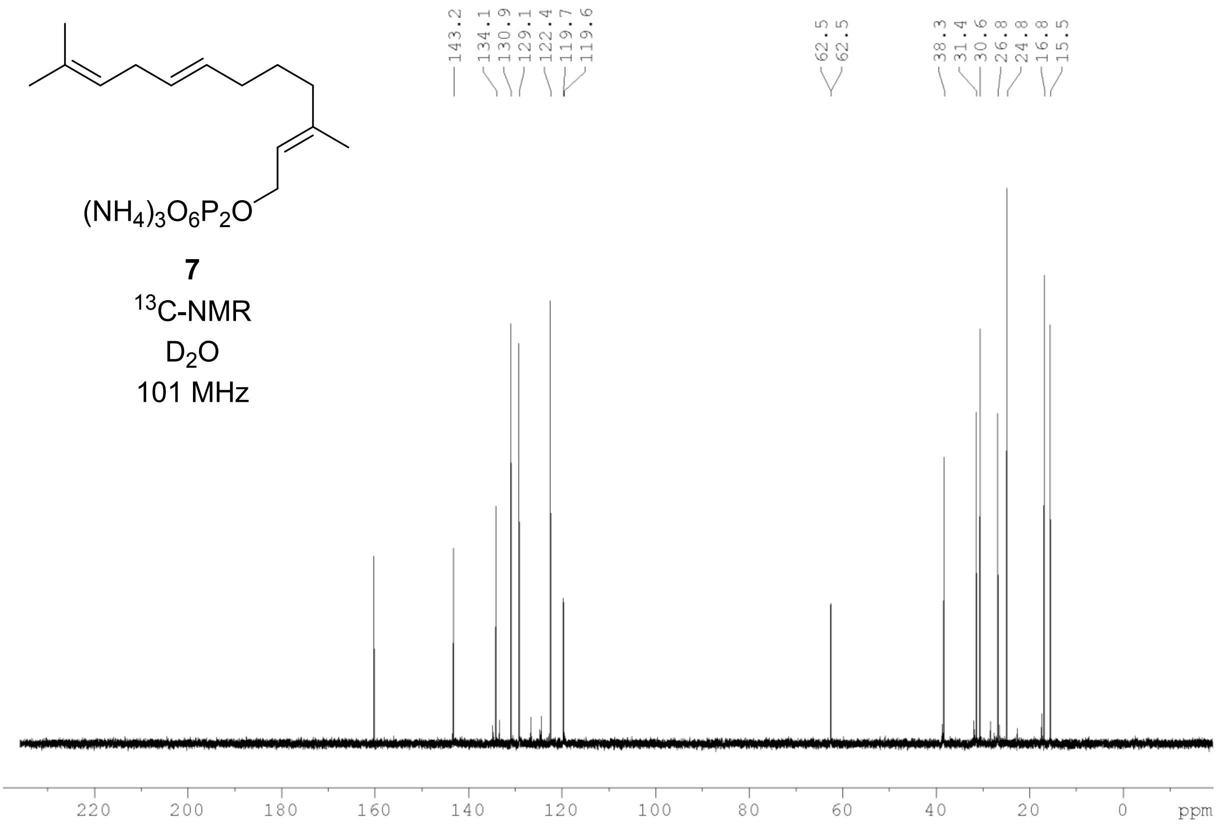

7
^{13}C-NMR
D_2O
101 MHz

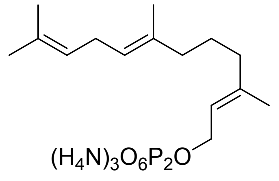

8
^{1}H-NMR
D_2O
400 MHz

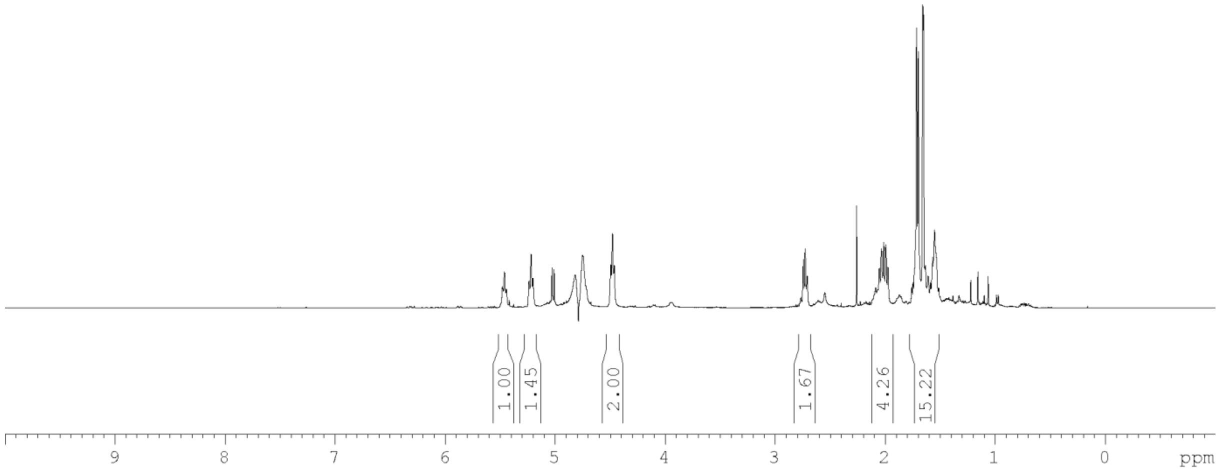

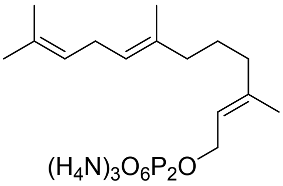

8
^{13}C-NMR
D_2O
101 MHz

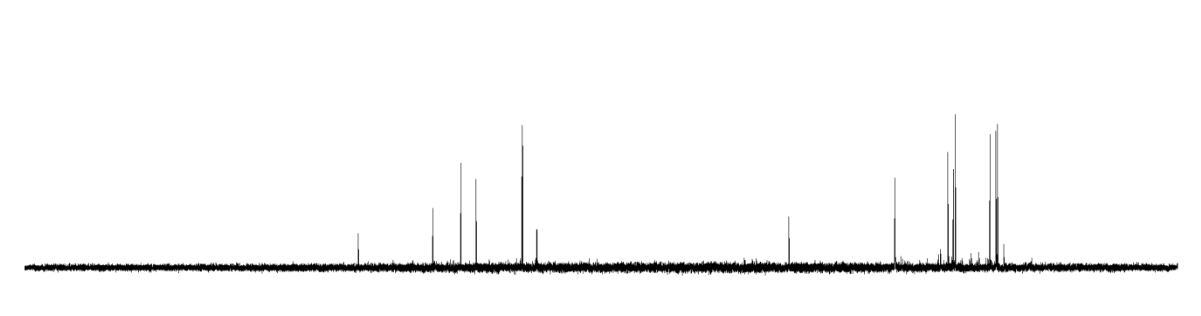

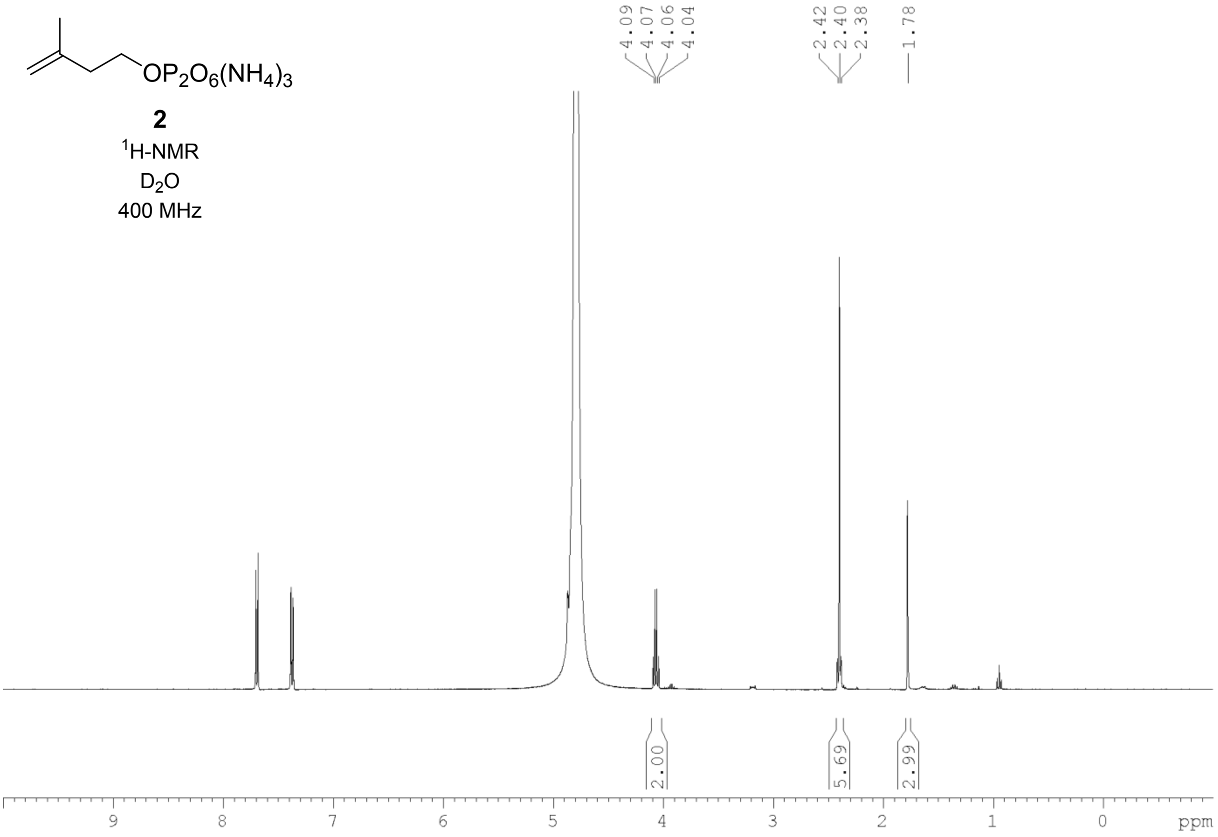

OP2O6(NH4)3
2
1H-NMR
D2O
400 MHz
4.09
4.07
4.06
4.04
2.42
2.40
2.38
1.78
2.00
5.69
2.99
9 8 7 6 5 4 3 2 1 0 ppm

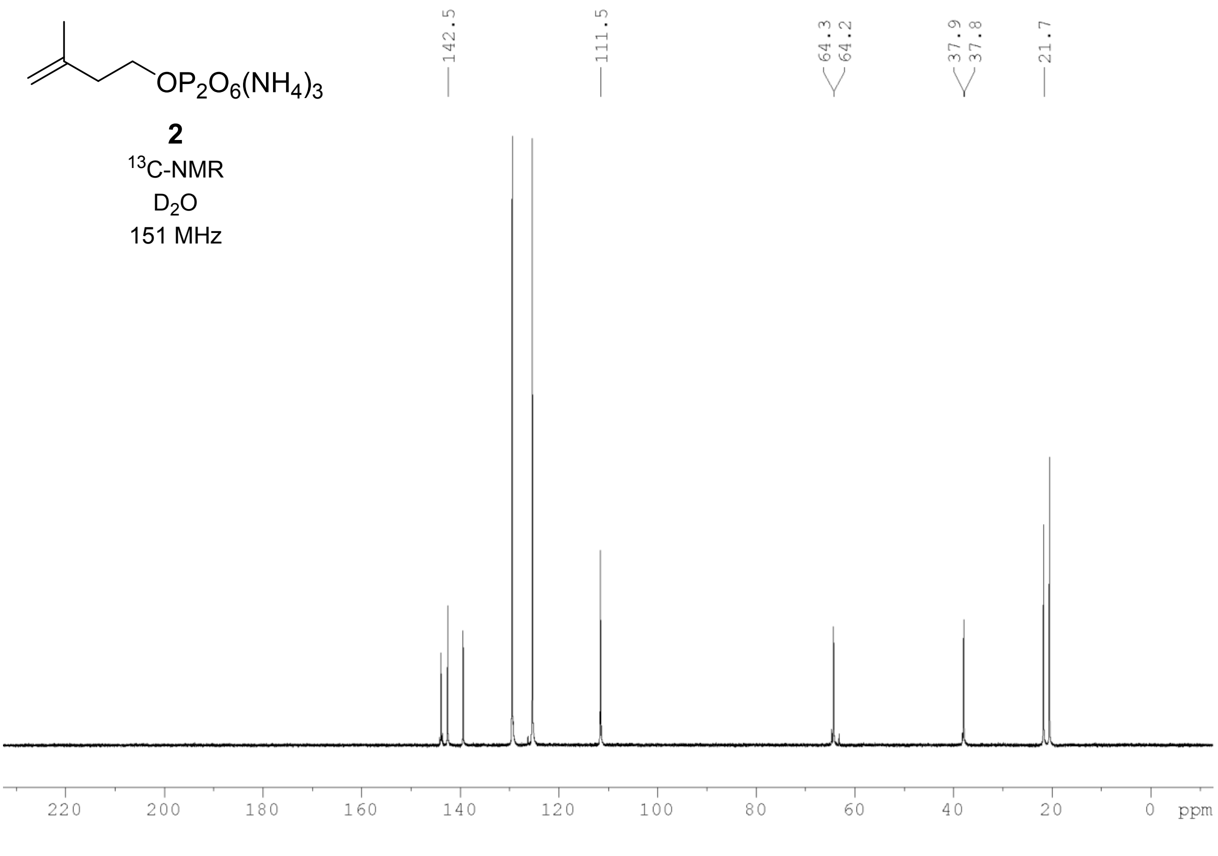

OP2O6(NH4)3
2
13C-NMR
D2O
151 MHz
142.5
111.5
64.3
64.2
37.9
37.8
21.7
220 200 180 160 140 120 100 80 60 40 20 0 ppm

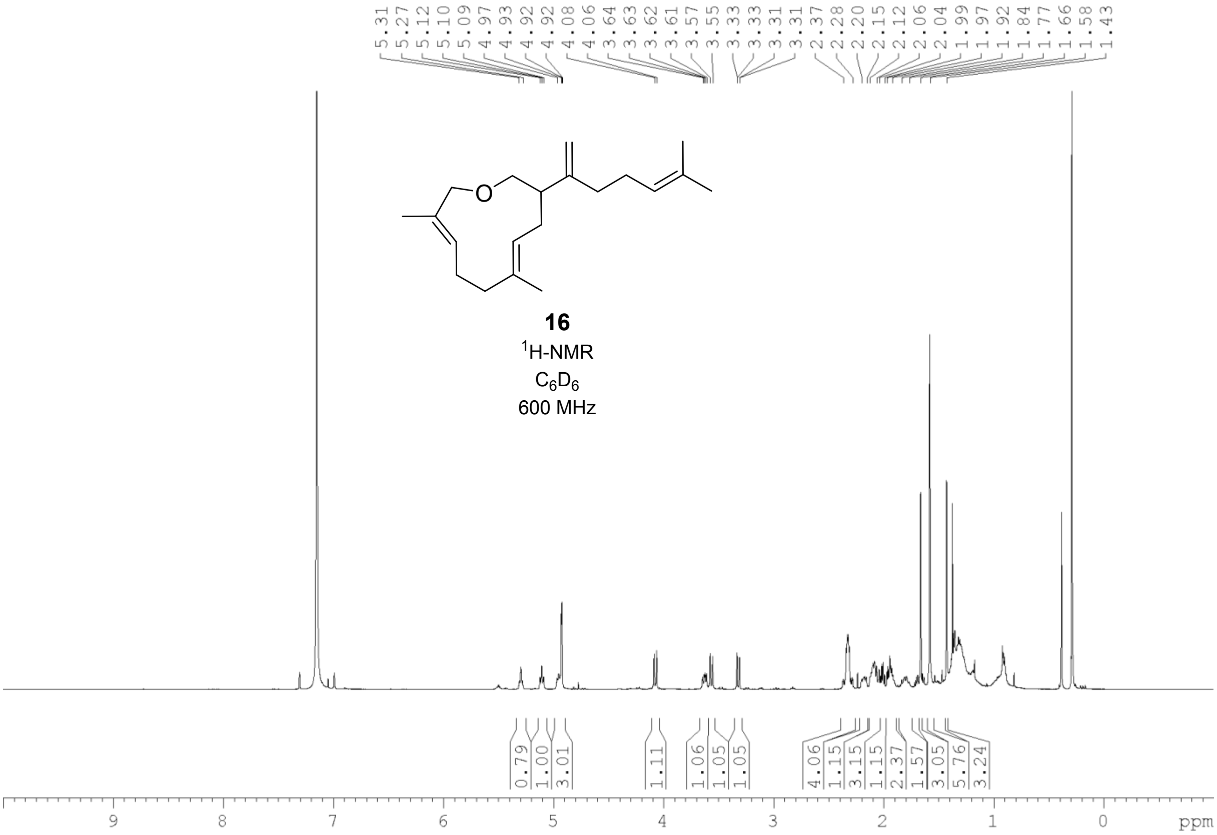
5.31 5.27 5.12 5.10 5.09 4.97 4.93 4.92 4.92 4.08 4.06 3.64 3.63 3.62 3.61 3.57 3.55 3.33 3.33 3.31 3.31 2.37 2.28 2.20 2.15 2.12 2.06 2.04 1.99 1.97 1.92 1.84 1.77 1.66 1.58 1.43
O
16
1H-NMR
C6D6
600 MHz
0.79 1.00 3.01 1.11 1.06 1.05 1.05 4.06 1.15 3.15 1.15 2.37 1.57 3.05 5.76 3.24
9 8 7 6 5 4 3 2 1 0 ppm

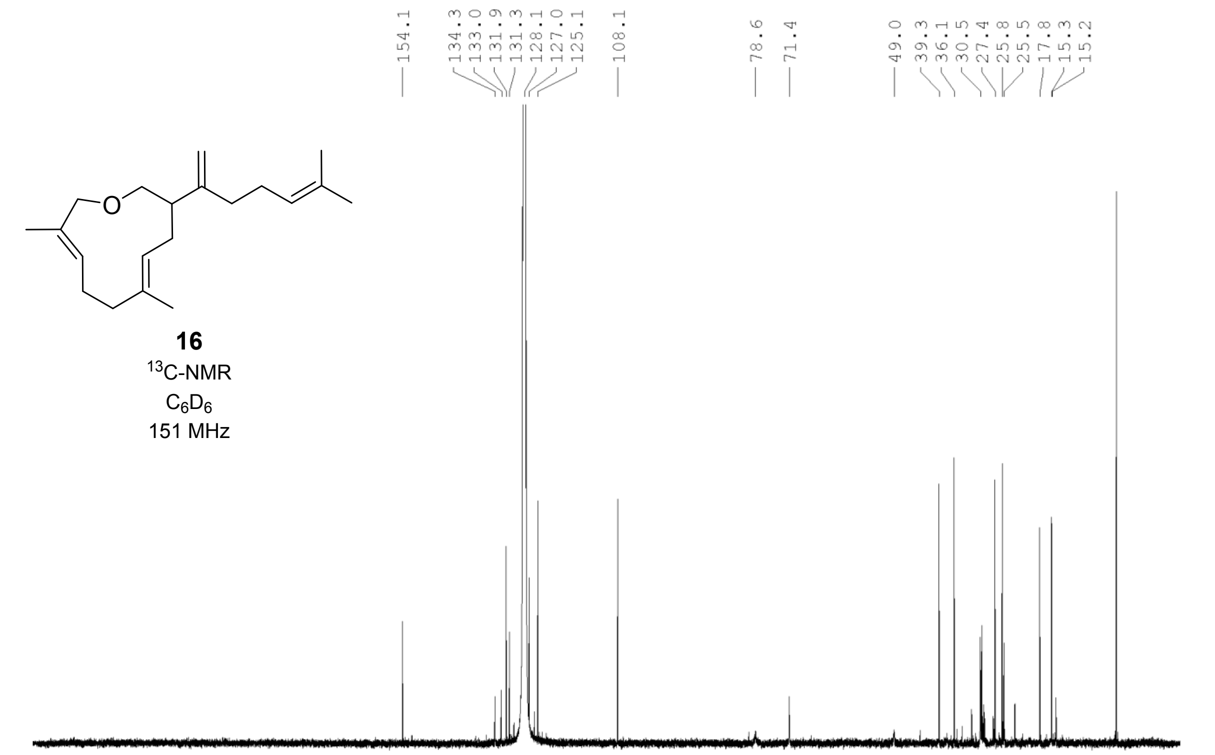
154.1 134.3 133.0 131.9 131.3 128.1 127.0 125.1 108.1 78.6 71.4 49.0 39.3 36.1 30.5 27.4 25.8 25.5 17.8 15.3 15.2
O
16
13C-NMR
C6D6
151 MHz
220 200 180 160 140 120 100 80 60 40 20 0 ppm

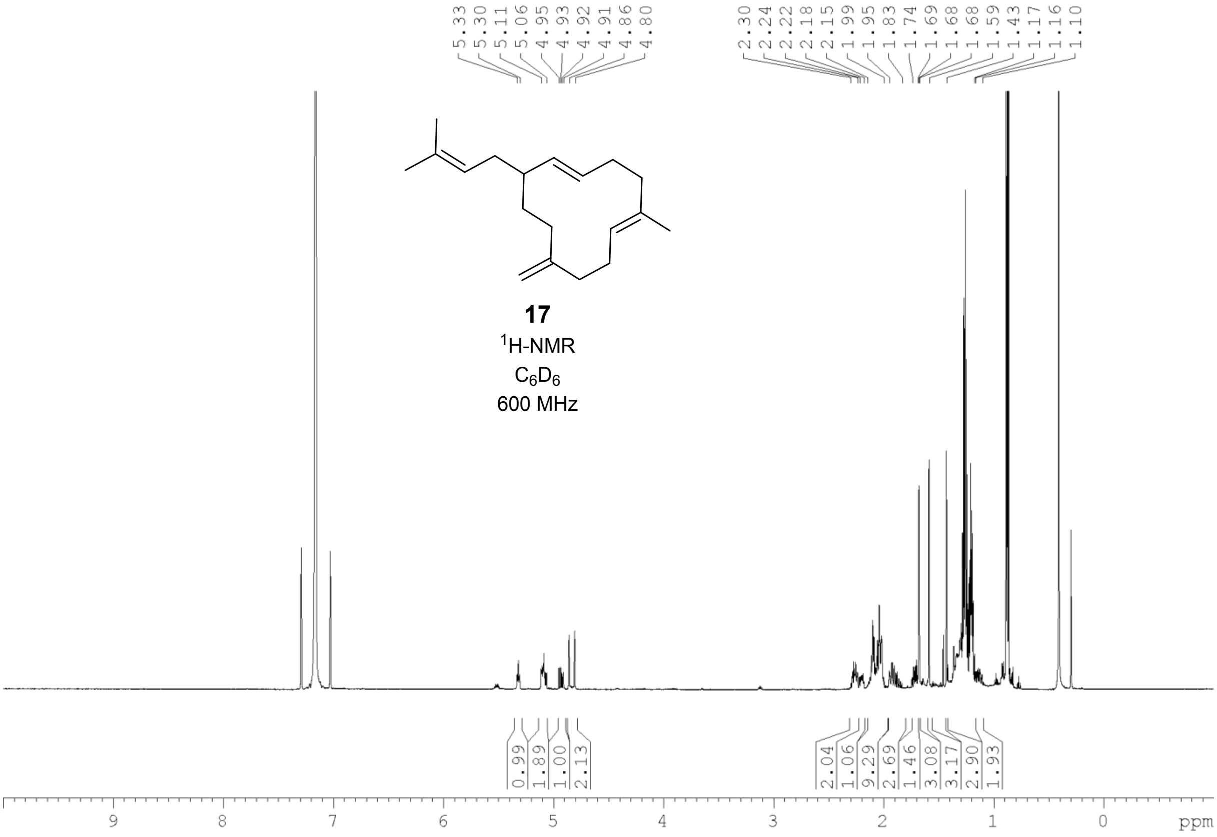
5.33
5.30
5.11
5.06
4.95
4.93
4.92
4.91
4.86
4.80
2.30
2.24
2.22
2.18
2.15
1.99
1.95
1.83
1.74
1.69
1.68
1.68
1.59
1.43
1.17
1.16
1.10
17
^{1}H-NMR
C_6D_6
600 MHz
0.99
1.89
1.00
2.13
2.04
1.06
9.29
2.69
1.46
3.08
3.17
2.90
1.93
9
8
7
6
5
4
3
2
1
0
ppm

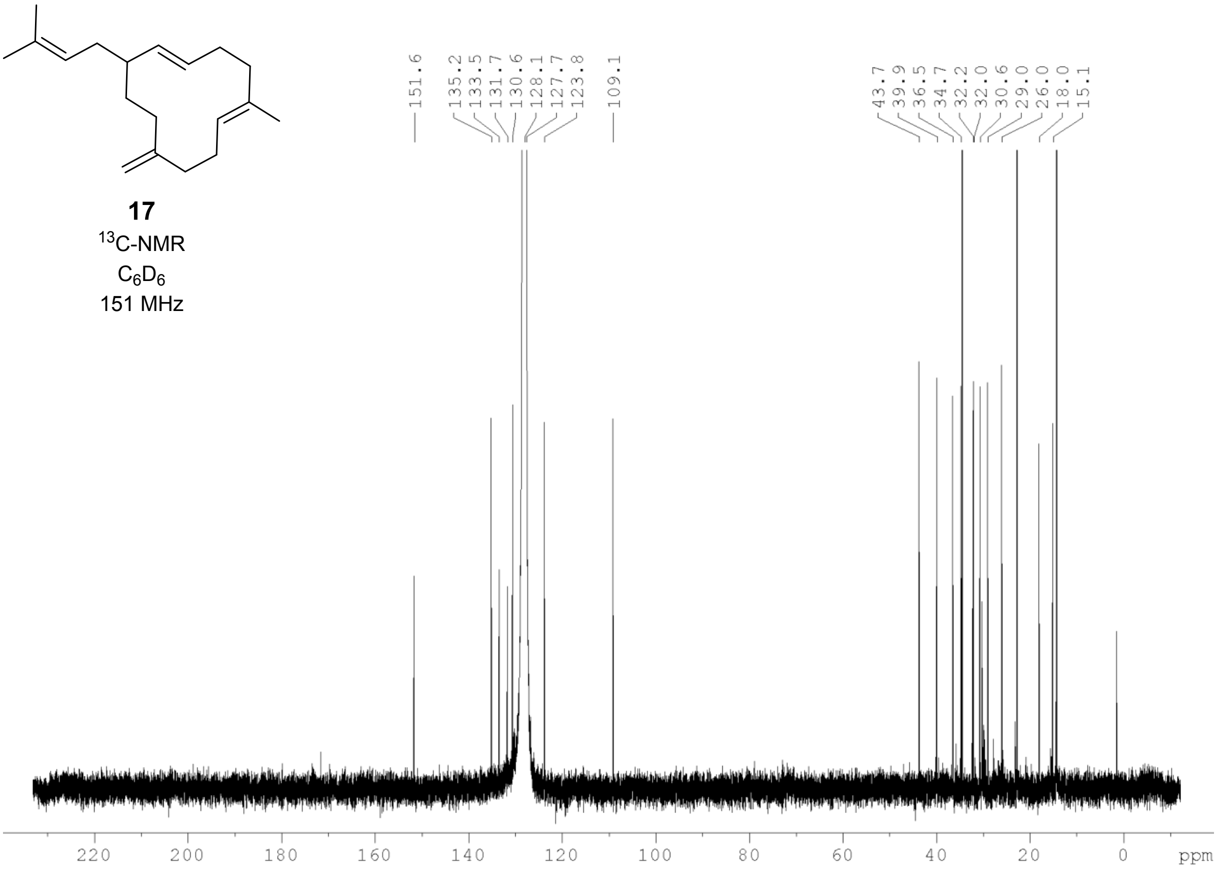
17
^{13}C-NMR
C_6D_6
151 MHz
151.6
135.2
133.5
131.7
130.6
128.1
127.7
123.8
109.1
43.7
39.9
36.5
34.7
32.2
32.0
30.6
29.0
26.0
18.0
15.1
220
200
180
160
140
120
100
80
60
40
20
0
ppm

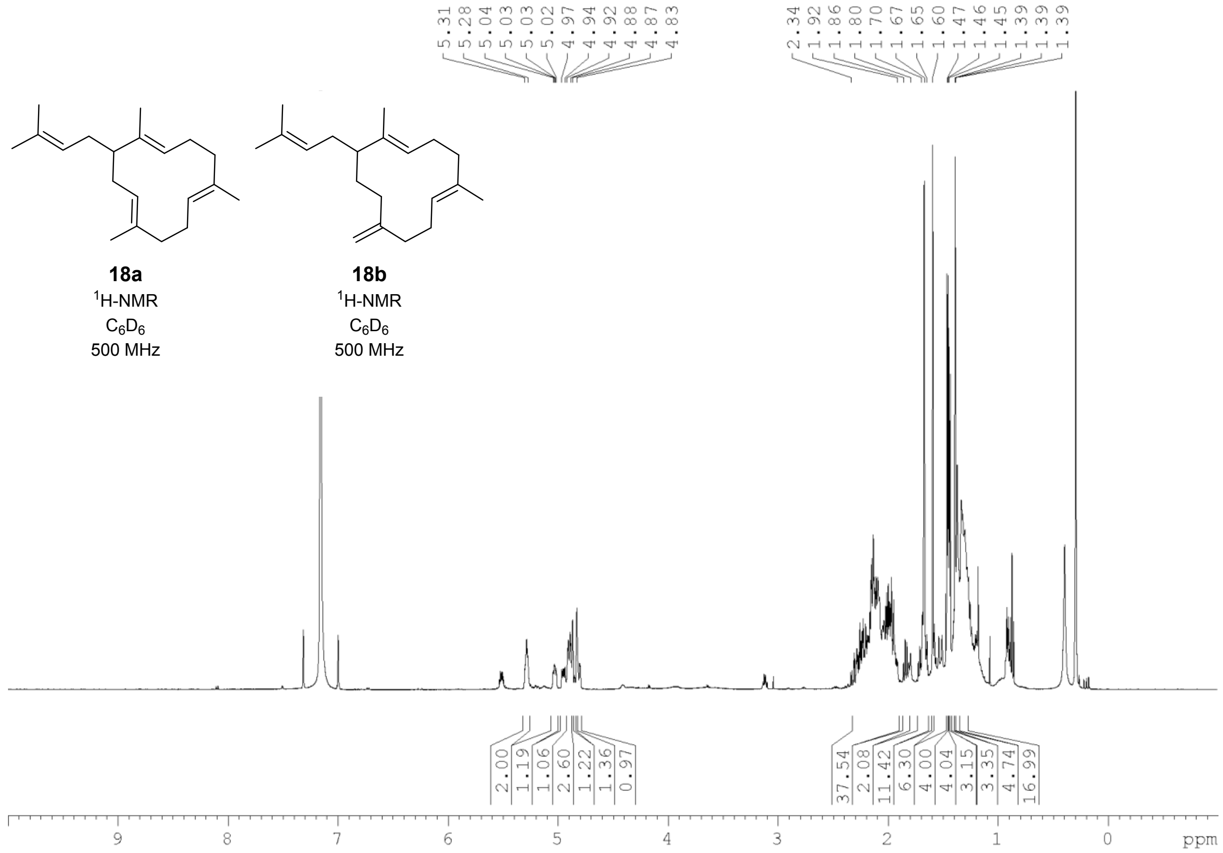
5.31
5.28
5.04
5.03
5.02
4.97
4.94
4.92
4.88
4.87
4.83
2.34
1.92
1.86
1.80
1.70
1.67
1.65
1.60
1.47
1.46
1.45
1.39
1.39
1.39
18a
1H-NMR
C6D6
500 MHz
18b
1H-NMR
C6D6
500 MHz
2.00
1.19
1.06
2.60
1.22
1.36
0.97
37.54
2.08
11.42
6.30
4.00
4.04
3.15
3.35
4.74
16.99
9 8 7 6 5 4 3 2 1 0 ppm

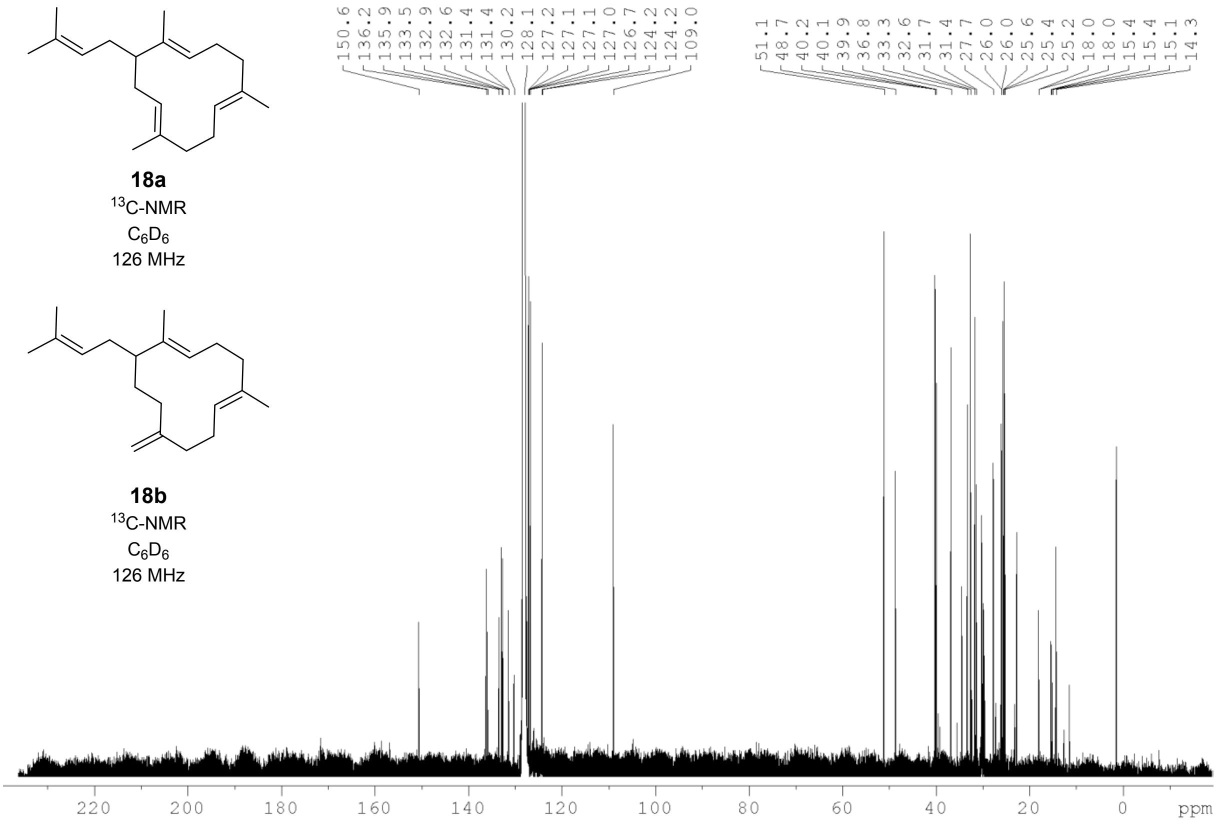
150.6
136.2
135.9
133.5
132.9
132.6
131.4
131.4
130.2
128.1
127.2
127.1
127.1
127.0
126.7
124.2
124.2
109.0
51.1
48.7
40.2
40.1
39.9
36.8
33.3
32.6
31.7
31.4
27.7
26.0
26.0
25.6
25.4
25.2
18.0
18.0
15.4
15.1
14.3
18a
13C-NMR
C6D6
126 MHz
18b
13C-NMR
C6D6
126 MHz
220 200 180 160 140 120 100 80 60 40 20 0 ppm

3. References (supporting information)

S1 Oberhauser, C.; Harms, V.; Seidel, K.; Schröder, B.; Ekramzadeh, K.; Beutel, S.; Winkler, S.; Lauterbach, L.; Dickschat, J. S.; Kirschning, A., *Angew. Chem. Int. Ed.* **2018**, *57*, 11802-11806.

S2 Struwe, H.; Droste, J.; Dhar, D.; Davari, M. D.; Kirschning, *ChemBioChem* **2023**, e202300599.

S3 Braddock, D. C.; Bhuva, R.; Millan, D. S.; Pérez-Fuertes, Y.; Roberts, C. A.; Sheppard, R. N.; Solanki, S.; Stokes E. S. E.; White, A. J. P., *Org. Lett.* **2007**, *9*, 445 – 448.

S4 Wang, C.; Barluenga, S.; Koripelly, G. K.; Fontaine, J.-G.; Chen, R.; Yu, J.-C.; Shen, X.; Chabala, J. C.; Heck, J. V.; Rubenstein, A.; Wissinger, N., *Bioorg. Med. Chem. Lett.* **2009**, *19*, 3836 – 3840.

S5 Fiasella, A.; Nuzzi, A.; Summa, M.; Armirotti, A.; Tarozzo, Tarzia, G.; Mor, M.; Bertozzi, F.; Bandiera, T.; Piomelli, D., *ChemMedChem* **2014**, *9*, 1602 – 1614.

S6 Hou, A.; Dickschat, J. S., *Angew. Chem. Int. Ed.* **2020**, *59*, 19961 – 19965.
